# Design, synthesis, in vitro biological assessment and molecular modeling insights for novel 3-(naphthalen-1-yl)-4,5-dihydropyrazoles as anticancer agents with potential EGFR inhibitory activity

**DOI:** 10.1038/s41598-022-15050-8

**Published:** 2022-07-27

**Authors:** Wagdy M. Eldehna, Mahmoud A. El Hassab, Zainab M. Elsayed, Tarfah Al-Warhi, Hazem Elkady, Mahmoud F. Abo-Ashour, Mohammed A. S. Abourehab, Ibrahim H. Eissa, Hatem A. Abdel-Aziz

**Affiliations:** 1 Department of Pharmaceutical Chemistry, Faculty of Pharmacy, Kafrelsheikh University, Kafrelsheikh, 33516 Egypt; 2 School of Biotechnology, Badr University in Cairo, Badr City, Cairo 11829 Egypt; 3 Department of Medicinal Chemistry, Faculty of Pharmacy, King Salman International University (KSIU), Ras Sedr, South Sinai Egypt; 4 Scientific Research and Innovation Support Unit, Faculty of Pharmacy, Kafrelsheikh University, Kafrelsheikh, Egypt; 5 Department of Chemistry, College of Science, Princess Nourah Bint Abdulrahman University, P.O. Box 84428, Riyadh, 11671 Saudi Arabia; 6 Pharmaceutical Medicinal Chemistry and Drug Design Department, Faculty of Pharmacy (Boys), Al-Azhar University, Cairo, 11884 Egypt; 7 Department of Pharmaceutical Chemistry, Faculty of Pharmacy, El saleheya El Gadida University, El Saleheya El Gadida, Egypt; 8 Department of Pharmaceutics, Faculty of Pharmacy, Umm Al-Qura University, Makkah, 21955 Saudi Arabia; 9 Department of Applied Organic Chemistry, National Research Center, P.O. Box 12622, Dokki, Giza, Egypt

**Keywords:** Medicinal chemistry, Medicinal chemistry, Oncology

## Abstract

Currently, the humanity is in a fierce battle against various health-related challenges especially those associated with human malignancies. This created the urge to develop potent and selective inhibitors for tumor cells through targeting specific oncogenic proteins possessing crucial roles in cancer progression and survive. In this respect, new series of pyrazole-thiazol-4-one hybrids (**9a–p**) were synthesized as potential anticancer agents. All the synthesized molecules exhibited potent antiproliferative actions against breast cancer (BC) T-47D and MDA-MB-231 cell lines with IC_50_ ranges 3.14–4.92 and 0.62–58.01, respectively. Moreover, the most potent anti-proliferative counterparts **9g** and **9k** were assessed against EGFR. They displayed nanomolar inhibitory activity, IC_50_ 267 ± 12 and 395 ± 17 nM, respectively. Worth noting, both compounds 9g and 9k induced apoptosis in MDA-MB-231 cells, and resulted in a cell cycle arrest at G2/M phase. Furthermore, an in silico analysis including docking and molecular dynamic simulations was performed.

## Introduction

In 2020, 2.3 million women were affected by breast cancer with nearly 685,000 deaths killed by the same disease worldwide. As the year ended, a report of the past 5 years, revealed 7.8 million alive women who diagnosed with breast cancer, giving it rise to be the most prevalent cancer globally^1^. More than 90% of BCs are not metastatic upon diagnosis. For breast cancer patients that diagnosed without metastatic disease, the main therapeutic objectives are eradication of the tumor and preventing recurrence. In many cases, patients with nonmetastatic breast cancer can be treated with chemotherapy as a systemic therapy^2^.

RTKs (receptor tyrosine kinases) are found on the cells surface and represent a type of receptor that plays a significant role in the genesis of tumors. RTKs are known to influence cancer stemness, angiogenesis, and metastasis through a variety of downstream signaling pathways^3^. RTKs are a good target for breast cancer treatment because of their several activities^4^. However, anti-RTK therapy is complicated by the mutations that are created in these biological targets^5^. Different types of RTKs are over-expressed in various human malignancies, including breast cancer, they are namely epidermal growth factor receptors (EGFRs), platelet-derived growth factor receptors (PDGFRs), vascular endothelial growth factor receptors (VEGFRs), fibroblast growth factor receptors (FGFRs) and insulin-like growth factor receptors (IGFRs)^3,6–8^. Overexpression of RTKs are associated with increased breast cancer aggressiveness^8^.

EGFR belongs to the ErbB family of RTK. These proteins are stimulated after binding with EGF-family of proteins. Many reports confirmed that the EGFR is involved in the pathogenesis of different cancer types^9^. The EGFR and its agonists are over-expressed in human malignancies producing cell transformation^10^. All EGFR types are expressed in breast cancer. EGFR has been reported to be over-expressed in about 14% of human breast tumors^11^.

**Figure 1:**
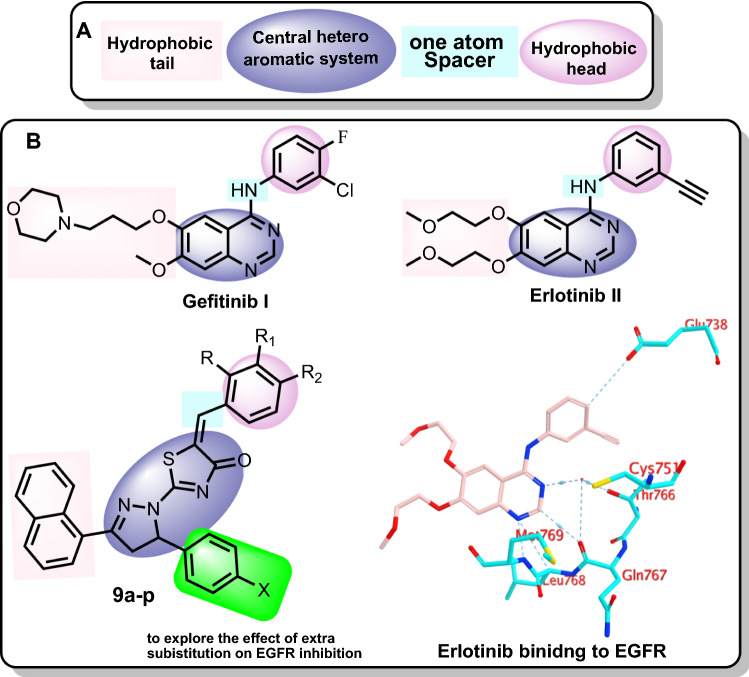
(**A**) The reported pharmacophoric features of EGFRIs. (**B**) Reported FDA EGFR inhibitors (Gefitinib and Erlotinib) and the synthesized compounds in this study (**9a-p**), as well as the binding mode of Erlotinib within EGFR active site.

Many EGFRs small molecule inhibitors (EGFRIs) have been developed. In comparison to other ErbB receptors, Gefitinib **I** (Fig. 1) is a reversible EGFRI with a 200-fold higher affinity for EGFR. It's been approved for the management of individuals with breast tumors and NSCLC who haven't responded to chemotherapies^12,13^. Erlotinib **II** (Fig. 1) is a reversible EGFRI that has been approved by the FDA for the management of NSCLC and metastatic pancreatic malignancy^14^. Gefitinib as well as Erlotinib work as ATP analogues through competing with the EGFR receptors' ATP binding spaces^15^. Despite the fact that EGFRIs have significant clinical outcomes in treating patients with breast cancer, tumor cells rapidly develop acquired resistance, in which the efficacy of EGFR-targeted therapy is highly limited^16^ (Fig. 1).

As depicted by Fig. 1, some pharmacophoric features of EGFR inhibitors are required for maximum affinity against the EGFR ATP binding site. These features include: (1) a central flat aromatic heterocyclic moiety that should be accommodated within the adenine binding pocket to interact with Leu768, Met769, Gln767 and Thr766^17^, (2) a terminal hydrophobic head which should occupy the hydrophobic region I to interact with Glu738, (3) a spacer of one atom (NH, spacer) to allow the terminal hydrophobic motif to access the hydrophobic region I^18^, (4) a hydrophobic tail that can occupy the hydrophobic region II^19,20^. Research about the ribose binding pocket has been limited^21^ (Fig. 1).

Literature investigation revealed that pyrazole moiety is a lead building block in many anticancer molecules through the inhibition of diverse RTKs including EGFR^22,23^. In addition, thiazol-4(5*H*)-one was reported as promising scaffold in the discovery of new antineoplastic agents targeting RTKs especially EGFR^22,24^.

Accordingly, the aim of this study is summarized on the design and synthesis of pyrazole- thiazol-4(5*H*)-one hybrids (**9a-p**, Fig. 1) having the essential pharmacophoric features of EGFRIs. In such design, the 2-(4,5-dihydro-1*H*-pyrazol-1-yl)thiazol-4-one moiety was utilized as an aromatic heterocyclic system to be convenient for the adenine binding pocket. In addition, diverse substituted phenyl rings were used as a hydrophobic head to accommodate within the hydrophobic pocket. Furthermore, the naphthyl moiety was used as a hydrophobic tail to occupy a second hydrophobic pocket. In this design, we gave two modifications on the reported EGFRIs. The first one, the NH linker was replaced by –CH= group as a chemical isostere. The second modification was the grafting of different aromatic substituents to explore the effect of such substituents on the biological activities (Fig. 1).

The synthesized molecules were examined as anti-proliferative agents against 2 BC MDA-MB-231 and T-47D cell lines. Then, the most promising members that showed superior cytotoxic activity were further evaluated for their EGFR inhibitory activity. In addition, the effects of these active members against cell cycle and apoptosis induction were examined. Finally, Molecular modelling studies including docking and molecular dynamic simulations were carried out to examine the binding mode of herein reported pyrazole- thiazol-4(5*H*)-one hybrids against their proposed biological target (EGFR).

## Results and discussion

### Chemistry

**Scheme 1:**
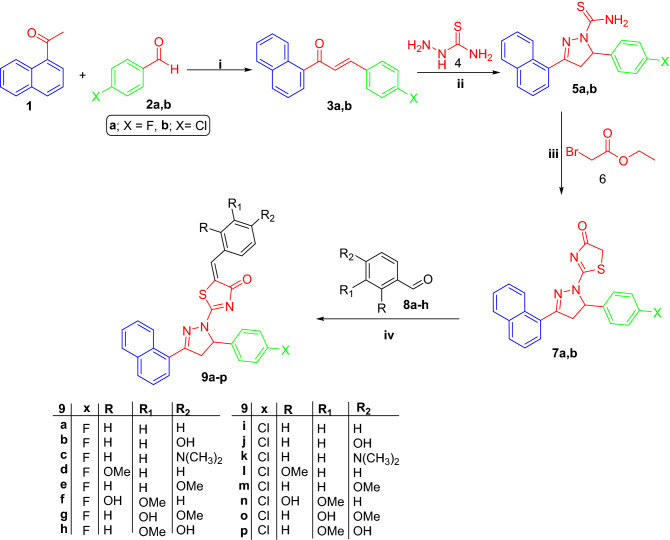
Synthesis of target molecules **9a-p**; (**i**) 40% sodium hydroxide, 95% ethyl alcohol, stirring at R.T. 8 h; (**ii**) Sodium hydroxide, ethyl alcohol, reflux 2 h; (**iii**) Absolute ethyl alcohol, sodium acetate, reflux 4 h; (**iv**) Glacial AcOH, CH_3_COONa, reflux 6 h.

The adopted strategy to prepare the herein proposed pyrazole- thiazol-4(5*H*)-one hybrids **9a-p** was illustrated in Scheme 1. 1-(Naphthalen-1-yl)ethan-1-one **1** was condensed with the appropriate 4-fluorobenzaldehyde **2a** or 4-chlorobenzaldehyde **2b** in a basic medium through the Claisen-Schmidt condensation reaction to furnish 3-(4-fluorophenyl)-1-(naphthalen-1-yl)propen-1-one **3a** and 3-(4-chlorophenyl)-1-(naphthalen-1-yl)propen-1-one **3b**, respectively.

Chalcones **3a-b** were then subjected to heterocyclization to produce 3-(naphthalen-1-yl)-4,5-dihydro-pyrazole-1-carbothioamide derivatives **5a-b** via refluxing in absolute ethanol with thiosemicarbazide and sodium hydroxide. The previously prepared thioamide compounds **5a-b** were heated under reflux in absolute ethanol with ethyl bromoacetate **6** to get the two key intermediates 3-(naphthalen-1-yl)-4,5-dihydropyrazol-1-yl)thiazol-4(5*H*)-one derivatives **7a-b**. Finally, the pyrazole- thiazol-4(5*H*)-one intermediates **7a-b** were reacted with different aldehydes **8a-h** via the Knoevenagel condensation by heating in glacial acetic acid and fused sod. acetate to yield the targeted pyrazole- thiazol-4(5*H*)-one hybrids **9a-p**.

Spectral and elemental analyses data supported the proposed structure for target pyrazole- thiazol-4(5*H*)-one hybrids **9a-p** herein reported. ^1^H NMR spectra for pyrazolines **9a-p** showed three doublet of doublet signals of pyrazoline ring; one for CH and two for CH_2_ about *δ* (5.90) and (3.67 and 4.39) *ppm*, respectively with three reciprocal values for coupling constant (4.0, 11.2, 18.0) Hz. Moreover, ^1^H NMR spectra for compounds **9d-h** and **9l-p** confirmed their structure through the presence of additional aliphatic singlet signal for OCH_3_ around *δ* 3.80 ppm, whereas, the ^1^H NMR spectra of pyrazole- thiazol-4-one hybrids **9c** and **9k** revealed the presence of another aliphatic signal for (N(CH_3_)_2_) around *δ* 3.01 ppm*.* In turn, the ^13^C NMR spectra for the targeted pyrazole- thiazol-4-one hybrids **9a-p** disclosed the presence of aliphatic signals of pyrazoline ring around (46.55 and 62.40) *ppm*, for CH_2_ and CH, respectively, in addition, the signal of (C = O) functional group appeared around *δ* 179.90 ppm*.* Lastly, the ^13^C NMR spectra of pyrazolines **9d-h** and **9l-p** disclosed the presence of the methoxy group around *δ* 56.0 ppm*.*

### Biological evaluation

#### In vitro anti-proliferative activity

The cytotoxic effects of herein prepared pyrazole- thiazol-4-one hybrids **9a-p** against BC MDA-MB-231 and T-47D cell lines, obtained from American Type Culture Collection (ATCC), were determined using the MTT assay protocol. It is worth to mention that the selected cancer cell lines in this study are EGFR-expressing cell lines^25–28^. Doxorubicin and Erolitinib were utilized as positive control (Table 1).

**Table 1:** Anti-proliferative activity of pyrazolines **9** BC MDA-MB-231 and T-47D cancer cell lines, as well as towards breast MCF-10A normal cells.


Com	X	R	R_1_	R_2_	IC_50_ (µM)^a^		
					MDA-MB-231	T-47D	MCF-10A
**9a**	F	H	H	H	19.84 ± 1.08	NA^b^	ND^c^
**9b**	F	H	H	OH	12.24 ± 0.67	18.61 ± 0.87	ND^c^
**9c**	F	H	H	N(CH_3_)_2_	2.54 ± 0.138	8.45 ± 0.27	47.03 ± 3.52
**9d**	F	OMe	H	H	45.02 ± 2.45	NA^b^	ND^c^
**9e**	F	H	H	OMe	14.13 ± 0.77	12.37 ± 0.33	ND^c^
**9f.**	F	OH	OMe	H	3.40 ± 0.185	27.1 ± 1.08	42.60 ± 2.81
**9g**	F	H	OH	OMe	0.62 ± 0.03	3.14 ± 0.11	31.74 ± 3.10
**9h**	F	H	OMe	OH	5.82 ± 0.317	11.42 ± 0.67	79.45 ± 5.27
**9i**	Cl	H	H	H	24.68 ± 1.35	NA^b^	ND^c^
**9j**	Cl	H	H	OH	6.24 ± 0.34	35.10 ± 2.04	65.37 ± 5.04
**9k**	Cl	H	H	N(CH_3_)_2_	1.14 ± 0.06	4.92 ± 0.28	58.51 ± 4.36
**9l**	Cl	OMe	H	H	58.01 ± 3.16	NA^b^	ND^c^
**9m**	Cl	H	H	OMe	18.95 ± 1.03	27.63 ± 1.24	ND^c^
**9n**	Cl	OH	OMe	H	35.15 ± 1.916	67.29 ± 2.51	ND^c^
**9o**	Cl	H	OH	OMe	10.81 ± 0.59	7.36 ± 0.30	ND^c^
**9p**	Cl	H	OMe	OH	6.55 ± 0.357	19.47 ± 0.92	83.62 ± 6.74
**Doxorubicin**	–	–	–	–	3.67 ± 0.20	2.73 ± 0.09	ND^c^
**Erolitinib**	–	–	–	–	2.88 ± 0.17	3.61 ± 0.24	

^a^IC_50_ value is the mean ± S.D. of 3 separate experiments.

^b^NA: Derivatives with IC_50_ more than 100 mM.

^c^ND: Not determined.

Generally, the obtained results disclosed that MDA-MB-231 cell line was more susceptible to the influence of the tested members than T-47D. Also, it was noticed that the 4-fluorophenyl derivative **9g (**IC_50_ = 0.62 ± 0.03 against MDA-MB-231 and 3.14 ± 0.11 µM against T-47D) and the 4-chlorophenyl derivative **9k (**IC_50_ = 1.14 ± 0.06 against MDA-MB-231 and 4.92 ± 0.28 µM against T-47D**)** were the most potent cytotoxic members against the two breast cancer cell lines, comparing to the positive control, doxorubicin (IC_50_ = 3.67 ± 0.2 and 2.73 ± 0.09 µM against MDA-MB-231 and T-47D, respectively), Table 1.

According to all results, it can be concluded that the 4-fluorophenyl derivatives **9a-h** were slightly more advantageous than the 4-chlorophenyl derivatives **9i-p** against the explor cell lines.

Regarding the activity toward MDA-MB-231 BC cell line, hybrids **9c**, **9f**, **9h**, **9j** and **9p** displayed good cytotoxicity with (IC_50_ = 2.54 ± 0.138, 3.40 ± 0.185, 5.82 ± 0.317, 6.24 ± 0.34 and 6.55 ± 0.357 μM, respectively). In the meantime, compounds **9a**, **9b**, **9e**, **9m**, and **9p**, with IC_50_ = 19.84 ± 1.08, 12.24 ± 0.67, 14.13 ± 0.77, 18.95 ± 1.03 and 10.81 ± 0.59 μM, respectively, exhibited moderate cytotoxicity, whereas, pyrazolines **9d**, **9i**, **9l**, and **9n** with IC_50_ ranging from 35.15 ± 1.916 to 58.01 ± 3.16 μM displayed the lowest cytotoxic activity.

Additionally, cytotoxicity evaluation against T-47D breast cancer cell line highlighted that compounds **9c** and **9o** displayed good anticancer actions with IC_50_ values of 8.45 ± 0.27 and 7.36 ± 0.30 μM, respectively. In turn, pyrazolines **9b**, **9e**, **9h**, and **9p** with (IC_50_ ranging from 11.42 ± 0.67 to 19.47 ± 0.92 μM) exhibited moderate cytotoxicity. Besides, compound **9f**, **9j**, **9m**, and **9n** with IC_50_ ranging from 27.1 ± 1.08 to 67.29 ± 2.51 μM demonstrated weak cytotoxic activity. Finally, pyrazolines **9a**, **9d**, **9i**, and **9l** appeared to be inactive.

Next, we inspected the influence of the substitution on the benzylidine moiety on the cytotoxic activities. Substitution of the benzylidine moiety at C-4 with hydrophilic or lipophilic electron donating groups (like the hydroxyl, methoxy and *N*,*N*-dimethylamino groups) resulted in an enhanced cytotoxic activities in comparison to the counterparts bearing unsubstituted benzylidine motif. In details, compounds **9b**, **9c** and **9e** bearing *para*-substituted benzylidine motifs showed better activity against both MDA-MB-231 (IC_50_ = 12.24 ± 0.67, 2.54 ± 0.138 and 14.13 ± 0.77 μM, respectively) and T-47D (IC_50_ = 18.61 ± 0.87, 8.45 ± 0.27 and 3.14 ± 0.11 μM, respectively) cell lines than the unsubstituted analogue **9a** (IC_50_ = 19.84 ± 1.08 and > 100 μM, toward MDA-MB-231 and T-47D cells respectively). Similarly, pyrazolines **9j**, **9k** and **9m** exerted more potent activity towards both MDA-MB-231 (IC_50_ = 6.24 ± 0.34, 1.14 ± 0.06 and 18.95 ± 1.03 μM, respectively) and T-47D (IC_50_ = 35.10 ± 2.04, 4.92 ± 0.28 and 27.63 ± 1.24, respectively) cell lines than the corresponding unsubstituted counterpart **9i** (IC_50_ = 24.68 ± 1.35 and > 100 μM, toward MDA-MB-231 and T-47D cells respectively).

It’s noteworthy mentioning that moving the methoxy group from *para* to *ortho* position led to a decrease of the cytotoxic influence against both the tested cancer cell lines; hybrids **9d** (IC_50_ = 45.02 ± 2.45 and > 100 μM) and **9l** (IC_50_ = 58.01 ± 3.16 and > 100 μM) *vs*. compounds **9e** (IC_50_ = 14.13 ± 0.77 and 12.37 ± 0.33 μM) and **9m** (IC_50_ = 18.95 ± 1.03 and 27.63 ± 1.24 μM). Moreover, it was found that the best di-substitution pattern (hydroxyl and methoxy substitution) for the benzylidene moiety is the 3-hydroxy-4-methoxy pattern for the 4-fluorophenyl bearing derivatives **9a-h** and the 4-chlorophenyl bearing derivatives **9i-p** against both the examined cell lines, except the 4-hydroxy-3-methoxy substitution in compound **9p** toward MDA-MB-231 cell line.

Furthermore, the cytotoxic impact for the most potent anti-proliferative thiazolyl pyrazolines reported in this work against MDA-MB-231 cell line (**9c**, **9f.**, **9g**, **9h**, **9j**, **9k** and **9p;** with IC_50_ value less than 10 µM) was evaluated toward the normal human MCF-10A cells (Table 1). The examined pyrazolines (**9c**, **9f.**, **9g**, **9h**, **9j**, **9k** and **9p)** exerted weak to non-significant activities against MCF-10A cell line (IC_50_ range: from 31.74 ± 3.10 to 83.62 ± 6.74), which highlights their safety and selectivity toward the cancer cells.

#### EGFR inhibitory activity

From the aforementioned data presented in Table 1 concerning the cytotoxicity, compounds **9g** and **9k** were identified as the most promising members among the series and showed superior cytotoxicity against MDA-MB-231 and T-47D cell lines. So, compounds **9g** and **9k** were further evaluated for their potential EGFR inhibitory action, using erlotinib as a reference EGFR inhibitor.

Table 2 shows the data as IC_50_ values. The results displayed that the two candidates exhibited EGFR inhibitory activity at the nanomolar level. In comparison to erlotinib (IC_50_ = 57 ± 3 nM), compounds **9g** and **9k** exhibited good activity as EGFR inhibitors in nanomolar concentrations (IC_50_ = 267 ± 12 and 395 ± 17 nM, respectively).

**Table 2:** In vitro inhibitory activity of hybrids **9g** and **9k** towards EGFR.


Compound	IC_50_ (nM)
	EGFR
**9g**	267 ± 12
**9k**	395 ± 17
**Erlotinib**	57 ± 3

#### Cell cycle analysis

In this work, MDA-MB-231 cells were treated with the most cytotoxic members (compounds **9g** and **9k**) at a concentration of 0.62 and 1.14 µM, respectively (corresponding to their IC_50_ values against MDA-MB-23 cells). Investigating the results, it can be noticed that compounds **9g** and **9k** induced a significant increase in the cell population at Sub-G1 phase (31.05% and 27.86%, respectively), comparing to control cells (2.13%). For the S phase, the percentage of MDA-MB-23 cells was increased from 32.11% in control cells to 35.43% for compound **9g** while the % was decreased to 28.45% for compound **9k**.

**Figure 2:**
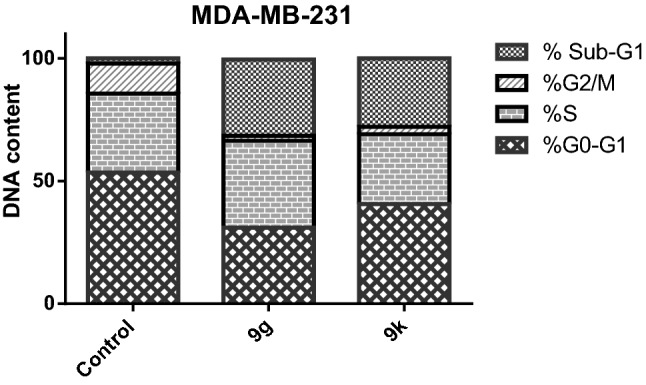
Effect of **9g** and **9k** on the phases of cell cycle of MDA-MB-231 cells.

Moreover, compounds **9g** and **9k** exhibited insignificant decrease in the cell population (31.0217% and 40.61%, respectively) at G0-G1 phase, comparing to the control cells (53.45%). Additionally, a marked decrease in the cell population for compounds **9g** (2%) and **9k** (3.08%) was observed at the G2/M phase, comparing to control cells (12.31%). These findings verify that the cytotoxicity of compounds **9g** and **9k** against MDA-MB-231cells was due to arresting the cell growth at G2/M phase (Fig. 2, Table 3).

**Table 3:** Influence of pyrazole-thiazol-4-one hybrids **9g** and **9k** on the cell cycle phases of MDA-MB-231 cells.

Com	%G0-G1	%S	%G2/M	%Sub-G1
9g	31.0217	35.43	2	31.05
9k	40.61	28.45	3.08	27.86
Control	53.45	32.11	12.31	2.13

#### Apoptosis assay

The most potent anticancer agents in the current work **9g** and **9k** were chosen for the assessment of apoptosis in MDA-MB-231cell line using Annexin V/propidium iodide (PI) double staining assay method. In this method, MDA-MB-231 cells were incubated with compounds **9g** and **9k** at the IC_50_ concentrations (0.62 and 1.14 µM, respectively) for 48 h.

**Figure 3:**
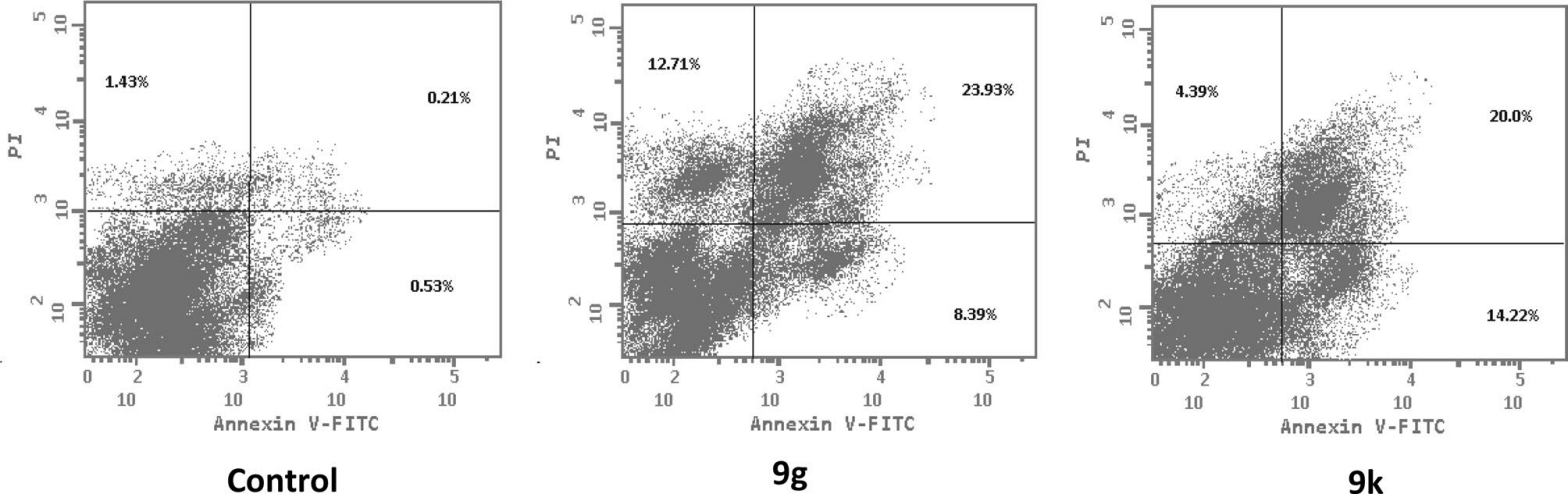
Effect of pyrazolines **9g** and **9k** on the percentage of annexin V-FITC-positive staining in MDA-MB-231 cells.

The results revealed that compounds **9g** and **9k** triggered more apoptotic cells in comparison to control cells. In particular, pyrazoline **9g** induced apoptosis by 32.32% (early apoptosis = 8.39% & late apoptosis = 23.93%) while pyrazoline **9k** induced apoptosis by 34.22% (early apoptosis = 14.22% & late apoptosis = 20%) compared to 0.94% in the control cells (0.73% and 0.21% for early and late apoptosis, respectively). From these findings, we concluded that compounds **9g** and **9k** could induce apoptosis in MDA-MB-231 cells (Fig. 3 and Table 4).

**Table 4:** Distribution of apoptotic cells within MDA-MB-231 cell line upon treatment with **9g** and **9k**, using AnnexinV-FITC/PI assay.

Com	Early apoptosis (lower right %)	Late apoptosis (upper right %)	Total (L.R % + U.R %)
9g	8.39	23.93	32.32
9k	14.22	20	34.22
Control	0.73	0.21	0.94

### Modeling studies

#### Docking study

Elucidating the potential binding mode between new compounds and their target became inevitable in lead discovery studies to provide futuristic insights for further optimization. Accordingly, both compounds **9k** and **9g** were docked into the predetermined active site of EGFR using MOE 2019.02.

**Figure 4:**
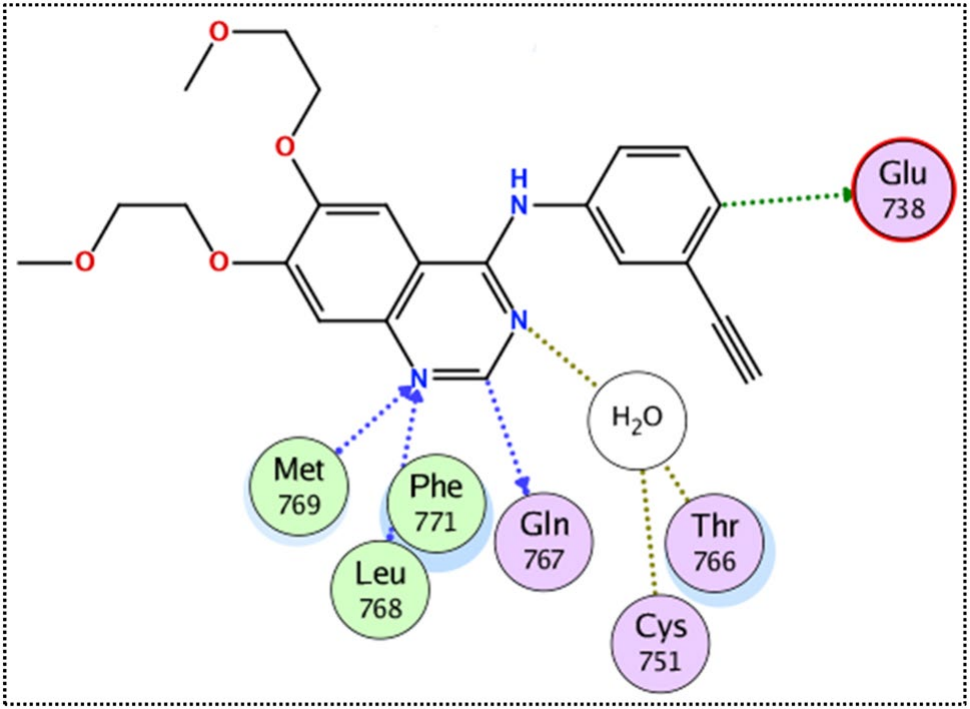
The binding mode of the crystal reference erlotinib with the EGFR receptor.

Exploring the binding mode of the crystal reference erlotinib with the EGFR has revealed the essential hydrogen bond interactions with the key residues Met 769 and Leu 768. Besides, the phenyl and quinazoline rings of erlotinib participated in carbon hydrogen bond interactions with Glu 738 and Gln 767, respectively. The importance of solvent molecules in EGFR active site was unequivocal, acting as a bridge for two interactions between the nitrogen of quinazoline ring with Cys 751 and Thr 766, Fig. 4.

**Figure 5:**
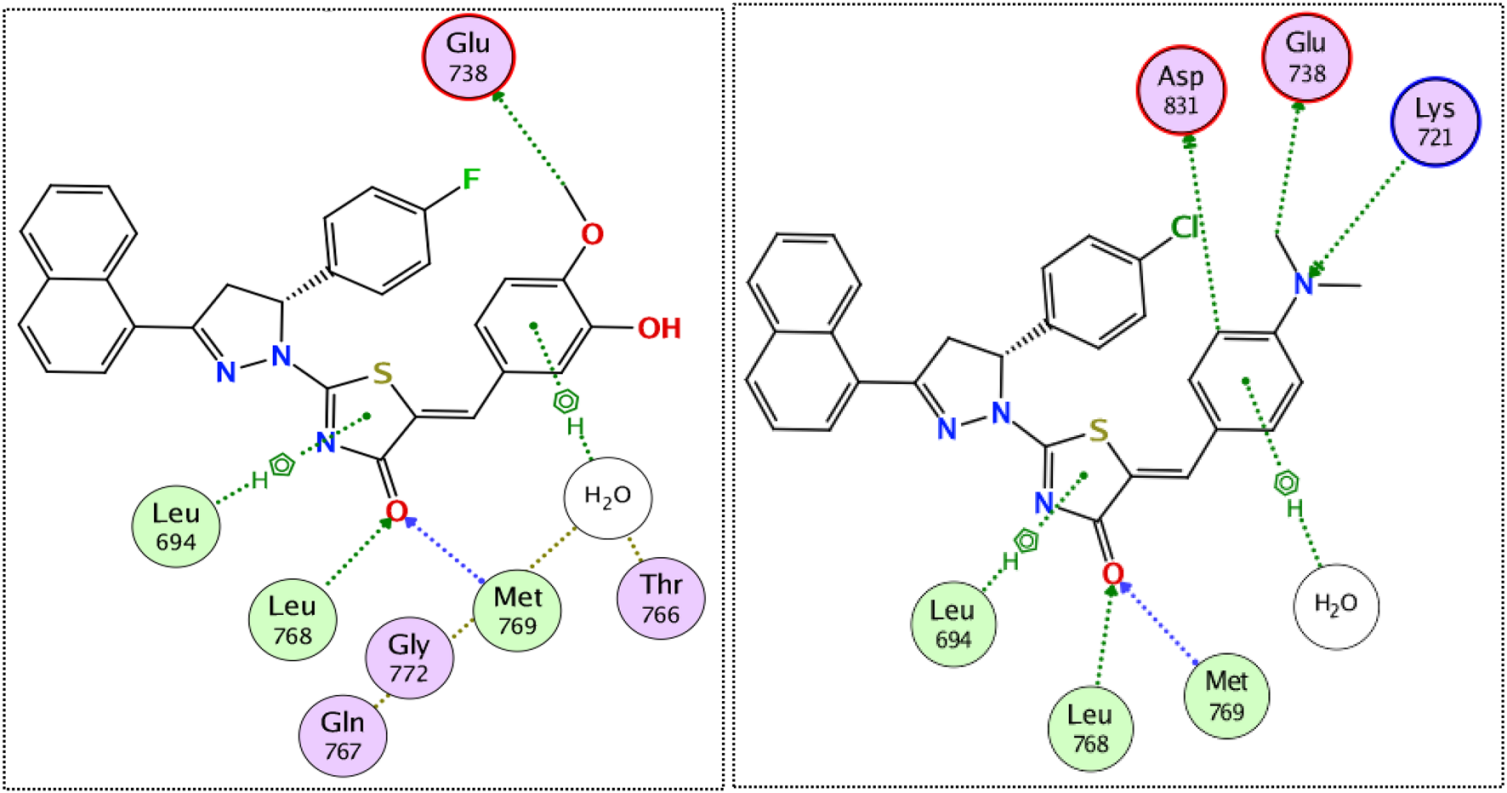
The 2D interaction diagram of EGFR receptor with **9g** (left) **9k** (right).

Interestingly, the docking of both compounds **9k** and **9g** induced a favorable binding upon interacting with EGFR through a binding pattern perfectly aligned with that of erlotinib. The synthesized compounds **9g** and **9k** achieved docking scores of −11.8 and −11.4 kcal/mol respectively, that nearly match the docking score of erlotinib (− 12.0 kcal/mol). As depicted from Fig. 5, the carbonyl group of the thiazol-4(5*H*)-one ring in compound **9g** acted as a hydrogen bond acceptor for 2 H-bonds with the key residues Met 769 and Leu 768, in addition the *para* methoxy group of compound **9g** was engaged in a carbon hydrogen bond interaction with Glu738. Once again, the water molecules played a crucial role in linking compound **9g** with Thr766 and Gln767.

**Figure 6:**
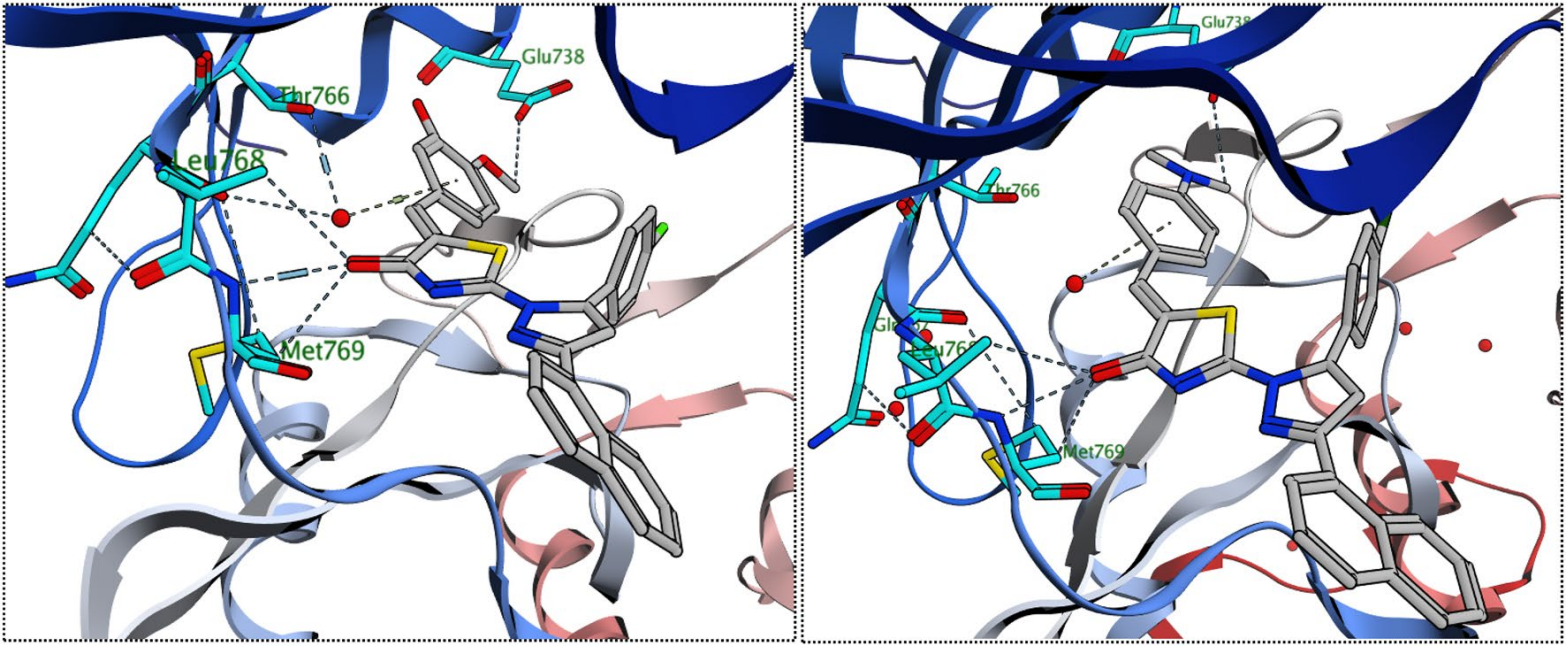
The 3D interaction diagram of EGFR receptor with **9g** (left) **9k** (right).

In a similar manner, compound **9k** engaged in two hydrogen bonds via its carbonyl group with the essential residues Met 769 and Leu 768. The *N*,*N*-dimethyl aniline ring of compound **9k** involved in one hydrogen bond with Lys 721 and three carbon hydrogen bonds with Glu738, Asp831 and a water molecule. Unlike compound **9g**, the water effect was less significant in case of compound **9k**, which explains the superiority of compound **9g** in docking score and biological activity. Noteworthy, the difference in the kinase activity of compounds **9g** and **9k** could be attributed to the grafting of different substituents (*para*-fluoro in **9g** and *para*-chloro in **9k**) to 4,5-dihydro-1*H*-pyrazolyl ring. The large size of the chlorine atom hinders the ability of compound **9k** to get closer enough with the solvent molecules unlike the smaller fluorine atom that allows compound **9g** to get closer enough to the water molecule attached to the Thr766 and Gln767, Fig. 5–6.

#### Molecular dynamics

##### RMSD and RMSF analysis

In the current study, further in silico investigations were achieved through molecular dynamic simulations. Molecular dynamics (MD) simulation provides many valuable information and parameters to study the dynamicity of biological systems. Amongst these information, MD could provide insights into precise estimation of the binding strength of a docked complex of a ligand and a target. Accordingly, the predicted binding co-ordinates retrieved from the docking of EGFR with **9g**, **9k** and erlotinib were moved forward to MD simulation. To provide a comparative mean for the effect of each ligand on the stability of the EGFR enzyme, the later was subjected to MDS using the Apo form.

**Figure 7:**
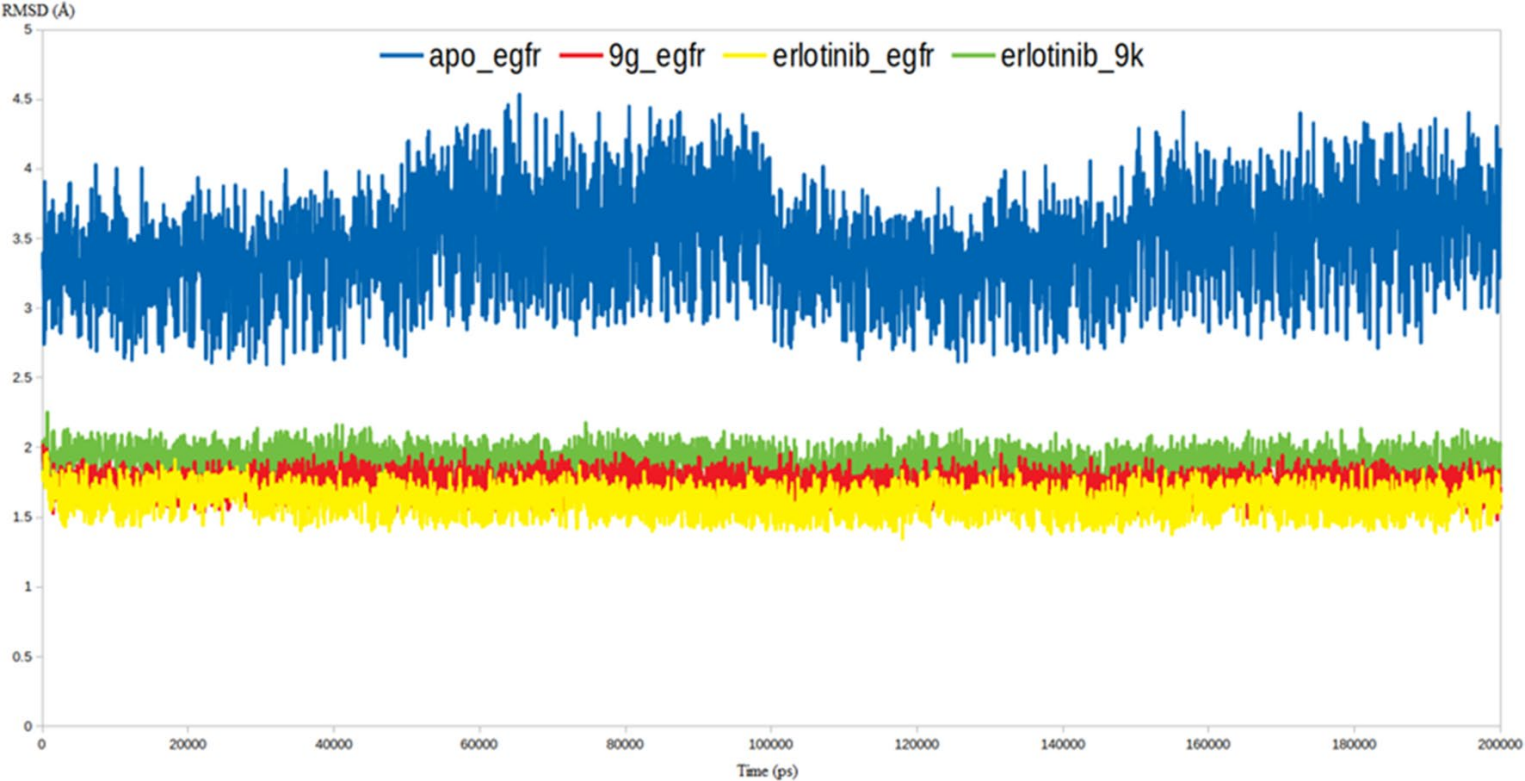
RMSD analysis for the MD simulations.

As demonstrated by Fig. 7, all the three inhibitors were able to stabilize the EGFR enzyme as indicated by their lower RMSD values comparing to the RMSD value of Apo EGFR. The EGFR-**9g** and EGFR-**9k** complexes had RMSD values of 1.8 and 2.1 Å, respectively, that are very comparable to that of EGFR-erlotinib (1.75 Å), while the RMSD of the Apo EGFR reached 4.5 Å. In cancer cells, EGFR serves as the switch-on for the intracellular signaling pathways to trigger cell division and uncontrolled growth. In this respect, the high dynamicity seen in the Apo EGFR as discerned from the high RMSD values is perfectly aligned with its intended oncogenic function. The capability of compounds **9g** and **9k** to restrict the dynamic nature of the EGFR via the formation of stable complexes as indicated by the lower RMSD values is a valid indicator for their inhibitory impact on EGFR (Fig. 7).

**Figure 8:**
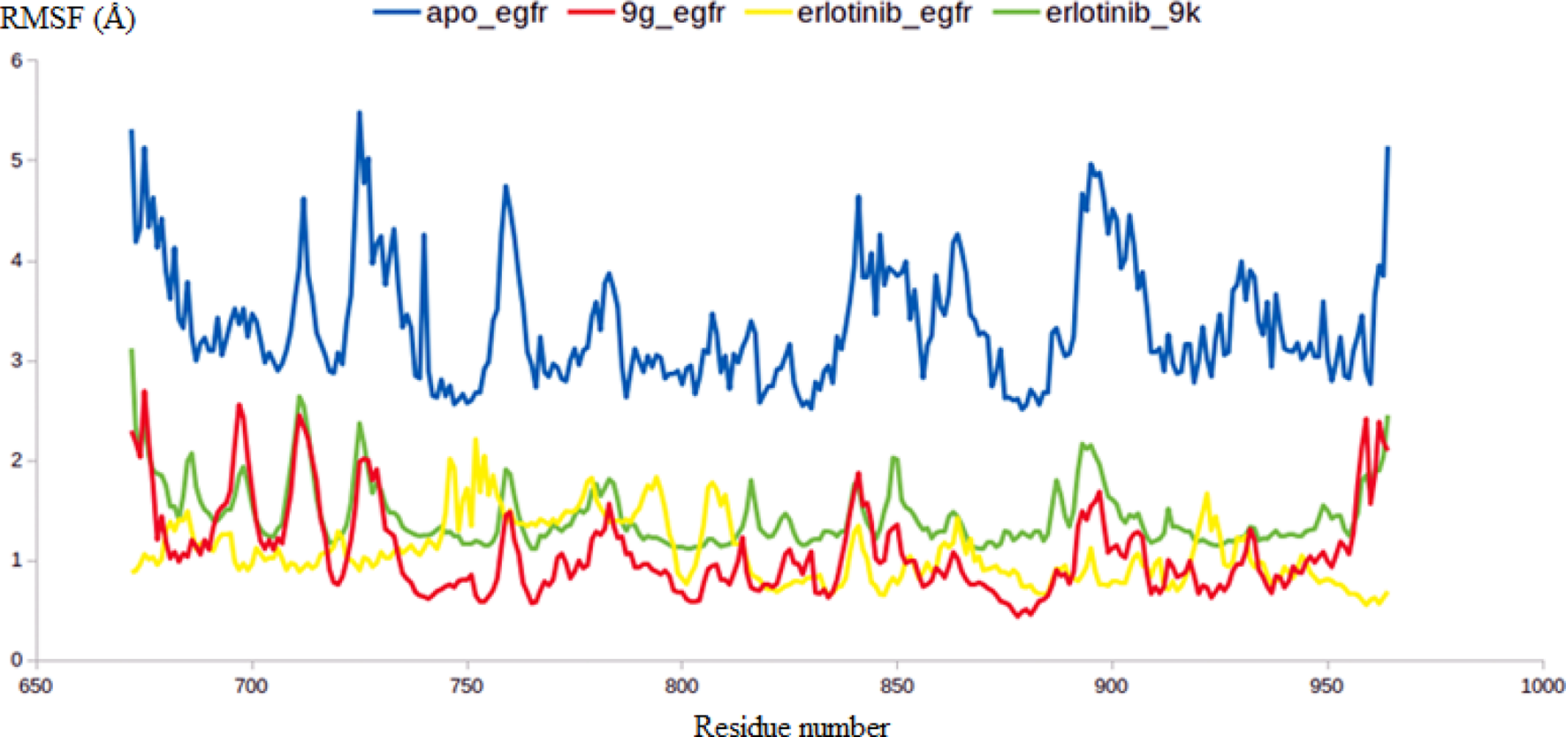
The RMSF analysis for the MD simulations.

To further endorse the outputs of the RMSD calculations, the RMSF values for all the residues in the four systems have been computed. As expected the RMSF values lead to the same conclusion retrieved from the RMSD calculations in which the average RMSF of the Apo EGFR residues reached 4.2 Å, while the average RMSF values for the EGFR residues in complex with **9g**, **9k** and erlotinib reached an average 1.8, 2.1, 1.7 Å respectively (Fig. 8). To this end, both RMSD and RMSF values validated the proposed binding mode between **9g** and **9k** with the EGFR active site and attributed the inhibitory activity of both molecules to their ability to form a stable complex with the EGFR.

##### Binding free energy calculations using MM-PBSA approach

Attempting to further evaluate the strength of binding between the EGFR enzyme and the newly prepared compound **9g** and **9k**, the g_mmpbsa package generated by Kumari et al. was brought in action to calculate the binding free energies between the EGFR enzyme and the proposed molecules **9g** and **9k**. The generated trajectories from the production stage were used to calculate all the forms of binding free energy. These energy types include Electrostatic energy, van der Waal energy, Polar solvation energy and SASA energy. All the previous types of energy were calculated to the three complexes containing EGFR bound to either **9g**, **9k** or erlotinib (Table 5).

**Table 5:** The binding free energies of **9g**, **9k** and erlotinib in complex with EGFR.

Complex	ΔE_binding_ (kJ/mol)	ΔE_Electrostatic_ (kJ/mol)	ΔE_polar solvation_ (kJ/mol)	ΔE_*Vander Waal*_ (kJ/mol)	SASA (kJ/mol)
9g	− 288.1 ± 4.9	− 123.3 ± 5.2	87.1 ± 1.2	− 224.3 ± 7.5	− 27.6 ± 0.2
9k	− 251.9 ± 3.8	− 105.7 ± 3.1	72.1 ± 1.7	− 196.1 ± 6.1	− 22.2 ± 0.2
Erlotinib	− 286.8 ± 4.4	− 118.3 ± 3.8	88.5 ± 2.4	− 230.9 ± 6.4	− 26.1 ± 0.4

Interestingly, the calculated binding free energy for the three small molecules were comparable to each other in which compound **9g** and **9k** achieved binding free energies of − 288.1 ± 4.9 and − 251.9 ± 3.8 (kJ/mol) respectively, whereas the crystal reference erlotinib achieved − 286.8 ± 4.4 (kJ/mol). These results augmented all the in silico calculations giving credit to the predicted binding mode of both **9g** and **9k** within EGFR. Moreover, all the MD and energy calculations favored compound **9g** over compound **9k** as consistent with the early results of enzyme assay.

**Figure 9:**
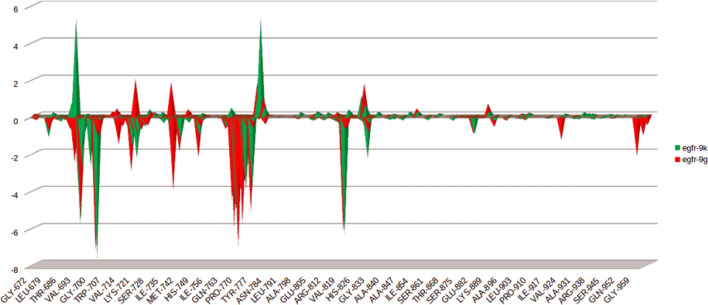
The per residue contribution for the binding free energy of **9g** and **9k** with EGFR.

In terms of binding energy the energy contribution for each residue was calculated to provide deep insights into the effect of compounds **9g** and **9k** upon engaging in the binding pocket of EGFR. This contribution was calculated through decomposing the total binding free energy of each complex into per residue contribution energy. As demonstrated by Fig. 9, the binding of compound **9g** to the EGFR active site resulted in favor energy contribution in all the Key residues especially those of close contact to compound **9g** such as Met769 and Leu768 Glu738 Thr766 and Gln767. In a similar fashion, the binding of compound **9k** contributed favorably to the free energy of the surrounding residues significantly to Met769, Leu768 and Glu738 and in lesser extent to Thr766 and Gln767. Consistent with the results of docking, MD and MMPBSA analyses, the per residue decomposition results supported the predicted binding mode of compounds **9g** and **9k** with EGFR kinase.

## Conclusions

In the current work, new series of pyrazole-thiazol-4(5*H*)-one hybrids (**9a-p**) were synthesized as potential anti-breast cancer molecules. The anti-proliferative activities for the synthesized molecules (**9a-p**) were evaluated against MDA-MB-231 and T-47D BC cell lines. The obtained results showed that MDA-MB-231 cell line was more sensitive to the influence of the tested members than T-47D. Also, it was noticed that the 4-fluorophenyl derivative **9g (**IC_50_ = 0.62 ± 0.03 against MDA-MB-231 and 3.14 ± 0.11 µM against T-47D**)** and the 4-chlorophenyl derivative **9k** (IC_50_ = 1.14 ± 0.06 against MDA-MB-231 and 4.92 ± 0.28 µM against T-47D) were the most potent cytotoxic members against the two BC cell lines, comparing to the positive control, doxorubicin (IC_50_ = 3.67 ± 0.2 and 2.73 ± 0.09 µM against MDA-MB-231 and T-47D, respectively). Accordingly, both compounds **9g** and **9k** were tested for their activity against EGFR, where their achieved nanomolar inhibitory activity; IC_50_ = 267 ± 12 and 395 ± 17 nM for **9g** and **9k**, respectively. Further investigations unveiled their ability to arrest cell cycle at G2/M phase in addition to apoptosis induction in the MDA-MB-231 cell line as evidenced by Annexin V analysis. Interestingly, the docking of both compounds **9g** and **9k** induced a favorable binding upon interacting with EGFR through a binding pattern perfectly aligned with the crystal reference erlotinib. Compound **9g** achieved docking score (− 11.8 kcal/mol) favorable than **9k (**− 11.4 kcal/mol) and both scores are close to the docking score of erlotinib (− 12.0 kcal/mol). The docking results were further endorsed by 200 ns of molecular dynamic simulations alongside with MM-PBSA calculations and both revealed that compound **9g** had more stable complex with EGFR than **9k** with RMSD of 1.8, Å and binding free energy of − 288.1 ± 4.9 kJ/mol. The results of per residue decomposition supported predicted binding mode of compounds **9g** and **9k** with EGFR enzyme and showed favorable energy contribution of the key residues in the active site especially upon the binding compound **9g**. The overall molecular modeling results attributed the superiority of compound **9g** to the smaller size of the fluorine substituent that enables perfect fitting of compound **9g** in EGFR active pocket.

## Experimental

### Chemistry

#### General

The IR spectra were recorded on Schimadzu FT-IR 8400S spectrophotometer. The NMR spectra were recorded via Bruker spectrometer at 400 MHz. ^13^C NMR spectra were run at 100 MHz in DMSO-*d6*. Chemical shifts (*δ*_*H*_) were reported relative to the solvent (DMSO-*d*_*6*_). Elemental analyses were performed by FLASH 2000 CHNS/O analyzer, Thermo Scientific. Chalcones **3a-b** were reported previously^29,30^.

#### Synthesis of 3-(naphthalen-1-yl)-4,5-dihydropyrazole-1-carbothioamide derivatives 5a-b

To hot stirred suspension of 1-(naphthalen-1-yl)propen-1-one derivatives **3a-b** (4.5 mmol) in 30 mL of abs. EtOH with sodium hydroxide (0.55 g, 13.5 mmol), equivalent amount of thiosemicarbazide **4** (0.40 g, 4.5 mmol) was added. The mixture was heated under reflux for 2 h with TLC monitoring. After full consumption of all starting, the produced precipitate was collected, washed with hot water (4 × 5 mL) and recrystallized form acetonitrile to furnish the intermediates 3-(naphthalen-1-yl)-4,5-dihydro-1*H*-pyrazole-1-carbothioamides **5a-b**, which utilized in the next reaction without more purification.

#### Synthesis of 3-(naphthalen-1-yl)-4,5-dihydropyrazol-1-yl)thiazol-4(5*H*)-one derivatives 7a-b

The previously prepared thioamide intermediates **5a-b** (2 mmol) and sod. acetate (0.34 g, 4 mmol) were added to a solution of ethyl bromoacetate **6** (0.33 g, 2 mmol) in absolute ethanol (12 mL). The reaction mixture was refluxed for 4 h with TLC monitoring. After completion of the reaction, it was cooled to r.t. The formed solid was filtrated, washed with hexane (3 × 4 mL) and recrystallized from glacial acetic acid to produce the key intermediates 3-(naphthalen-1-yl)-4,5-dihydro-1*H*-pyrazol-1-yl)thiazol-4(5*H*)-one derivatives **7a-b**, which exploited in the next step without further purification.

#### Synthesis of target molecules 9a-p

To hot stirred solution of the appropriate aldehydes **8a-h** (0.5 mmol) in glacial AcOH (15 mL) with two equivalent amount of fused sod. acetate (0.08 g, 1 mmol) at round flask, an equivalent amount of the previously prepared key intermediates **7a-b** (1 mmol) was added. The reaction solution was left under reflux for 6 h, the formed solid was collected while hot, washed with MeOH, and recrystallized from DMF to get the targeted **9a-p**.

The spectral (IR and NMR) and elemental analysis for all the newly prepared pyrazolines **9a-p** were provided in the Supporting Materials.

### Biological evaluation

All the biological assays conducted in this study were performed as reported earlier; MTT cytotoxicity^31,32^ cell cycle^33^, Annexin V-FITC Apoptosis^34–36^ and EGFR kinase^37^ assays, (Supplementary Materials).

### Molecular modeling studies

#### Docking study

In this work, all the docking studies were conducted using Molecular operating environment (MOE 2019.02) Software^38,39^. The X-ray crystal structures of EGFR in complex with erlotinib were downloaded from the protein databank PDB IDs 1M17. At the beginning, the hydrogens and charges of the receptors were optimized using AMBER10: EHT embedded in MOE software. The binding site of the EGFR enzyme was constructed where the co-crystalized erlotinib is bound. As a part of docking validation, the x-ray coordinates of the co-crystalized ligand was retrieved through re-docking into the pre-determined active site. The previous step resulted in RMSD value of 0.77 Å between the co-crystalized pose and the docking pose indicating a valid docking protocol. After that, compounds **9g** and **9k** were docked into the EGFR binding domain using triangular matcher and London dg as a placement and scoring methods, respectively. At last, 2D and 3D interaction diagrams were generated by MOE to analyze the socking results.

#### Molecular dynamics

In this work, 4 molecular dynamic simulations (MDS) were conducted for 200 ns using GROMACS 2.1.1 software^40^. The free EGFR enzyme and the retrieved docking coordinates of the same enzyme bound Erlotinib, **9k** and **9g** were used as input structures for the molecular dynamics. The typical work scheme of Gromacs simulation was applied to conduct the four MDS^41–45^ (Supplementary Materials).

#### MM-PBSA calculation and per residue contribution

The MM-PBSA package of Kumari et al.^46^ was contrived to calculate the binding free energy between the ligands and the EGFR enzyme using the following equation.

$$\Delta {\text{G}}_{{({\text{Binding}})}} = {\text{ G}}_{{({\text{Complex}})}} - {\text{ G}}_{{({\text{Receptor}})}} - {\text{ G}}_{{\left( {{\text{Ligand}}} \right) }}$$ (Supplementary Materials)

Both the complexes of EGFR-9g and EGFR-9k complexes were subjected to such calculations and compared to the binding energy values of EGFR-Erlotinib.

To evaluate the contribution of each residue to the binding of **9g** and **9k**, the total free energy of each complex was decomposed per residues.

## Supplementary Information

Supplementary Information.

## Data Availability

All data generated or analyzed during this study are included in this published article [and its supplementary information files].
