# Supplementary material for: Design, synthesis, in vitro biological assessment and molecular modeling insights for novel 3-(naphthalen-1-yl)-4,5-dihydropyrazoles as anticancer agents with potential EGFR inhibitory activity
Source: Sci Rep. 2022 Jul 27;12:12821. doi: 10.1038/s41598-022-15050-8 (PMC9329325; doi:10.1038/s41598-022-15050-8)
Supplement: Supplementary file 1 — Supplementary Information. [file 41598_2022_15050_MOESM1_ESM.pdf]

Supporting Information

Design, synthesis, *in vitro* biological assessment and molecular modeling insights for novel 3-(naphthalen-1-yl)-4,5-dihydropyrazoles as anticancer agents with potential EGFR inhibitory activity

Wagdy M. Eldehna*, Mahmoud A. El Hassab, Zainab M. Elsayed, Tarfah Al-Warhi, Hazem Elkady, Mahmoud F. Abo-Ashour, Mohammed A.S. Abourehab, Ibrahim H. Eissa, Hatem A. Abdel-Aziz

Tables of Contents

1. Anti-proliferative Activity 2

2. Cell Cycle Analysis 2

3. AnnexinV-FITC/PI Apoptosis Assay 3

4. EGFR Kinase Inhibitory Activity 3

5. Characterisation of the target compounds (**9a-p**) 4-10

6. In silico Analysis 11

7. NMR Spectra 12-43

1. Anti-proliferative activity

The two examined breast cancer cell lines (MDA-MB-231 and T-47D) as well as the normal breast MCF-10A cells have been obtained from American Type Culture Collection (ATCC). Cells lines were maintained as monolayers in Dulbecco's Modified Eagle's Medium (DMEM) supplemented with 10% FBS, 2 mM L-glutamine, 100 U/ml penicillin and 100μg/ml streptomycin sulfate. Cells were sub-cultured with trypsine /EDTA solution, counted with haemocytometer and plated onto 96-well plates (5000 cells/well) and left overnight to form a semi-confluent monolayer. Cell monolayers were treated in quadrates with vehicle (DMSO, 0.1% v/v), test samples (thiazolyl-pyrazolines **9a-p**) or Doxorubicin as positive control for an exposure time of 48 h. At the end of exposure, MTT solution in PBS (5 mg/ml) was then added to all well including no cell blank and left to incubate for 90 min. The formation of formazan crystals were visually confirmed using phase contract microscopy. DMSO (100 µl/well) was added to dissolve the formazan crystals with shaking for 10 min after which the absorbance was read at 590 nm against no cell blanks on a FLuo Star Optima microplate reader (BMG technologies, Germany). Cell proliferation was calculated comparing the OD values of the DMSO control wells and those of the samples represented as % proliferation to the control. Dose-response experiment was performed on samples producing > or =50% loss of cell proliferation using five serial 2-fold dilutions (50, 25, 12.5, 6.25 and 3.125 µM) of the sample. IC_{50} values (concentration of sample causing 50% loss of cell proliferation of the vehicle control) were calculated using non-linear regression curve fitting of the dose response plots on GraphPad Prism V.6.0 software.

2. Cell Cycle Analysis

Breast cancer MDA-MB-231 cells were treated with compounds **9g and 9k** for 24 h (at their IC_{50} concentration), and then cells were washed twice with ice-cold phosphate buffered saline (PBS). Subsequently, the treated cells were collected by centrifugation, fixed in ice-cold 70% (*v*/*v*) ethanol, washed with PBS, re-suspended with 100 µg/mL RNase, stained with 40 µg/mL PI, and analyzed by flow cytometry using FACS Calibur (Becton Dickinson, BD, Franklin Lakes, NJ, USA). The cell cycle distributions were calculated using CellQuest software 5.1 (Becton Dickinson).

3. Annexin V-FITC Apoptosis Assay

Phosphatidylserine externalization was assayed using Annexin V-FITC/PI apoptosis detection kit (BD Biosciences, USA) according to the manufacturer's instructions. Breast cancer MDA-MB-231 cells were cultured to a monolayer then treated with compounds **9g and 9k** at their IC_{50} concentration. Briefly, cells were then harvested *via* trypsinization, and rinsed twice in PBS followed by binding buffer. Moreover, cells were re-suspended in 100 μL of binding buffer with the addition of 1 μL of FITC-Annexin V followed by an incubation period of 30 min at 4 °C. Cells were then rinsed in binding buffer and resuspended in 150 μL of binding buffer with the addition of 1 μL of DAPI (1 μg/μL in PBS). Cells were then analyzed using the flow cytometer BD FACS Canto II and the results were interpreted with FlowJo7.6.4 software (Tree Star, Ashland, OR, USA).

4. EGFR Kinase Inhibitory Activity

Compounds **9g and 9k** were tested in vitro for inhibition of EGFR tyrosine kinase using ADP-Glo™ Kinase Assay (Promega, Catalogue No. V3831) which is a luminescent kinase assay that measures ADP formed from a kinase reaction. ADP is converted into ATP which is converted into light by Ultra-Glo™ Luciferase. The luminescent signal positively correlates with ADP amount and kinase activity.

Protocol: first dilute enzyme, substrate, ATP and inhibitors in Tyrosine Kinase Buffer (40 mM Tris,7.5; 20 mM $MgCl_2$; 0.1 mg/ml BSA (bovine serum albumin); 2 mM $MnCl_2$; 50μM DTT), then add to the wells of 384 low volume plate: 1 μl of inhibitor or (5% DMSO), 2 μl of enzyme and 2 μl of substrate/ATP mix. Incubate at room temperature for 60 minutes, add 5 μl of ADP-Glo™ Reagent, incubate at room temperature for 40 minutes, add 10 μl of Kinase Detection reagent, incubate at room temperature for 30 minutes and finally record luminescence (Integration time 0.5-1second).

5. Characterisation of the target compounds (9a-p)

5-Benzylidene-2-(5-(4-fluorophenyl)-3-(naphthalen-1-yl)-4,5-dihydro-1H-pyrazol-1-yl)thiazol-4(5H)-one ***9a***.

White crystals (yield 68%), m.p. 201-203 °C; IR (KBr, ν cm^{-1}) 1702 (C=O); ^1H NMR δ *ppm*: 3.67 (dd, 1H, CH_2a of pyrazoline ring, *J*= 4.4 Hz, 18.0 Hz), 4.39 (dd, 1H, CH_2b of pyrazoline ring, *J*= 11.2 Hz, 18.0 Hz), 5.92 (dd, 1H, CH of pyrazoline ring, *J*= 4.0 Hz, 11.2 Hz), 7.22-7.27(m, 2H, Ar-H), 7.41-7.48 (m, 3H, Ar-H), 7.53-7.57 (m, 2H, Ar-H), 7.61-7.63 (d, 1H, Ar-H, *J*= 7.6 Hz), 7.65- 7.70 (m, 4H, Ar-H), 7.80 (t, 1H, Ar-H, *J*= 8.0 Hz), 7.94 (d, 1H, Ar-H, *J*= 7.6 Hz), 8.07 (d, 1H, Ar-H, *J*= 8.4 Hz), 7.14 (d, 1H, Ar-H, *J*= 8.0 Hz), 9.16 (d, 1H, Ar-H, *J*= 8.0 Hz); ^{13}C NMR δ *ppm*: 46.60 (CH_2), 62.49 (CH), 116.19, 116.41, 125.74, 126.48, 127.05, 128.44, 128.72, 128.80, 128.87, 129.55, 129.80, 130.19, 130.30, 130.45, 130.89, 131.72, 132.93, 134.11, 134.34, 136.74, 162.34, 171.21, 179.62; Anal. Calcd. for $C_{29}H_{20}FN_3OS$: C, 72.94; H, 4.22; N, 8.80; found C, 72.74; H, 4.26; N, 8.86.

2-(5-(4-Fluorophenyl)-3-(naphthalen-1-yl)-4,5-dihydro-1H-pyrazol-1-yl)-5-(4-hydroxybenzylidene)thiazol-4(5H)-one ***9b***.

White crystals (yield 72%), m.p. 224-226 °C; IR (KBr, ν cm^{-1}) 3210 (OH) and 1700 (C=O); ^1H NMR δ *ppm*: 3.63 (dd, 1H, CH_2a of pyrazoline ring, *J*= 4.0 Hz, 17.6 Hz), 4.37 (dd, 1H, CH_2b of pyrazoline ring, *J*= 11.6 Hz, 18.4 Hz), 5.89 (dd, 1H, CH of pyrazoline ring, *J*= 4.4 Hz, 11.2 Hz), 6.70-6.74 (m, 2H, Ar-H), 7.22-7.26 (m, 2H, Ar-H), 7.37-7.44 (m, 4H, Ar-H), 7.56 (s, 1H, Ar-H), 7.61-7.69 (m, 2H, Ar-H), 7.81 (t, 1H, Ar-H, *J*= 8.0 Hz), 7.92 (d, 1H, Ar-H, *J*= 8.0 Hz), 8.07 (d, 1H, Ar-H, *J*= 8.0 Hz), 8.13 (d, 1H, Ar-H, *J*= 8.0 Hz), 9.19 (d, 1H, Ar-H, *J*= 8.0 Hz); ^{13}C NMR δ *ppm*: 46.48 (CH_2), 62.24 (CH), 116.14, 116.35, 118.51, 125.73, 126.52, 126.60, 127.04, 127.92, 128.58, 128.66, 128.83, 129.13, 129.49, 130.30, 130.60, 132.65, 132.95, 133.07, 134.10, 137.11, 160.93, 163.35, 170.88; Anal. Calcd. for $C_{29}H_{20}FN_3O_2S$ C, 70.57; H, 4.08; N, 8.51; found C, 70.31; H, 4.05; N, 8.45.

5-(4-(Dimethylamino)benzylidene)-2-(5-(4-fluorophenyl)-3-(naphthalen-1-yl)-4,5-dihydro-1H-pyrazol-1-yl)thiazol-4(5H)-one ***9c***.

White crystals (yield 70%), m.p. 217-219 °C; IR (KBr, ν cm^{-1}) 1710 (C=O); ^1H NMR δ *ppm*: 3.02 (s, 6H, $N(CH_3)_2$), 3.65 (dd, 1H, CH_2a of pyrazoline ring, *J*= 4.0 Hz, 17.6 Hz), 4.38 (dd, 1H,

CH_2b of pyrazoline ring, *J*= 11.6 Hz, 18.4 Hz), 5.90 (dd, 1H, CH of pyrazoline ring, *J*= 4.4 Hz, 11.2 Hz), 6.48 (d, 2H, Ar-H, *J*= 8.0 Hz), 7.22-7.26 (m, 2H, Ar-H), 7.41-7.47 (m, 2H, Ar-H), 7.50 (d, 2H, Ar-H, *J*= 8.4 Hz), 7.59-7.71 (m, 4H, Ar-H), 7.83 (t, 1H, Ar-H, *J*= 8.4 Hz), 7.94 (d, 1H, Ar-H, *J*= 7.6 Hz), 8.09 (d, 1H, Ar-H, *J*= 8.4 Hz), 8.14 (d, 1H, Ar-H, *J*= 8.0 Hz), 9.21 (d, 1H, Ar-H, *J*= 8.8 Hz); ^{13}C NMR δ *ppm*: 40.10 ($N(CH_3)_2$), 46.50 (CH_2), 62.27 (CH), 112.63, 116.15, 116.37, 121.12, 121.41, 125.76, 126.54, 127.00, 128.63, 128.72, 128.83, 129.56, 130.32, 130.75, 132.15, 132.75, 132.91, 134.13, 161.27, 170.86; Anal. Calcd. for $C_{31}H_{25}FN_4OS$: C, 71.52; H, 4.84; N, 10.76; found C, 71.77; H, 4.80; N, 10.69.

2-(5-(4-Fluorophenyl)-3-(naphthalen-1-yl)-4,5-dihydro-1H-pyrazol-1-yl)-5-(2-methoxybenzylidene)thiazol-4(5H)-one ***9d***.

White crystals (yield 78%), m.p. 212-213 °C; IR (KBr, ν cm^{-1}) 1709 (C=O); ^{1}H NMR δ *ppm*: 3.35 (s, 3H, OCH_3), 3.63 (dd, 1H, CH_2a of pyrazoline ring, *J*= 4.4 Hz, 18.0 Hz), 4.36 (dd, 1H, CH_2b of pyrazoline ring, *J*= 11.2 Hz, 18.4 Hz), 5.89 (dd, 1H, CH of pyrazoline ring, *J*= 4.0 Hz, 11.2 Hz), 7.10-7.13 (m, 2H, Ar-H), 7.21-7.27 (m, 2H, Ar-H), 7.40-7.48 (m, 3H, Ar-H), 7.55-7.58 (m, 1H, Ar-H), 7.60-7.68 (m, 2H, Ar-H), 7.76-7.80 (m, 1H, Ar-H), 7.91-7.95 (m, 2H, Ar-H), 8.06 (d, 1H, Ar-H, *J*= 8.0 Hz), 8.13 (d, 1H, Ar-H, *J*= 8.0 Hz), 9.16 (d, 1H, Ar-H, *J*= 8.8 Hz); ^{13}C NMR δ *ppm*: 46.55 (CH_2), 56.16 (OCH_3), 62.39 (CH), 116.38, 121.53, 122.94, 125.75, 126.45, 127.05, 128.68, 128.76, 129.17, 129.55, 130.31, 130.85, 132.37, 132.89, 133.16, 134.11, 136.79, 137.21, 141.54, 146.06, 158.37, 162.08, 171.44, 179.68 (C=O); Anal. Calcd. for $C_{30}H_{22}FN_3O_2S$: C, 70.99; H, 4.37; N, 8.28; found C, 71.16; H, 4.33; N, 8.22.

2-(5-(4-Fluorophenyl)-3-(naphthalen-1-yl)-4,5-dihydro-1H-pyrazol-1-yl)-5-(4-methoxybenzylidene)thiazol-4(5H)-one ***9e***.

White crystals (yield 65%), m.p. 229-231 °C; IR (KBr, ν cm^{-1}) 1700 (C=O); ^{1}H NMR δ *ppm*: 3.66 (dd, 1H, CH_2a of pyrazoline ring, *J*= 4.0 Hz, 18.0 Hz), 3.83 (s, 3H, OCH_3), 4.38 (dd, 1H, CH_2b of pyrazoline ring, *J*= 11.2 Hz, 18.0 Hz), 5.90 (dd, 1H, CH of pyrazoline ring, *J*= 4.4 Hz, 11.2 Hz), 7.10-7.14 (m, 2H, Ar-H), 7.21-7.27 (m, 2H, Ar-H), 7.40-7.45 (m, 2H, Ar-H), 7.60-7.71 (m, 5H, Ar-H), 7.81-7.86 (m, 1H, Ar-H), 7.94 (d, 1H, Ar-H, *J*= 7.2 Hz), 8.08 (d, 1H, Ar-H, *J*= 7.6 Hz), 8.14 (d, 1H, Ar-H, *J*= 8.0 Hz), 9.18 (d, 1H, Ar-H, *J*= 8.4 Hz); ^{13}C NMR δ *ppm*: 46.55 (CH_2), 55.91 (OCH_3), 62.40 (CH), 115.37, 116.17, 116.39, 125.46, 125.73, 126.44, 126.52, 126.75,

127.01, 128.68, 128.77, 128.86, 129.55, 130.31, 130.83, 131.76, 132.13, 132.87, 134.11, 136.81, 136.84, 160.98, 161.14, 161.90, 171.09, 179.88 (C=O); Anal. Calcd. for $C_{30}H_{22}FN_3O_2S$: C, 70.99; H, 4.37; N, 8.28; found C, 71.22; H, 4.34; N, 8.21.

2-(5-(4-Fluorophenyl)-3-(naphthalen-1-yl)-4,5-dihydro-1H-pyrazol-1-yl)-5-(2-hydroxy-3-methoxybenzylidene)thiazol-4(5H)-one ***9f***.

White crystals (yield 73%), m.p. 243-245 °C; IR (KBr, ν cm^{-1}) 3213 (OH) and 1702 (C=O); ^{1}H NMR δ *ppm*: 3.19 (s, 3H, OCH_3), 3.65 (dd, 1H, CH_2a of pyrazoline ring, *J*= 4.0 Hz, 18.0 Hz), 4.38 (dd, 1H, CH_2b of pyrazoline ring, *J*= 11.2 Hz, 18.0 Hz), 5.91 (dd, 1H, CH of pyrazoline ring, *J*= 4.4 Hz, 11.2 Hz), 6.94 (t, 1H, Ar-H, *J*= 8.0 Hz), 7.06 (d, 1H, Ar-H, *J*= 8.0 Hz), 7.12 (d, 1H, Ar-H, *J*= 7.6 Hz), 7.21-7.27 (m, 2H, Ar-H), 7.41-7.45 (m, 2H, Ar-H), 7.60-7.68 (m, 2H, Ar-H), 7.78 (d, 1H, Ar-H, *J*= 7.6 Hz), 7.93 (d, 1H, Ar-H, *J*= 7.2 Hz), 8.01 (s, 1H, Ar-H), 8.06 (d, 1H, Ar-H, *J*= 8.0 Hz), 8.13 (d, 1H, Ar-H, *J*= 8.0 Hz), 9.15 (d, 1H, Ar-H, *J*= 8.8 Hz); ^{13}C NMR δ *ppm*: 49.07 (CH_2), 56.43 (OCH_3), 62.34 (CH), 116.39, 119.62, 120.13, 120.56, 121.54, 123.72, 125.77, 126.44, 127.06, 127.28, 128.84, 129.56, 130.27, 130.82, 131.99, 132.86, 134.10, 135.04, 137.50, 140.61, 143.04, 147.74, 151.54, 156.54, 166.61, 173.71, 179.91 (C=O); Anal. Calcd. for $C_{30}H_{22}FN_3O_3S$: C, 68.82; H, 4.24; N, 8.03; found C, 68.98; H, 4.21; N, 7.96.

2-(5-(4-Fluorophenyl)-3-(naphthalen-1-yl)-4,5-dihydro-1H-pyrazol-1-yl)-5-(3-hydroxy-4-methoxybenzylidene)thiazol-4(5H)-one ***9g***.

White crystals (yield 80%), m.p. 226-227 °C; IR (KBr, ν cm^{-1}) 3240 (OH) and 1712 (C=O); ^{1}H NMR δ *ppm*: 3.66 (dd, 1H, CH_2a of pyrazoline ring, *J*= 4.0 Hz, 18.0 Hz), 3.84 (s, 3H, OCH_3), 4.39 (dd, 1H, CH_2b of pyrazoline ring, *J*= 11.2 Hz, 18.0 Hz), 5.91 (dd, 1H, CH of pyrazoline ring, *J*= 4.0 Hz, 11.6 Hz), 7.07-7.10 (m, 1H, Ar-H), 7.13-7.16 (m, 2H, Ar-H), 7.21-7.27 (m, 2H, Ar-H), 7.41-7.46 (m, 2H, Ar-H), 7.56 (s, 1H, Ar-H), 7.61-7.71 (m, 2H, Ar-H), 7.81-7.85 (m, 1H, Ar-H), 7.94 (d, 1H, Ar-H, *J*= 7.6 Hz), 8.08 (d, 1H, Ar-H, *J*= 7.6 Hz), 8.14 (d, 1H, Ar-H, *J*= 8.0 Hz), 9.15 (d, 1H, Ar-H, *J*= 8.4 Hz), 9.51 (s, 1H, OH); ^{13}C NMR δ *ppm*: 46.60 (CH_2), 56.14 (OCH_3), 62.43 (CH), 112.98, 116.12, 116.18, 116.39, 123.47, 125.20, 125.75, 126.49, 126.52, 126.97, 127.05, 128.67, 128.75, 128.83, 129.55, 130.31, 130.74, 132.25, 132.82, 134.11, 136.87, 147.43, 150.18, 161.90, 171.19, 179.93 (C=O); Anal. Calcd. for $C_{30}H_{22}FN_3O_3S$: C, 68.82; H, 4.24; N, 8.03; found C, 68.64; H, 4.21; N, 8.09.

2-(5-(4-Fluorophenyl)-3-(naphthalen-1-yl)-4,5-dihydro-1H-pyrazol-1-yl)-5-(4-hydroxy-3-methoxybenzylidene)thiazol-4(5H)-one ***9h***.

White crystals (yield 74%), m.p. 229-230 °C; IR (KBr, ν cm^{-1}) 3230 (OH) and 1711 (C=O); ^{1}H NMR δ *ppm*: 3.64 (dd, 1H, CH_2a of pyrazoline ring, *J*= 4.0 Hz, 18.0 Hz), 3.85 (s, 3H, OCH_3), 4.38 (dd, 1H, CH_2b of pyrazoline ring, *J*= 11.2 Hz, 18.0 Hz), 5.90 (dd, 1H, CH of pyrazoline ring, *J*= 4.0 Hz, 11.6 Hz), 6.69 (d, 1H, Ar-H, *J*= 8.0 Hz), 7.06-7.10 (m, 2H, Ar-H), 7.20-7.25 (m, 2H, Ar-H), 7.38-7.43 (m, 2H, Ar-H), 7.55 (s, 1H, Ar-H), 7.59-7.71 (m, 3H, Ar-H), 7.90 (d, 1H, Ar-H, *J*= 8.0 Hz), 8.06 (d, 1H, Ar-H, *J*= 8.0 Hz), 8.11 (d, 1H, Ar-H, *J*= 8.0 Hz), 9.27 (d, 1H, Ar-H, *J*= 8.0 Hz), 9.50 (s, 1H, OH); ^{13}C NMR δ *ppm*: 46.40 (CH_2), 55.43 (OCH_3), 62.09 (CH), 112.77, 116.13, 116.34, 117.86, 119.01, 119.75, 125.75, 126.43, 126.68, 127.05, 128.48, 128.61, 128.69, 129.54, 130.31, 130.81, 132.72, 134.03, 134.16, 137.12, 137.15, 150.17, 160.93, 163.36, 170.74, 180.39 (C=O); Anal. Calcd. for $C_{30}H_{22}FN_3O_3S$: C, 68.82; H, 4.24; N, 8.03; found C, 69.07.88; H, 4.20; N, 8.00.

5-Benzylidene-2-(5-(4-chlorophenyl)-3-(naphthalen-1-yl)-4,5-dihydro-1H-pyrazol-1-yl)thiazol-4(5H)-one ***9i***.

White crystals (yield 75%), m.p. 217-218 °C; IR (KBr, ν cm^{-1}) 1701 (C=O); ^{1}H NMR δ *ppm*: 3.66 (dd, 1H, CH_2a of pyrazoline ring, *J*= 4.4 Hz, 18.0 Hz), 4.38 (dd, 1H, CH_2b of pyrazoline ring, *J*= 11.2 Hz, 18.0 Hz), 5.90 (dd, 1H, CH of pyrazoline ring, *J*= 4.4 Hz, 11.2 Hz), 7.39-7.42 (m, 2H, Ar-H), 7.43-7.48 (m, 3H, Ar-H), 7.52-7.56 (m, 2H, Ar-H), 7.360-7.68 (m, 4H, Ar-H), 7.70 (s, 1H, Ar-H), 7.79-7.83 (m, 1H, Ar-H), 7.92 (d, 1H, Ar-H, *J*= 7.6 Hz), 8.07 (d, 1H, Ar-H, *J*= 8.0 Hz), 8.13 (d, 1H, Ar-H, *J*= 8.0 Hz), 9.15 (d, 1H, Ar-H, *J*= 8.4 Hz); ^{13}C NMR δ *ppm*: 46.52 (CH_2), 62.52 (CH), 125.75, 126.36, 126.46, 127.07, 128.39, 128.52, 128.89, 129.40, 129.48, 129.56, 129.82, 130.20, 130.29, 130.49, 130.91, 131.81, 132.96, 133.11, 134.11, 134.31, 139.46, 139.83, 147.31, 151.83, 161.16, 162.33, 171.23, 179.59 (C=O); Anal. Calcd. for $C_{29}H_{20}ClN_3OS$: C, 70.51; H, 4.08; N, 8.51; found C, 70.40; H, 4.02; N, 8.55.

2-(5-(4-Chlorophenyl)-3-(naphthalen-1-yl)-4,5-dihydro-1H-pyrazol-1-yl)-5-(4-hydroxybenzylidene)thiazol-4(5H)-one ***9j***.

White crystals (yield 68%), m.p. 252-253 °C; IR (KBr, ν cm^{-1}) 3301 (OH) and 1702 (C=O); ^{1}H NMR δ *ppm*: 3.61 (dd, 1H, CH_2a of pyrazoline ring, *J*= 4.4 Hz, 18.0 Hz), 4.30 (dd, 1H, CH_2b of

pyrazoline ring, *J*= 11.2 Hz, 18.0 Hz), 5.80 (dd, 1H, CH of pyrazoline ring, *J*= 4.4 Hz, 11.2 Hz), 6.98-6.91 (m, 1H, Ar-H), 7.32-7.41 (m, 2H, Ar-H), 7.33-7.41 (m, 3H, Ar-H), 7.58-7.68 (m, 3H, Ar-H), 7.80-7.85 (m, 2H, Ar-H), 7.88-7.92 (m, 2H, Ar-H), 8.05-8.14 (m, 2H, Ar-H), 9.17-9.22 (m, 1H, Ar-H); ^{13}C NMR δ *ppm*: 46.26 (CH_2), 62.27 (CH), 117.27, 125.73, 126.30, 126.51, 127.02, 128.38, 128.45, 128.61, 128.86, 129.08, 129.39, 129.44, 129.55, 130.28, 130.71, 130.90, 132.56, 132.82, 132.97, 134.10, 134.13, 139.66, 139.82, 161.14, 171.09, 178.26 (C=O); Anal. Calcd. for $C_{29}H_{20}ClN_3O_2S$: C, 68.30; H, 3.95; N, 8.24; found C, 68.49; H, 3.93; N, 8.20.

2-(5-(4-Chlorophenyl)-3-(naphthalen-1-yl)-4,5-dihydro-1H-pyrazol-1-yl)-5-(4-(dimethylamino)benzylidene)thiazol-4(5H)-one ***9k***.

White crystals (yield 75%), m.p. 241-243 °C; IR (KBr, ν cm^{-1}) 1710 (C=O); ^{1}H NMR δ *ppm*: 3.10 (s, 6H, $N(CH_3)_2$), 3.64 (dd, 1H, CH_2a of pyrazoline ring, *J*= 4.4 Hz, 18.0 Hz), 4.37 (dd, 1H, CH_2b of pyrazoline ring, *J*= 11.2 Hz, 18.0 Hz), 5.88 (dd, 1H, CH of pyrazoline ring, *J*= 4.4 Hz, 11.2 Hz), 6.82-8.86 (m, 2H, Ar-H), 7.38-7.41 (m, 2H, Ar-H), 7.44-7.57 (m, 4H, Ar-H), 7.58 (s, 1H, Ar-H), 7.60 (t, 1H, Ar-H, *J*= 8.0 Hz), 7.66 (t, 1H, Ar-H, *J*= 8.0 Hz), 7.82 (t, 1H, Ar-H, *J*= 7.2 Hz), 7.92 (d, 1H, Ar-H, *J*= 7.2 Hz), 8.08 (d, 1H, Ar-H, *J*= 8.4 Hz), 8.13 (d, 1H, Ar-H, *J*= 8.0 Hz), 9.20 (d, 1H, Ar-H, *J*= 8.8 Hz); ^{13}C NMR δ *ppm*: 46.40 (CH_2), 49.07 ($N(CH_3)_2$), 62.32 (CH), 112.64, 121.08, 122.92, 125.76, 126.48, 127.02, 128.45, 129.45, 129.56, 130.30, 130.75, 132.17, 132.78, 133.01, 134.12, 137.52, 138.26, 139.73, 142.06, 142.78, 144.81, 151.74, 170.89, 180.21 (C=O); Anal. Calcd. for $C_{31}H_{25}ClN_4OS$: C, 69.33; H, 4.69; N, 10.43; found C, 69.25; H, 4.70; N, 10.40.

2-(5-(4-Chlorophenyl)-3-(naphthalen-1-yl)-4,5-dihydro-1H-pyrazol-1-yl)-5-(2-methoxybenzylidene)thiazol-4(5H)-one ***9l***.

White crystals (yield 78%), m.p. 209-211 °C; IR (KBr, ν cm^{-1}) 1705 (C=O); ^{1}H NMR δ *ppm*: 3.63 (dd, 1H, CH_2a of pyrazoline ring, *J*= 4.0 Hz, 18.0 Hz), 3.82 (s, 3H, OCH_3), 4.36 (dd, 1H, CH_2b of pyrazoline ring, *J*= 11.2 Hz, 18.0 Hz), 5.91 (dd, 1H, CH of pyrazoline ring, *J*= 4.0 Hz, 11.6 Hz), 6.68 (d, 1H, Ar-H, *J*= 8.0 Hz), 7.06-7.10 (m, 2H, Ar-H), 7.20-7.25 (m, 2H, Ar-H), 7.37-7.44 (m, 3H, Ar-H), 7.56 (s, 1H, Ar-H), 7.59-7.71 (m, 3H, Ar-H), 7.90 (d, 1H, Ar-H, *J*= 8.0 Hz), 8.06 (d, 1H, Ar-H, *J*= 8.0 Hz), 8.10 (d, 1H, Ar-H, *J*= 8.0 Hz), 9.25 (d, 1H, Ar-H, *J*= 8.0 Hz); ^{13}C NMR δ *ppm*: 49.07 (CH_2), 56.14 (OCH_3), 62.39 (CH), 112.28, 121.54, 122.82, 125.76, 126.31,

127.08, 128.48, 128.82, 129.17, 129.47, 129.57, 130.26, 130.94, 132.44, 132.96, 133.09, 134.10, 135.13, 137.50, 139.52, 158.34, 162.10, 171.40, 179.68 (C=O); Anal. Calcd. for $C_{30}H_{22}ClN_3O_2S$: C, 68.76; H, 4.23; N, 8.02; found C, 68.51; H, 4.20; N, 8.07.

2-(5-(4-Chlorophenyl)-3-(naphthalen-1-yl)-4,5-dihydro-1H-pyrazol-1-yl)-5-(4-methoxybenzylidene)thiazol-4(5H)-one ***9m***.

White crystals (yield 75%), m.p. 249-250 °C; IR (KBr, ν cm^{-1}) 1707 (C=O); ^{1}H NMR δ *ppm*: 3.62 (dd, 1H, CH_2a of pyrazoline ring, *J*= 4.0 Hz, 18.0 Hz), 3.71 (s, 3H, OCH_3), 4.31 (dd, 1H, CH_2b of pyrazoline ring, *J*= 11.2 Hz, 18.0 Hz), 5.79 (dd, 1H, CH of pyrazoline ring, *J*= 4.0 Hz, 11.2 Hz), 7.10-7.14 (m, 2H, Ar-H), 7.32-7.35 (m, 2H, Ar-H), 7.38-7.48 (m, 3H, Ar-H), 7.59-7.76 (m, 4H, Ar-H), 7.82-7.95 (m, 2H, Ar-H), 8.06-8.16 (m, 2H, Ar-H), 9.17-9.25 (m, 1H, Ar-H); ^{13}C NMR δ *ppm*: 46.25 (CH_2), 55.92 (OCH_3), 62.26 (CH), 115.38, 125.42, 125.74, 126.29, 126.38, 126.52, 126.72, 127.03, 128.38, 128.48, 128.88, 129.40, 129.46, 129.56, 130.27, 130.91, 132.15, 132.83, 132.96, 133.08, 134.10, 134.12, 139.82, 161.14, 161.89, 171.12, 178.25 (C=O); Anal. Calcd. for $C_{30}H_{22}ClN_3O_2S$: C, 68.76; H, 4.23; N, 8.02; found C, 68.86; H, 4.28; N, 8.00.

2-(5-(4-Chlorophenyl)-3-(naphthalen-1-yl)-4,5-dihydro-1H-pyrazol-1-yl)-5-(2-hydroxy-3-methoxybenzylidene)thiazol-4(5H)-one ***9n***.

White crystals (yield 70%), m.p. 234-235 °C; IR (KBr, ν cm^{-1}) 3202 (OH) and 1701 (C=O); ^{1}H NMR δ *ppm*: 3.64 (dd, 1H, CH_2a of pyrazoline ring, *J*= 4.4 Hz, 18.0 Hz), 3.85 (s, 3H, OCH_3), 4.36 (dd, 1H, CH_2b of pyrazoline ring, *J*= 11.2 Hz, 18.0 Hz), 5.90 (dd, 1H, CH of pyrazoline ring, *J*= 4.4 Hz, 11.2 Hz), 6.93 (t, 1H, Ar-H, *J*= 8.0 Hz), 7.05 (d, 1H, Ar-H, *J*= 8.0 Hz), 7.12 (d, 1H, Ar-H, *J*= 8.0 Hz), 7.38-7.41(m, 2H, Ar-H), 7.44-7.47 (m, 2H, Ar-H), 7.58-7.67 (m, 2H, Ar-H), 7.77 (t, 1H, Ar-H, *J*= 8.8 Hz), 7.91 (d, 1H, Ar-H, *J*= 7.2 Hz), 8.03 (s, 1H, Ar-H), 8.05 (d, 1H, Ar-H, *J*= 8.0 Hz), 8.11 (d, 1H, Ar-H, *J*= 8.4 Hz), 9.15 (d, 1H, Ar-H, *J*= 8.8 Hz); ^{13}C NMR δ *ppm*: 46.40 (CH_2), 56.45 (OCH_3), 62.37 (CH), 113.94, 120.01, 120.08, 121.63, 125.71, 126.37, 126.48, 127.02, 127.09, 127.38, 128.43, 128.79, 129.45, 129.52, 130.28, 130.79, 132.86, 133.10, 134.09, 139.52, 146.93, 148.50, 161.83, 171.44, 179.84 (C=O); Anal. Calcd. for $C_{30}H_{22}ClN_3O_3S$: C, 66.72; H, 4.11; N, 7.78; found C, 66.60; H, 4.14; N, 7.72.

2-(5-(4-Chlorophenyl)-3-(naphthalen-1-yl)-4,5-dihydro-1H-pyrazol-1-yl)-5-(3-hydroxy-4-methoxybenzylidene)thiazol-4(5H)-one ***9o***.

White crystals (yield 73%), m.p. 265-266 °C; IR (KBr, *ν* cm^{-1}) 3220 (OH) and 1712 (C=O); ^{1}H NMR *δ ppm*: 3.65 (dd, 1H, CH_2a of pyrazoline ring, *J*= 4.4 Hz, 18.0 Hz), 3.84 (s, 3H, OCH_3), 4.39 (dd, 1H, CH_2b of pyrazoline ring, *J*= 11.6 Hz, 18.4 Hz), 5.91 (dd, 1H, CH of pyrazoline ring, *J*= 4.4 Hz, 11.2 Hz), 7.07-7.09 (m, 1H, Ar-H), 7.13-7.15 (m, 2H, Ar-H), 7.39-7.42 (m, 2H, Ar-H), 7.46-7.48 (m, 2H, Ar-H), 7.57 (s, 1H, Ar-H), 7.61-7.70 (m, 2H, Ar-H), 7.08 (t, 1H, Ar-H, *J*= 8.8 Hz), 7.93 (d, 1H, Ar-H, *J*= 8.0 Hz), 8.08 (d, 1H, Ar-H, *J*= 8.8 Hz), 8.14 (d, 1H, Ar-H, *J*= 8.0 Hz), 9.17 (d, 1H, Ar-H, *J*= 8.0 Hz), 9.52 (s, 1H, OH); ^{13}C NMR *δ ppm*: 46.50 (CH_2), 56.15 (OCH_3), 62.45 (CH), 112.98, 116.14, 123.46, 125.18, 125.75, 126.48, 126.95, 127.06, 128.47, 128.83, 129.47, 129.55, 130.29, 130.74, 132.32, 132.83, 133.07, 134.10, 139.57, 147.43, 150.20, 161.86, 171.23, 179.90 (C=O); Anal. Calcd. for $C_{30}H_{22}ClN_3O_3S$: C, 66.72; H, 4.11; N, 7.78; found C, 66.61; H, 4.06; N, 7.72.

2-(5-(4-Chlorophenyl)-3-(naphthalen-1-yl)-4,5-dihydro-1H-pyrazol-1-yl)-5-(4-hydroxy-3-methoxybenzylidene)thiazol-4(5H)-one ***9p***.

White crystals (yield 76%), m.p. 256-258 °C; IR (KBr, *ν* cm^{-1}) 3213 (OH) and 1702 (C=O); ^{1}H NMR *δ ppm*: 3.65 (dd, 1H, CH_2a of pyrazoline ring, *J*= 4.4 Hz, 18.0 Hz), 3.82 (s, 3H, OCH_3), 4.39 (dd, 1H, CH_2b of pyrazoline ring, *J*= 11.6 Hz, 18.4 Hz), 5.91 (dd, 1H, CH of pyrazoline ring, *J*= 4.4 Hz, 11.2 Hz), 6.88-6.70 (m, 1H, Ar-H), 7.06-7.11 (m, 2H, Ar-H), 7.37-7.40 (m, 2H, Ar-H), 7.45-7.48 (m, 2H, Ar-H), 7.55 (s, 1H, Ar-H), 7.60-7.76 (m, 3H, Ar-H), 7.91-7.94 (m, 1H, Ar-H), 8.06-8.09 (m, 1H, Ar-H), 8.13 (d, 1H, Ar-H, *J*= 8.0 Hz), 9.22 (d, 1H, Ar-H, *J*= 8.0 Hz); ^{13}C NMR *δ ppm*: 46.34 (CH_2), 55.54(OCH_3), 62.15 (CH), 113.06, 117.74, 123.56, 124.48, 125.77, 126.39, 126.63, 126.75, 127.09, 128.45, 128.53, 129.43, 129.56, 130.29, 130.85, 132.77, 132.98, 133.84, 134.15, 139.84, 140.35, 145.38, 149.84, 160.68, 170.75, 180.26 (C=O); Anal. Calcd. for $C_{30}H_{22}ClN_3O_3S$: C, 66.72; H, 4.11; N, 7.78; found C, 66.47; H, 4.09; N, 7.75.

6.1. Molecular Dynamics

The receptor and ligand topologies were generated by PDB2gmx (embedded in GROMACS) and GlycoBioChem PRODRG2 Server respectively, both under GROMOS96 force field was implemented to generate the ligand topologies using the [40]. After rejoining ligands and receptor topologies to generate four systems, the typical molecular dynamics scheme of GROMACS was applied for all the systems. This include, solvation, neutralization, energy minimization under GROMOS96 43a1 force field and two stages of equilibration (NVT and NPT) [41-44]. Finally, unrestricted production stage of 200ns was applied for the four systems with particle mesh ewald (PME) method implemented to compute the long-range electrostatic values using 12 Å cut-off and 12 Å Fourier spacing. Further information is available in the supplementary material section. The stability of the complexes was judged using RMSD and RMSF values calculated from the MDS trajectories from the production step.

6.2. MMPBSA calculations

$$\Delta G_{(Binding)} = G_{(Complex)} - G_{(Receptor)} - G_{(Ligand)}$$

Where $G_{(Complex)}$ is the total free energy of the protein−ligand complex and $G_{(Receptor)}$ and $G_{(Ligand)}$ are the total free energies of the isolated protein and ligand in solvent, respectively. The total free energy of any of the three mentioned entities (complex, receptor and ligand) were calculated for all MD trajectories from its molecular mechanics potential energy plus the energy of the solvation, using the g_mmpbsa package implemented in the GROMACS software. Individual energies along with the values of standard deviations were calculated and then summed together to yield the average total free energy of each component. Finally, to calculate the binding-free energy, the total free energy of the receptor and the ligand were subtracted from the total free energy of the complex.

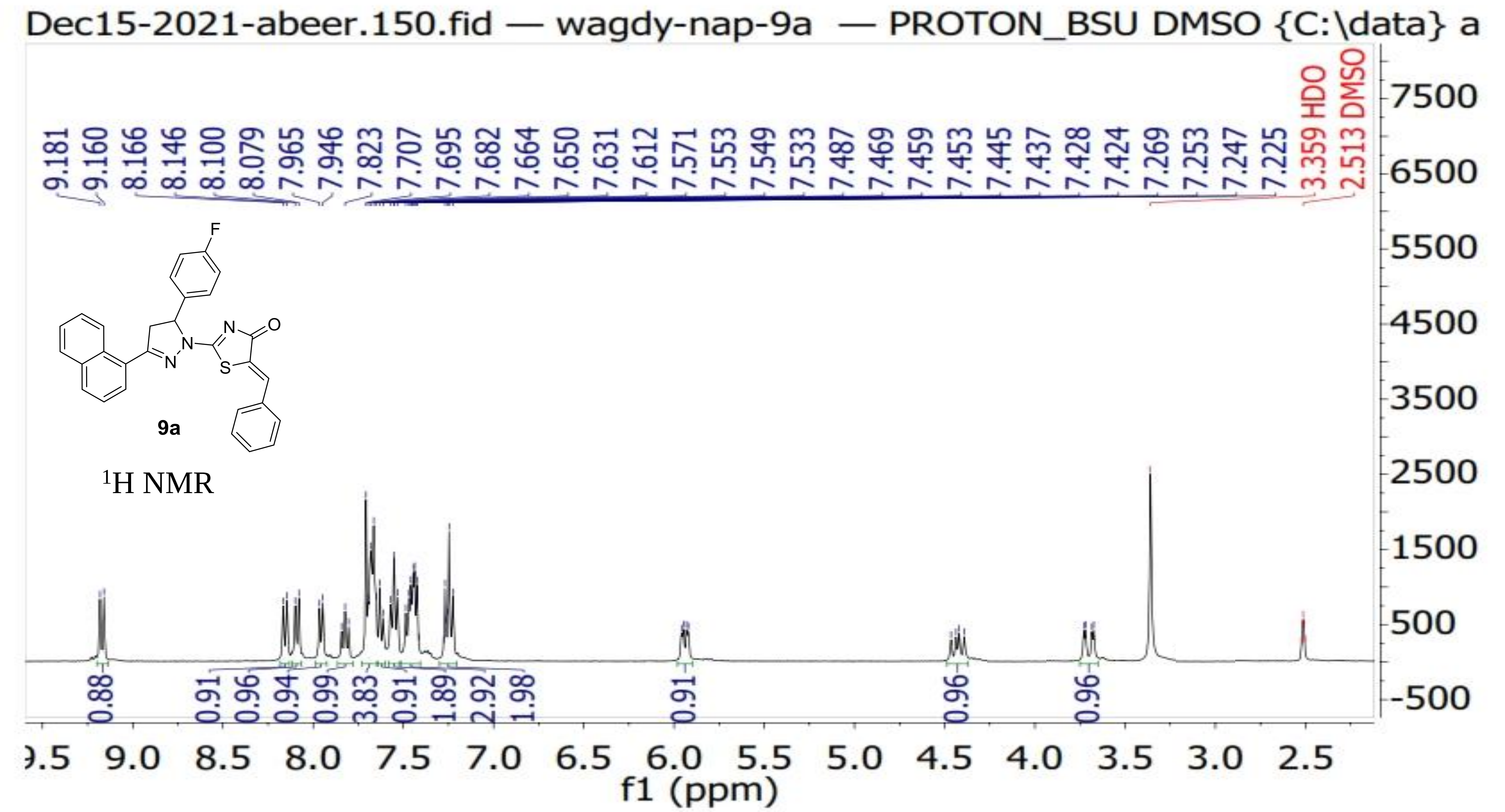
Dec15-2021-abeer.150.fid — wagdy-nap-9a — PROTON_BSU DMSO {C:\data} a
9.181
9.160
8.166
8.146
8.100
8.079
7.965
7.946
7.823
7.707
7.695
7.682
7.664
7.650
7.631
7.612
7.571
7.553
7.549
7.533
7.487
7.469
7.459
7.453
7.445
7.437
7.428
7.424
7.269
7.253
7.247
7.225
3.359 HDO
2.513 DMSO
F
N
O
N
N
S
9a
1H NMR
7500
6500
5500
4500
3500
2500
1500
500
-500
0.88
0.91
0.96
0.94
0.99
3.83
0.91
1.89
2.92
1.98
0.91
0.96
0.96
9.5
9.0
8.5
8.0
7.5
7.0
6.5
6.0
5.5
5.0
4.5
4.0
3.5
3.0
2.5
f1 (ppm)

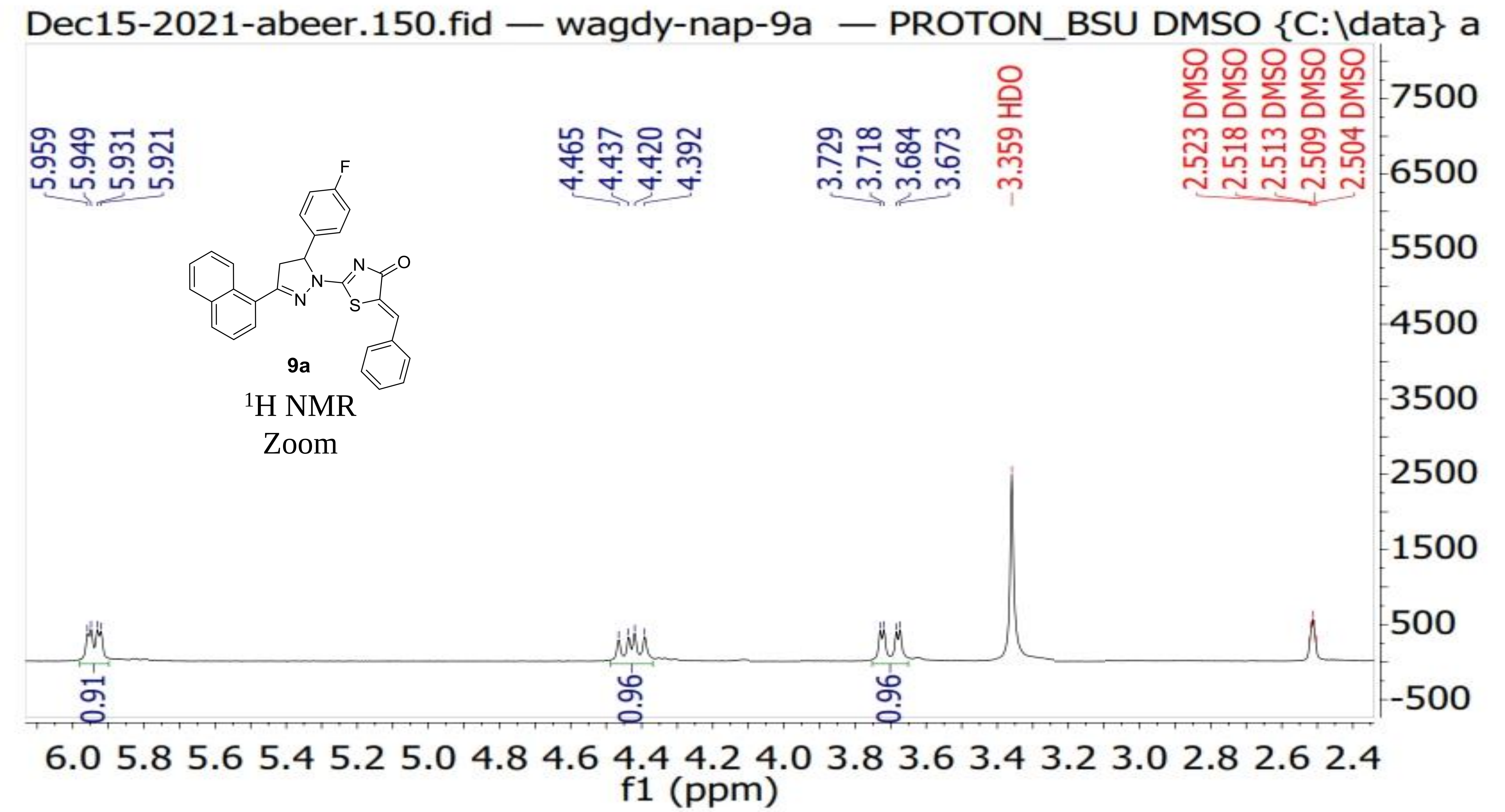
Dec15-2021-abeer.150.fid — wagdy-nap-9a — PROTON_BSU DMSO {C:\data} a
5.959
5.949
5.931
5.921
4.465
4.437
4.420
4.392
3.729
3.718
3.684
3.673
3.359 HDO
2.523 DMSO
2.518 DMSO
2.513 DMSO
2.509 DMSO
2.504 DMSO
F
N
O
N
N
S
9a
1H NMR
Zoom
0.91
0.96
0.96
7500
6500
5500
4500
3500
2500
1500
500
-500
6.0 5.8 5.6 5.4 5.2 5.0 4.8 4.6 4.4 4.2 4.0 3.8 3.6 3.4 3.2 3.0 2.8 2.6 2.4
f1 (ppm)

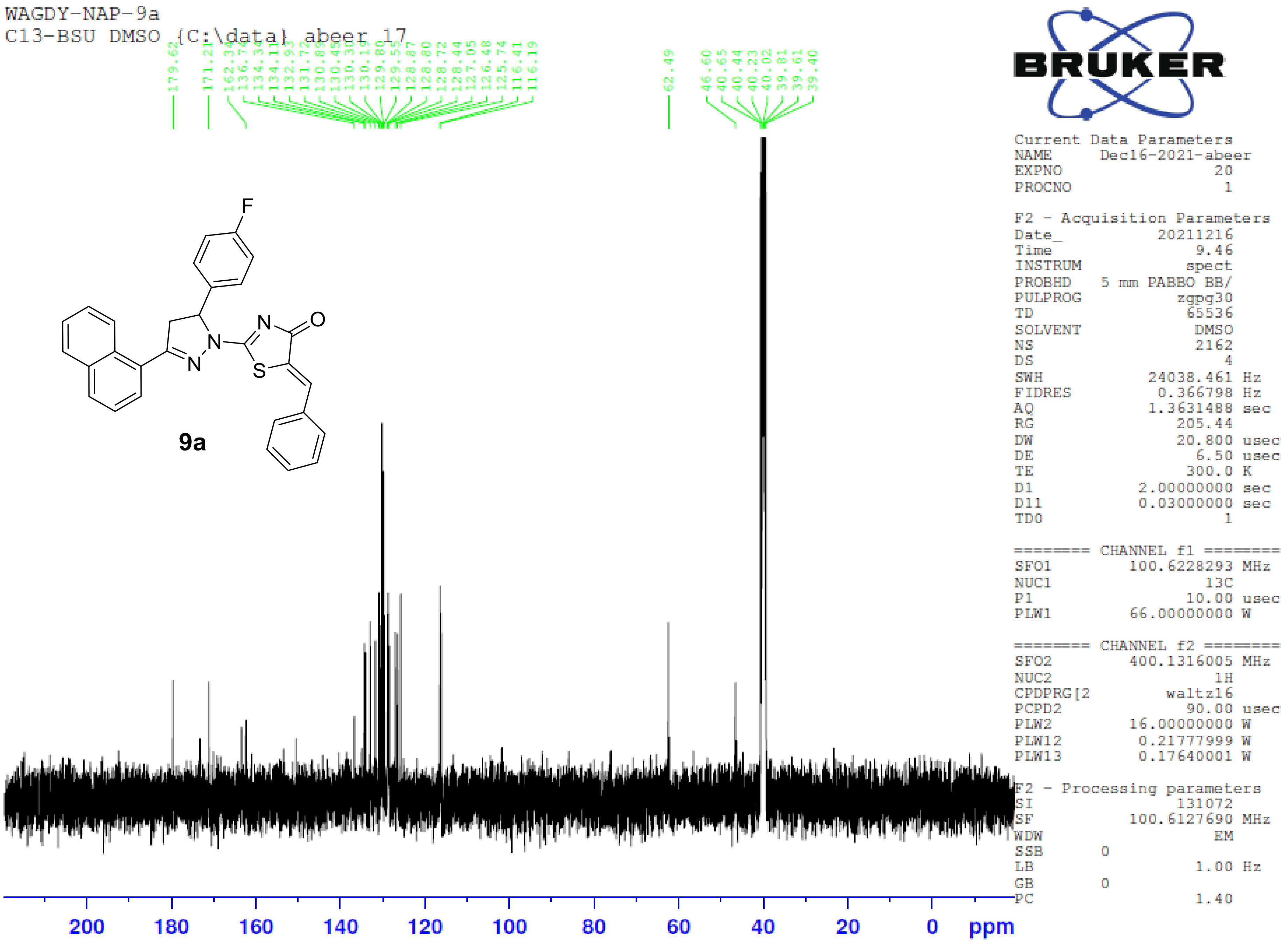
WAGDY-NAP-9a
C13-BSU DMSO {C:\data} abeer 17
179.62
171.21
162.34
136.74
134.34
134.11
132.93
131.72
130.89
130.45
130.30
130.15
129.80
129.55
128.87
128.80
128.72
128.44
127.05
126.48
125.74
116.41
116.19
62.49
46.60
40.65
40.44
40.23
40.02
39.81
39.61
39.40
F
N
N
N
O
S
9a
200
180
160
140
120
100
80
60
40
20
0
ppm
Current Data Parameters
NAME Dec16-2021-abeer
EXPNO 20
PROCNO 1
F2 - Acquisition Parameters
Date_ 20211216
Time 9.46
INSTRUM spect
PROBHD 5 mm PABBO BB/
PULPROG zgpg30
TD 65536
SOLVENT DMSO
NS 2162
DS 4
SWH 24038.461 Hz
FIDRES 0.366798 Hz
AQ 1.3631488 sec
RG 205.44
DW 20.800 usec
DE 6.50 usec
TE 300.0 K
D1 2.00000000 sec
D11 0.03000000 sec
TD0 1
======== CHANNEL f1 ========
SFO1 100.6228293 MHz
NUC1 13C
P1 10.00 usec
PLW1 66.00000000 W
======== CHANNEL f2 ========
SFO2 400.1316005 MHz
NUC2 1H
CPDPRG[2 waltz16
PCPD2 90.00 usec
PLW2 16.00000000 W
PLW12 0.21777999 W
PLW13 0.17640001 W
F2 - Processing parameters
SI 131072
SF 100.6127690 MHz
WDW EM
SSB 0
LB 1.00 Hz
GB 0
PC 1.40

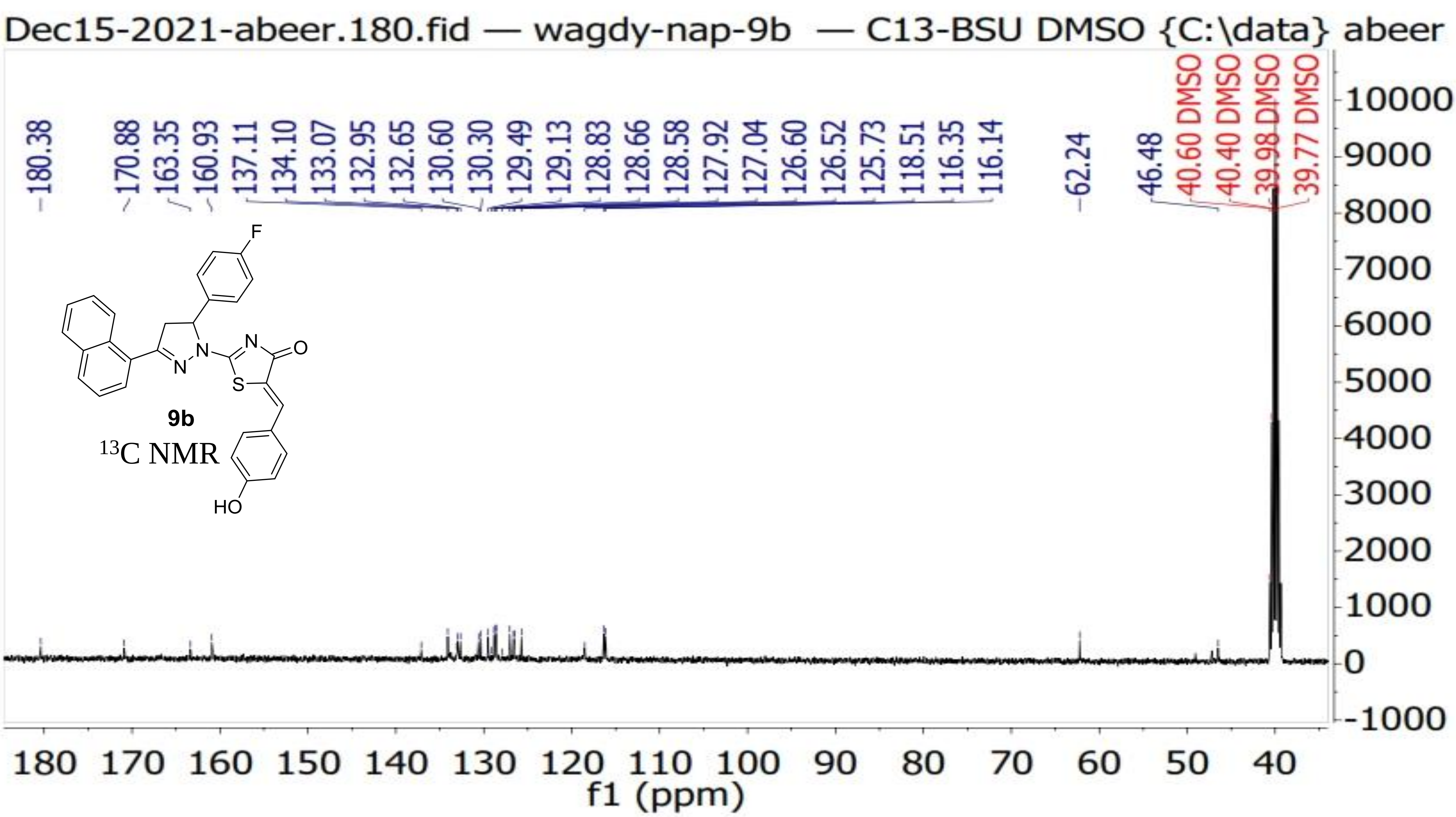

Dec15-2021-abeer.180.fid — wagdy-nap-9b — C13-BSU DMSO {C:\data} abeer
180.38
170.88
163.35
160.93
137.11
134.10
133.07
132.95
132.65
130.60
130.30
129.49
129.13
128.83
128.66
128.58
127.92
127.04
126.60
126.52
125.73
118.51
116.35
116.14
62.24
46.48
40.60 DMSO
40.40 DMSO
39.98 DMSO
39.77 DMSO
F
N
N
N
O
S
HO
9b
^{13}C NMR
f1 (ppm)

wagdy-nap-9c
C13-BSU DMSO {C:\data} abeer 18

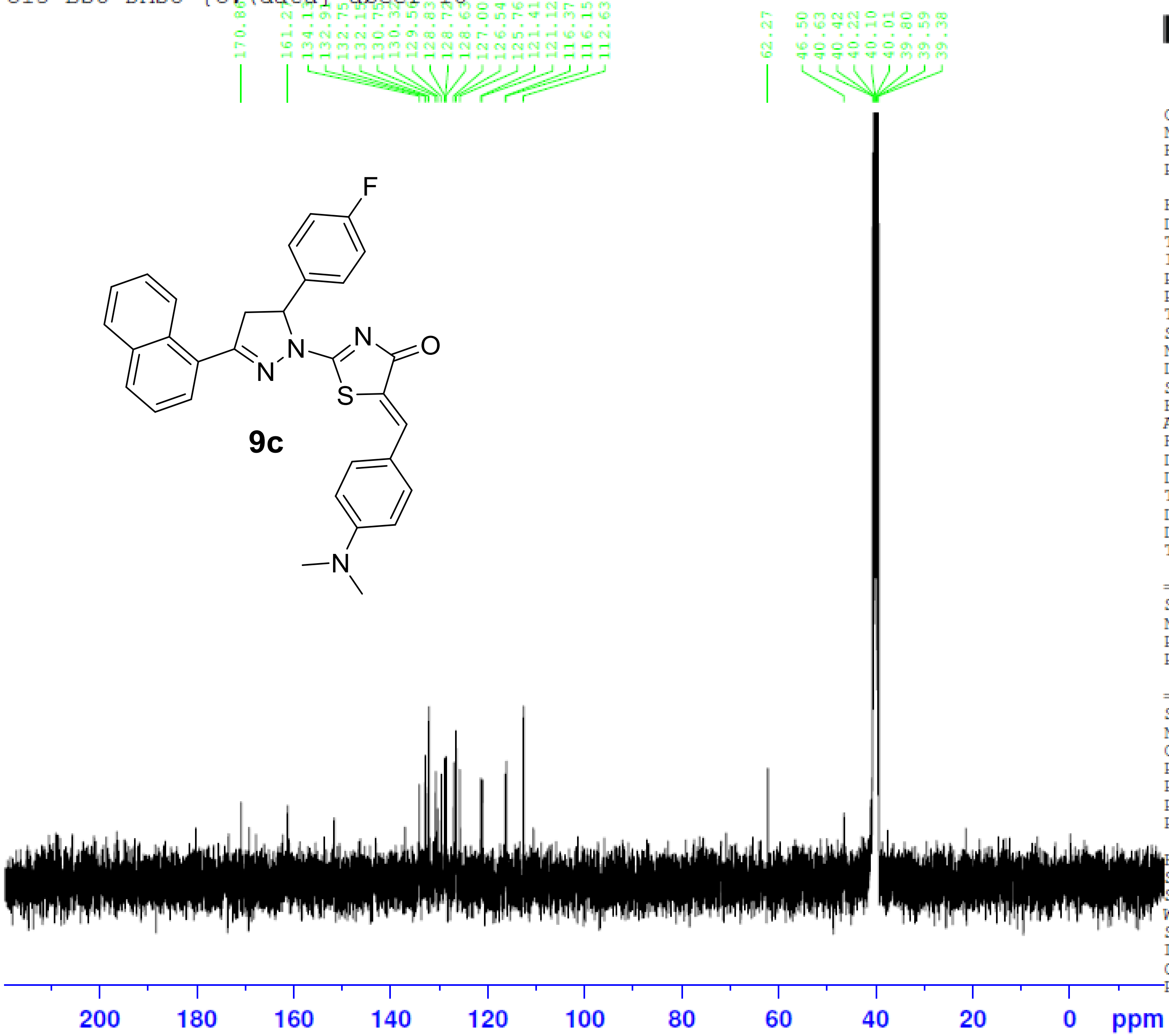


```
Current Data Parameters
NAME        Dec16-2021-abeer
EXPNO                     30
PROCNO                     1

F2 - Acquisition Parameters
Date_               20211216
Time                   11.53
INSTRUM                spect
PROBHD     5 mm PABBO BB/
PULPROG              zgpg30
TD                     65536
SOLVENT                 DMSO
NS                      2104
DS                         4
SWH            24038.461 Hz
FIDRES          0.366798 Hz
AQ             1.3631488 sec
RG                    205.44
DW                20.800 usec
DE                  6.50 usec
TE                   300.1 K
D1           2.00000000 sec
D11          0.03000000 sec
TD0                        1

======== CHANNEL f1 ========
SFO1        100.6228293 MHz
NUC1                     13C
P1                 10.00 usec
PLW1          66.00000000 W

======== CHANNEL f2 ========
SFO2        400.1316005 MHz
NUC2                      1H
CPDPRG[2             waltz16
PCPD2              90.00 usec
PLW2          16.00000000 W
PLW12          0.21777999 W
PLW13          0.17640001 W

F2 - Processing parameters
SI                    131072
SF          100.6127690 MHz
WDW                       EM
SSB        0
LB                   1.00 Hz
GB         0
PC                      1.40
```

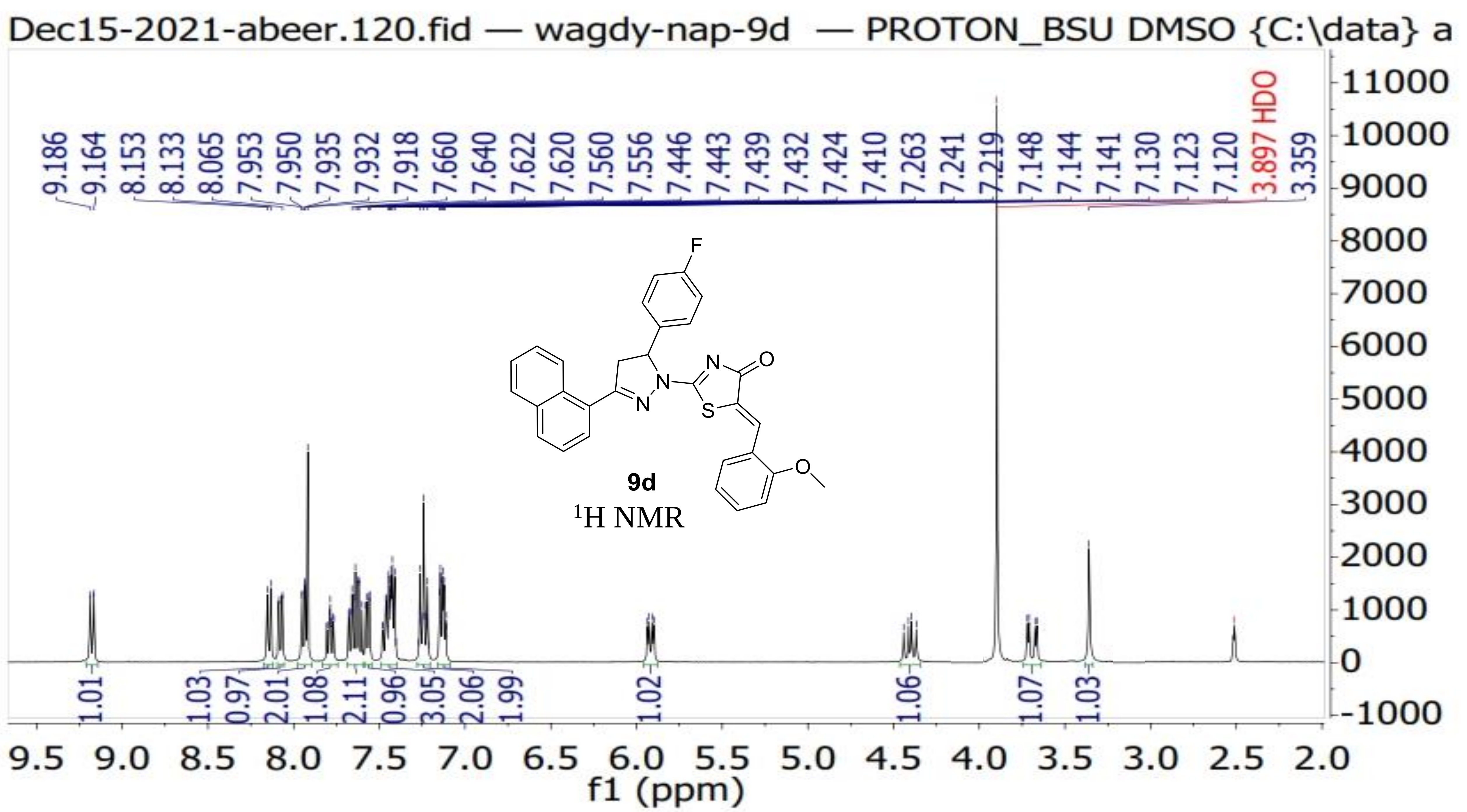
Dec15-2021-abeer.120.fid — wagdy-nap-9d — PROTON_BSU DMSO {C:\data} a
9.186 9.164 8.153 8.133 8.065 7.953 7.950 7.935 7.932 7.918 7.660 7.640 7.622 7.620 7.560 7.556 7.446 7.443 7.439 7.432 7.424 7.410 7.263 7.241 7.219 7.148 7.144 7.141 7.130 7.123 7.120 3.897 HDO 3.359
F
N
O
N
S
N
O
9d
1H NMR
1.01 1.03 0.97 2.01 1.08 2.11 0.96 3.05 2.06 1.99 1.02 1.06 1.07 1.03
11000 10000 9000 8000 7000 6000 5000 4000 3000 2000 1000 0 -1000
9.5 9.0 8.5 8.0 7.5 7.0 6.5 6.0 5.5 5.0 4.5 4.0 3.5 3.0 2.5 2.0
f1 (ppm)

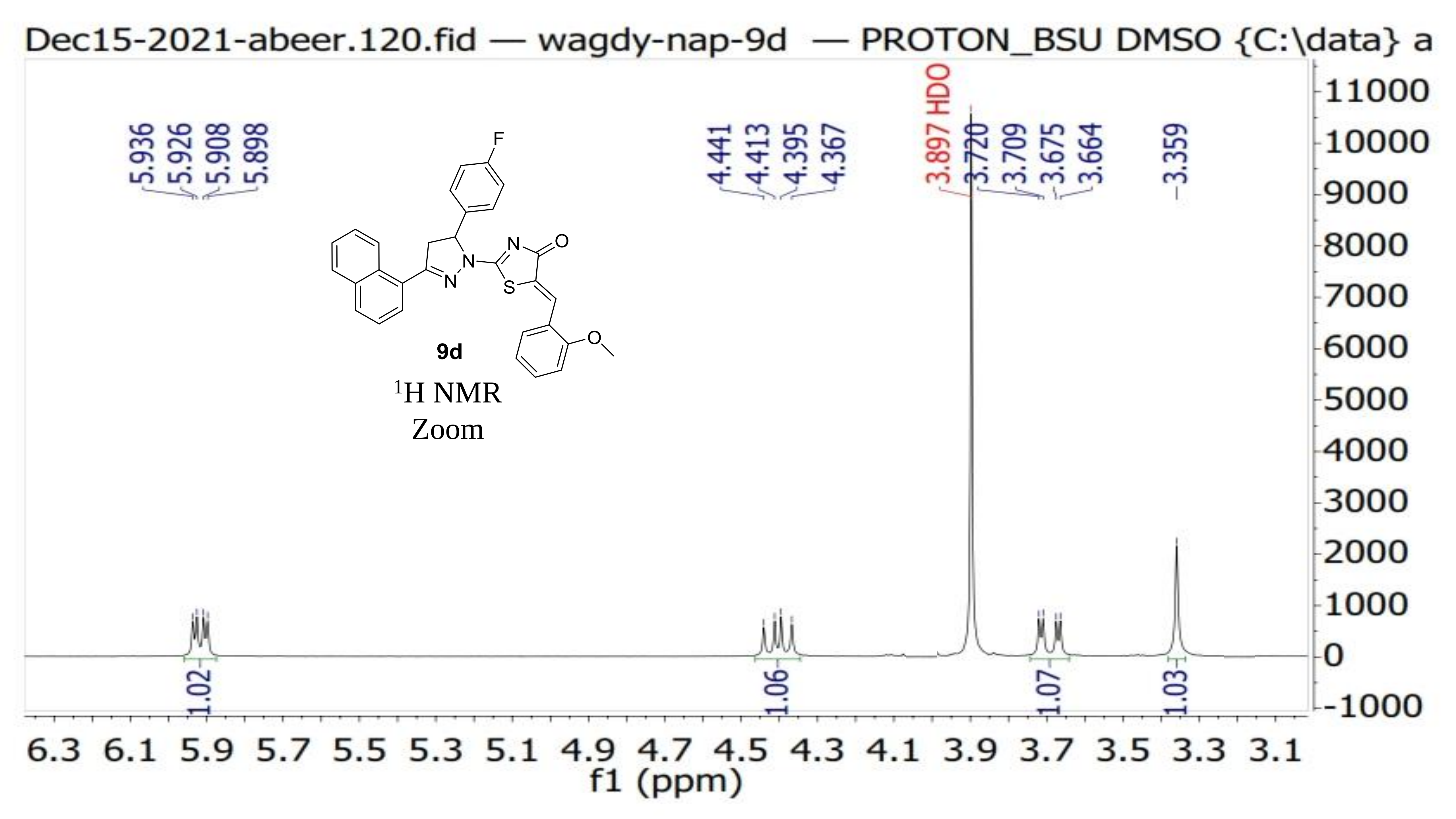
Dec15-2021-abeer.120.fid — wagdy-nap-9d — PROTON_BSU DMSO {C:\data} a
5.936
5.926
5.908
5.898
4.441
4.413
4.395
4.367
3.897 HDO
3.720
3.709
3.675
3.664
3.359
F
N
O
N
N
S
O
9d
^{1}H NMR
Zoom
1.02
1.06
1.07
1.03
11000
10000
9000
8000
7000
6000
5000
4000
3000
2000
1000
0
-1000
6.3 6.1 5.9 5.7 5.5 5.3 5.1 4.9 4.7 4.5 4.3 4.1 3.9 3.7 3.5 3.3 3.1
f1 (ppm)

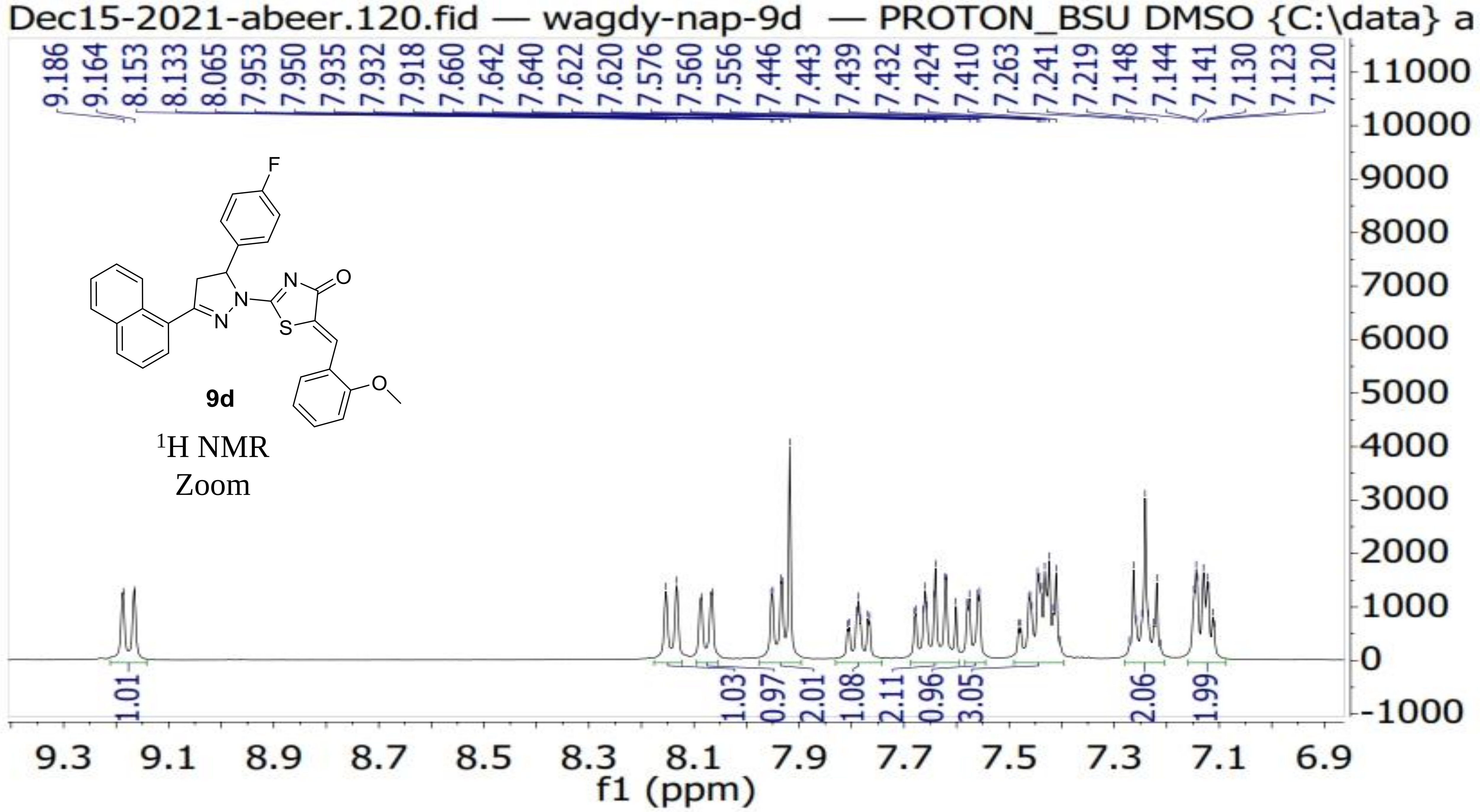
Dec15-2021-abeer.120.fid — wagdy-nap-9d — PROTON_BSU DMSO {C:\data} a
9.186 9.164 8.153 8.133 8.065 7.953 7.950 7.935 7.932 7.918 7.660 7.642 7.640 7.622 7.620 7.576 7.560 7.556 7.446 7.443 7.439 7.432 7.424 7.410 7.263 7.241 7.219 7.148 7.144 7.141 7.130 7.123 7.120
F
N
O
N
N
S
O
9d
1H NMR
Zoom
1.01
1.03
0.97
2.01
1.08
2.11
0.96
3.05
2.06
1.99
11000
10000
9000
8000
7000
6000
5000
4000
3000
2000
1000
0
-1000
9.3
9.1
8.9
8.7
8.5
8.3
8.1
7.9
7.7
7.5
7.3
7.1
6.9
f1 (ppm)

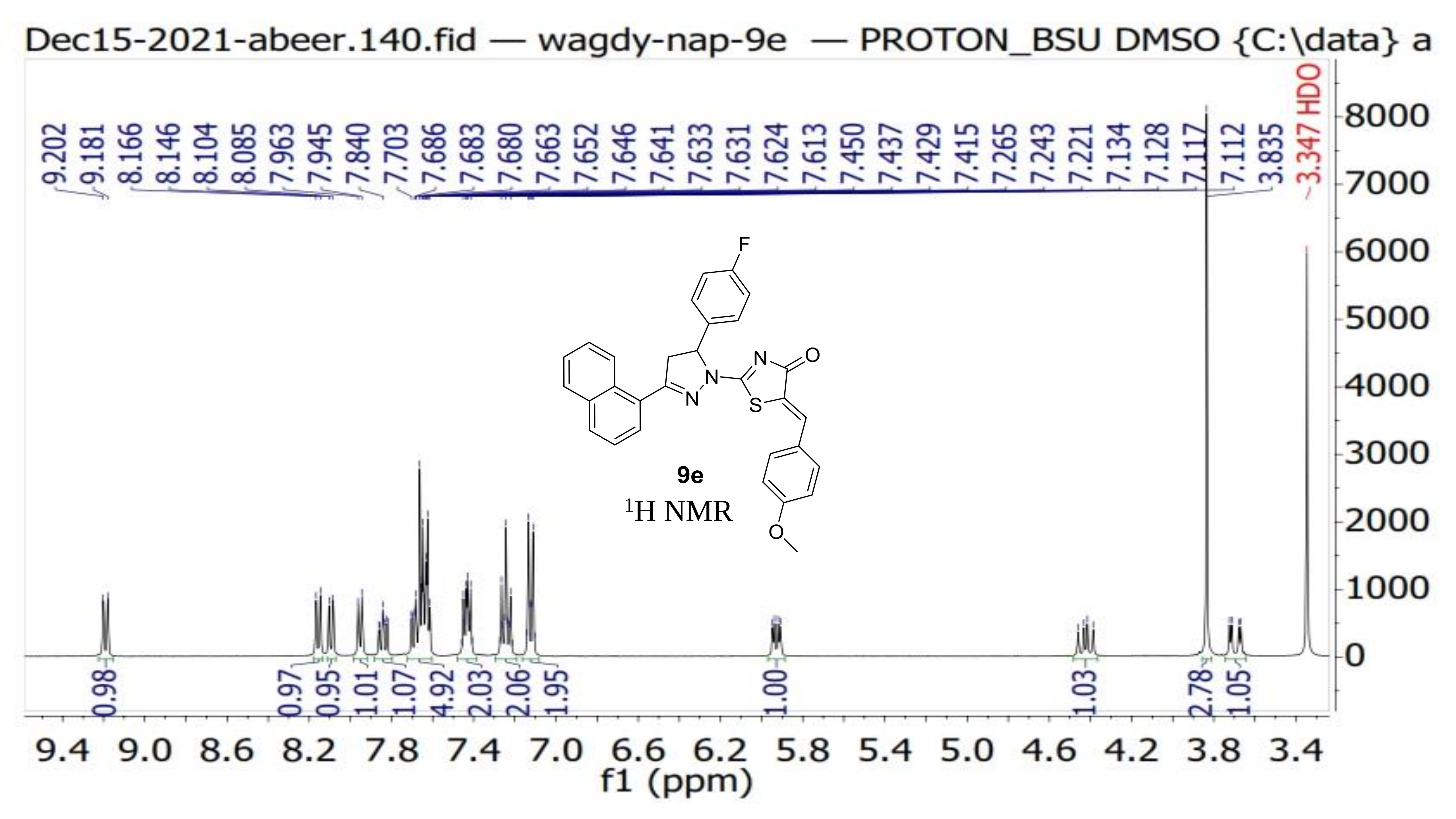
Dec15-2021-abeer.140.fid — wagdy-nap-9e — PROTON_BSU DMSO {C:\data} a
9.202
9.181
8.166
8.146
8.104
8.085
7.963
7.945
7.840
7.703
7.686
7.683
7.680
7.663
7.652
7.646
7.641
7.633
7.631
7.624
7.613
7.450
7.437
7.429
7.415
7.265
7.243
7.221
7.134
7.128
7.117
7.112
3.835
~3.347 HDO
F
N
O
N
N
S
O
9e
1H NMR
0.98
0.97
0.95
1.01
1.07
4.92
2.03
2.06
1.95
1.00
1.03
2.78
1.05
8000
7000
6000
5000
4000
3000
2000
1000
0
9.4
9.0
8.6
8.2
7.8
7.4
7.0
6.6
6.2
5.8
5.4
5.0
4.6
4.2
3.8
3.4
f1 (ppm)

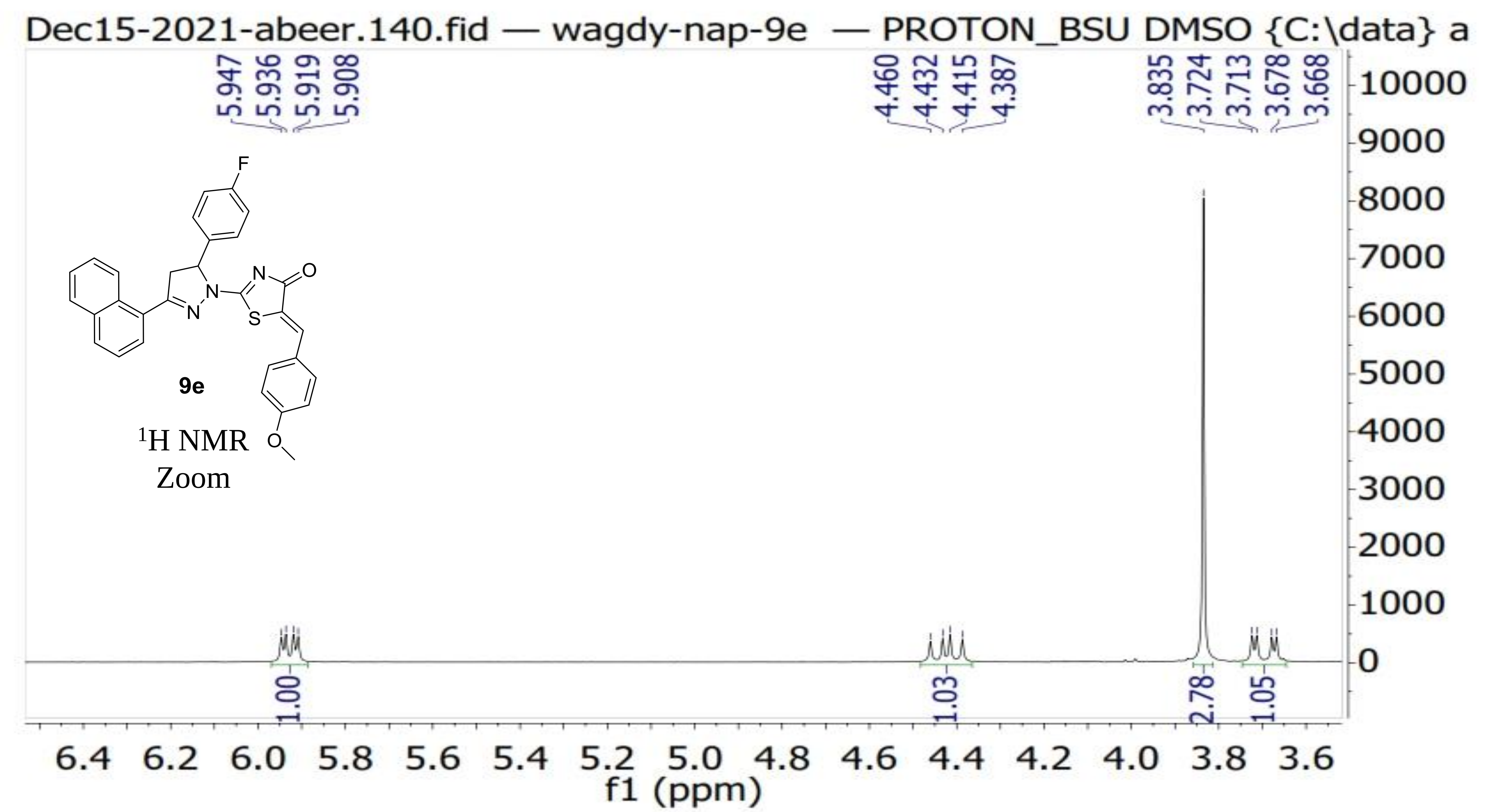

Dec15-2021-abeer.140.fid — wagdy-nap-9e — PROTON_BSU DMSO {C:\data} a
5.947
5.936
5.919
5.908
4.460
4.432
4.415
4.387
3.835
3.724
3.713
3.678
3.668
F
N
O
N
N
S
O
9e
^{1}H NMR
Zoom
1.00
1.03
2.78
1.05
10000
9000
8000
7000
6000
5000
4000
3000
2000
1000
0
6.4
6.2
6.0
5.8
5.6
5.4
5.2
5.0
4.8
4.6
4.4
4.2
4.0
3.8
3.6
f1 (ppm)

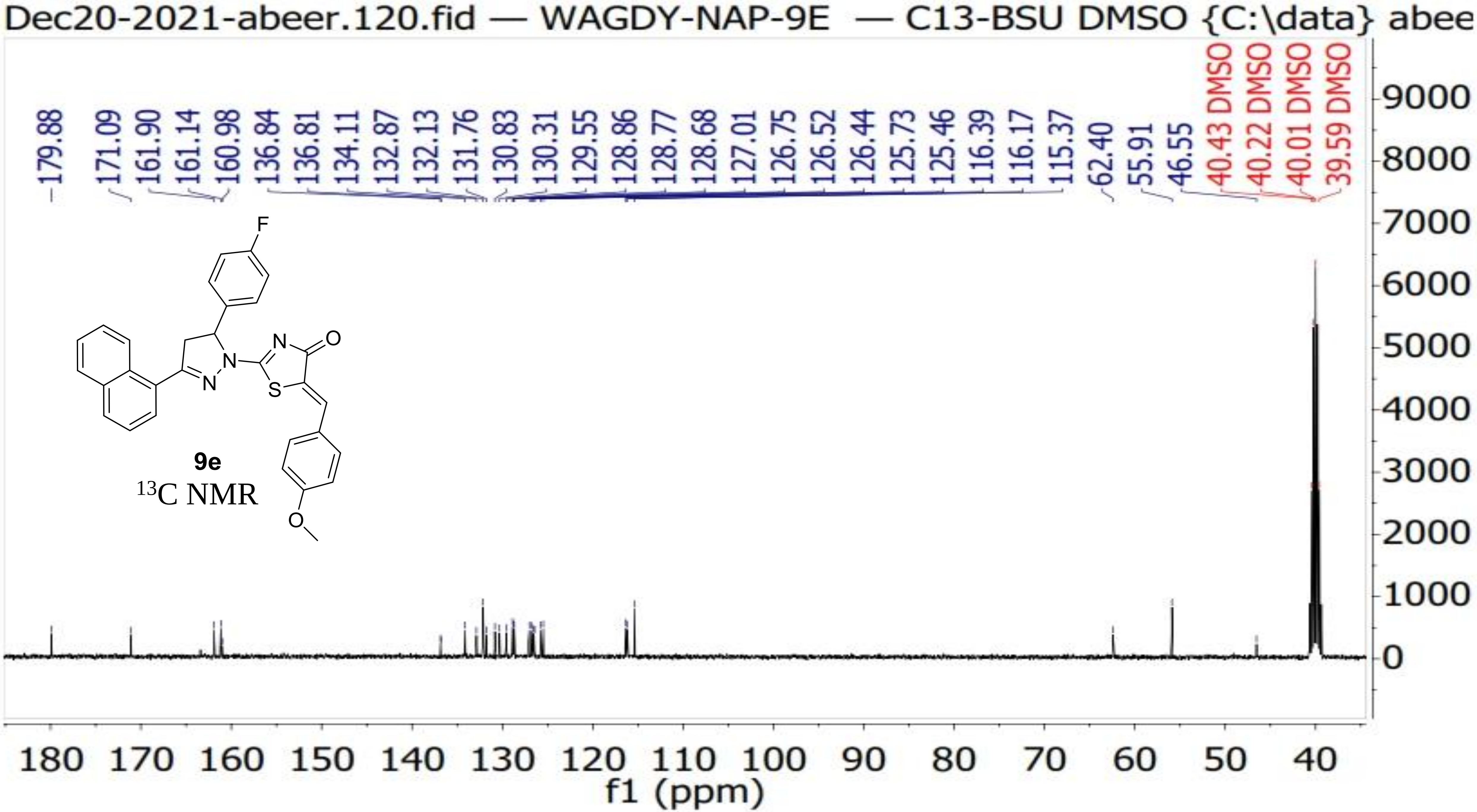
Dec20-2021-abeer.120.fid — WAGDY-NAP-9E — C13-BSU DMSO {C:\data} abee
179.88
171.09
161.90
161.14
160.98
136.84
136.81
134.11
132.87
132.13
131.76
130.83
130.31
129.55
128.86
128.77
128.68
127.01
126.75
126.52
126.44
125.73
125.46
116.39
116.17
115.37
62.40
55.91
46.55
40.43 DMSO
40.22 DMSO
40.01 DMSO
39.59 DMSO
F
N
O
N
N
S
O
9e
13C NMR
f1 (ppm)

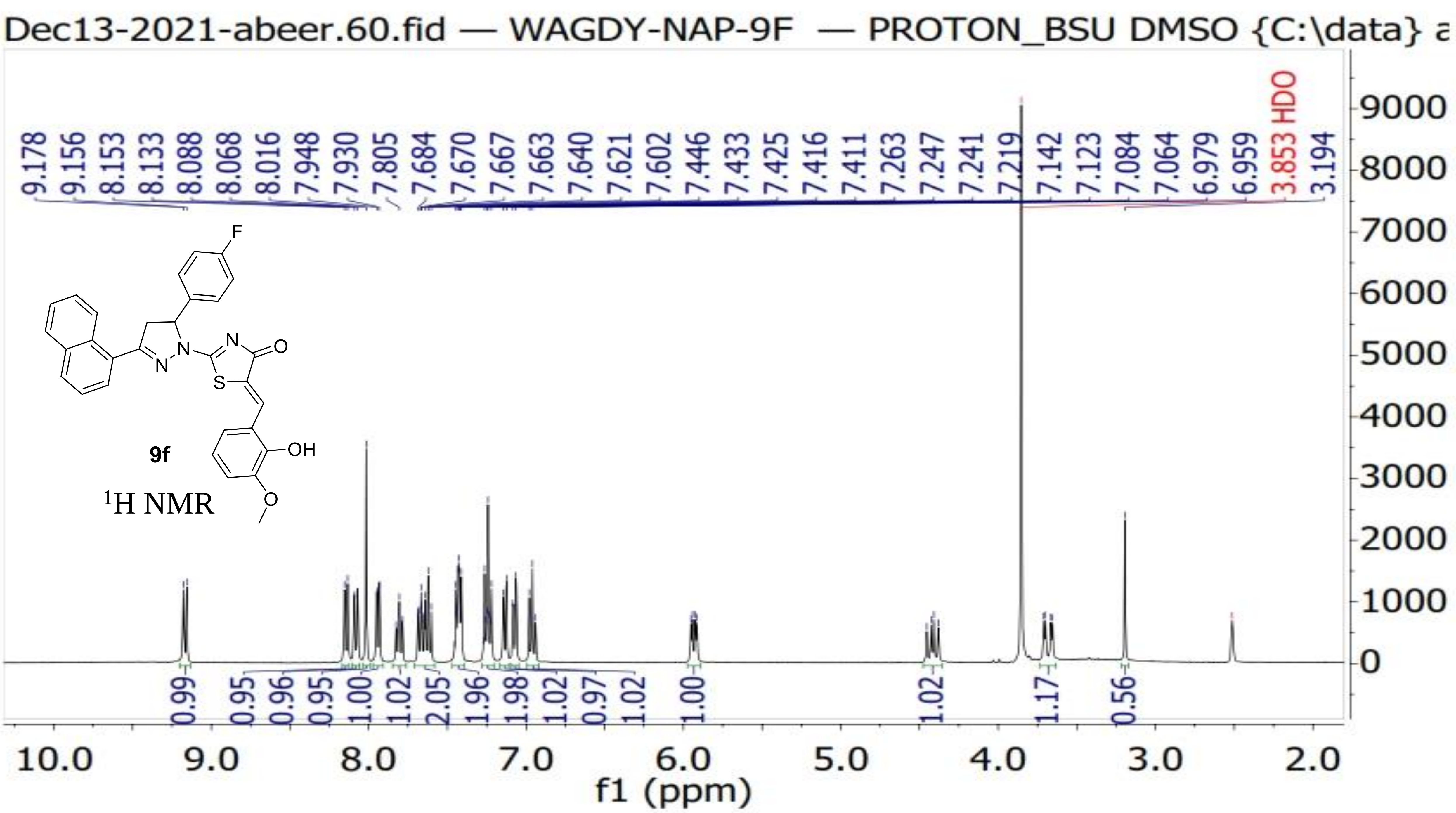

Dec13-2021-abeer.60.fid — WAGDY-NAP-9F — PROTON_BSU DMSO {C:\data} a
9.178 9.156 8.153 8.133 8.088 8.068 8.016 7.948 7.930 7.805 7.684 7.670 7.667 7.663 7.640 7.621 7.602 7.446 7.433 7.425 7.416 7.411 7.263 7.247 7.241 7.219 7.142 7.123 7.084 7.064 6.979 6.959 3.853 HDO 3.194
9000
8000
7000
6000
5000
4000
3000
2000
1000
0
0.99 0.95 0.96 0.95 1.00 1.02 2.05 1.96 1.98 1.02 0.97 1.02 1.00 1.02 1.17 0.56
10.0 9.0 8.0 7.0 6.0 5.0 4.0 3.0 2.0
f1 (ppm)
F
N
N
N
O
S
OH
O
9f
1H NMR

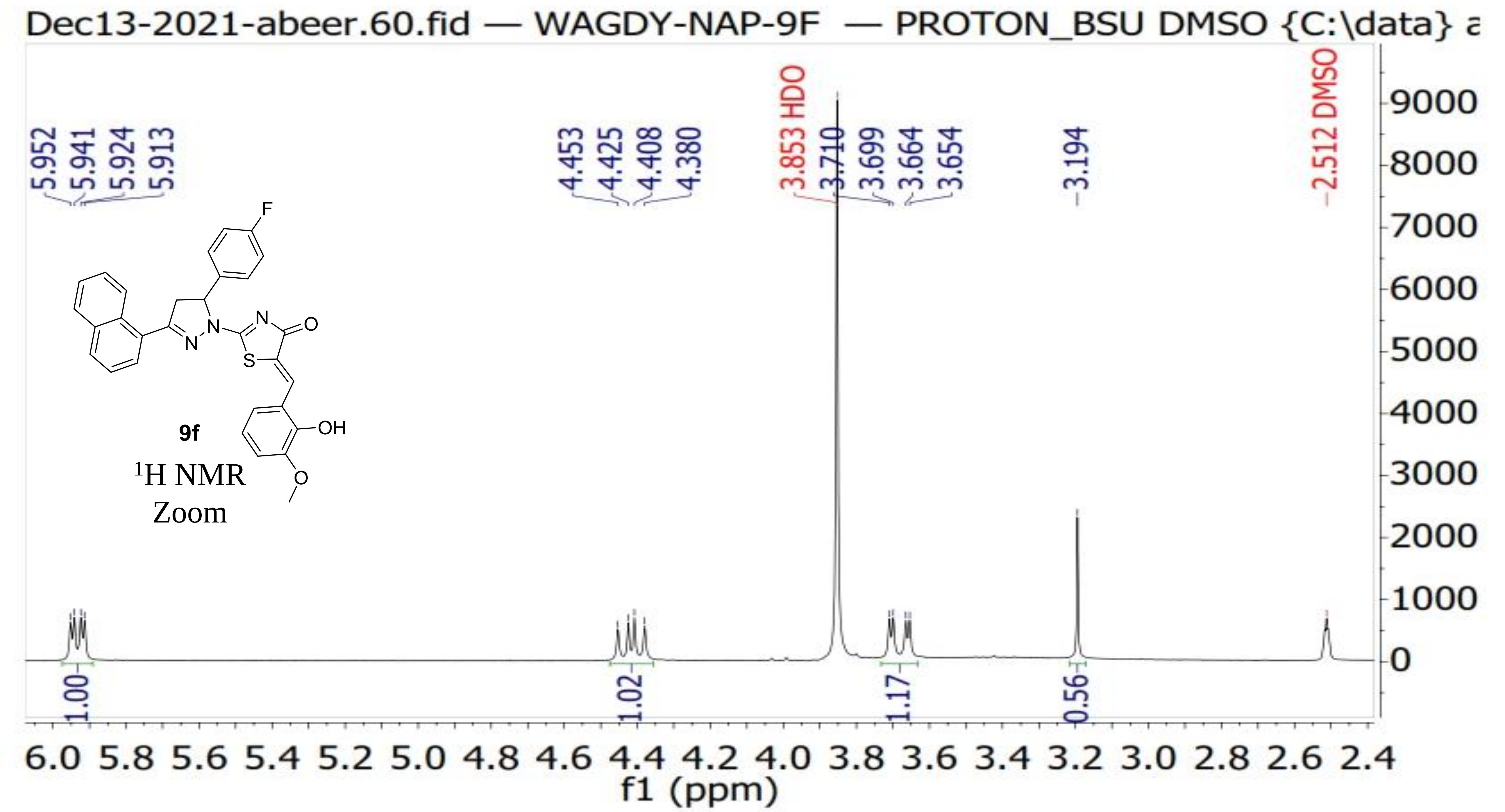
Dec13-2021-abeer.60.fid — WAGDY-NAP-9F — PROTON_BSU DMSO {C:\data} a
5.952
5.941
5.924
5.913
4.453
4.425
4.408
4.380
3.853 HDO
3.710
3.699
3.664
3.654
3.194
2.512 DMSO
F
N
N
N
O
S
OH
O
9f
1H NMR
Zoom
1.00
1.02
1.17
0.56
9000
8000
7000
6000
5000
4000
3000
2000
1000
0
6.0 5.8 5.6 5.4 5.2 5.0 4.8 4.6 4.4 4.2 4.0 3.8 3.6 3.4 3.2 3.0 2.8 2.6 2.4
f1 (ppm)

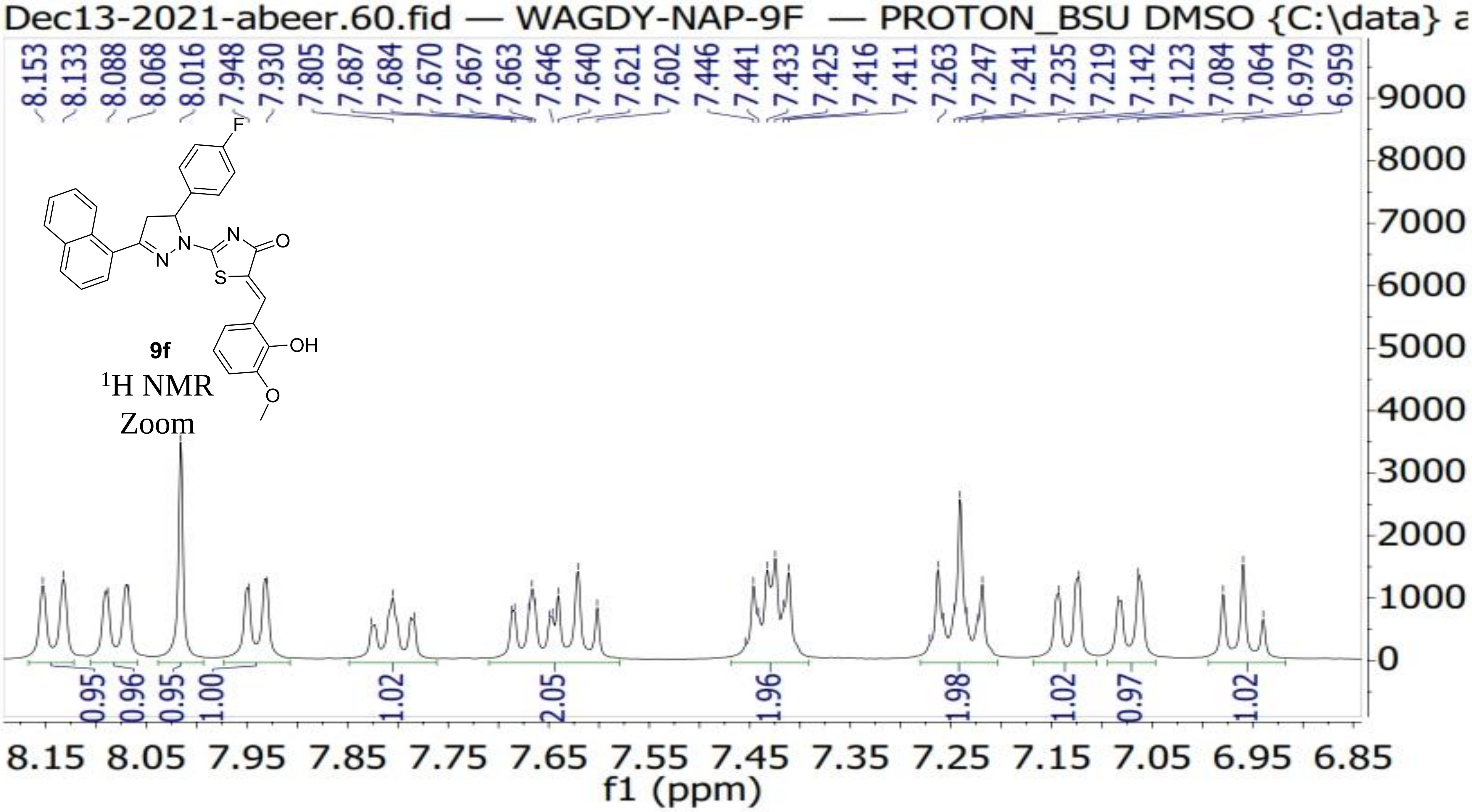

Dec13-2021-abeer.60.fid — WAGDY-NAP-9F — PROTON_BSU DMSO {C:\data} a
8.153 8.133 8.088 8.068 8.016 7.948 7.930 7.805 7.687 7.684 7.670 7.667 7.663 7.646 7.640 7.621 7.602 7.446 7.441 7.433 7.425 7.416 7.411 7.263 7.247 7.241 7.235 7.219 7.142 7.123 7.084 7.064 6.979 6.959
9f
^{1}H NMR
Zoom
0.95 0.96 0.95 1.00 1.02 2.05 1.96 1.98 1.02 0.97 1.02
f1 (ppm)

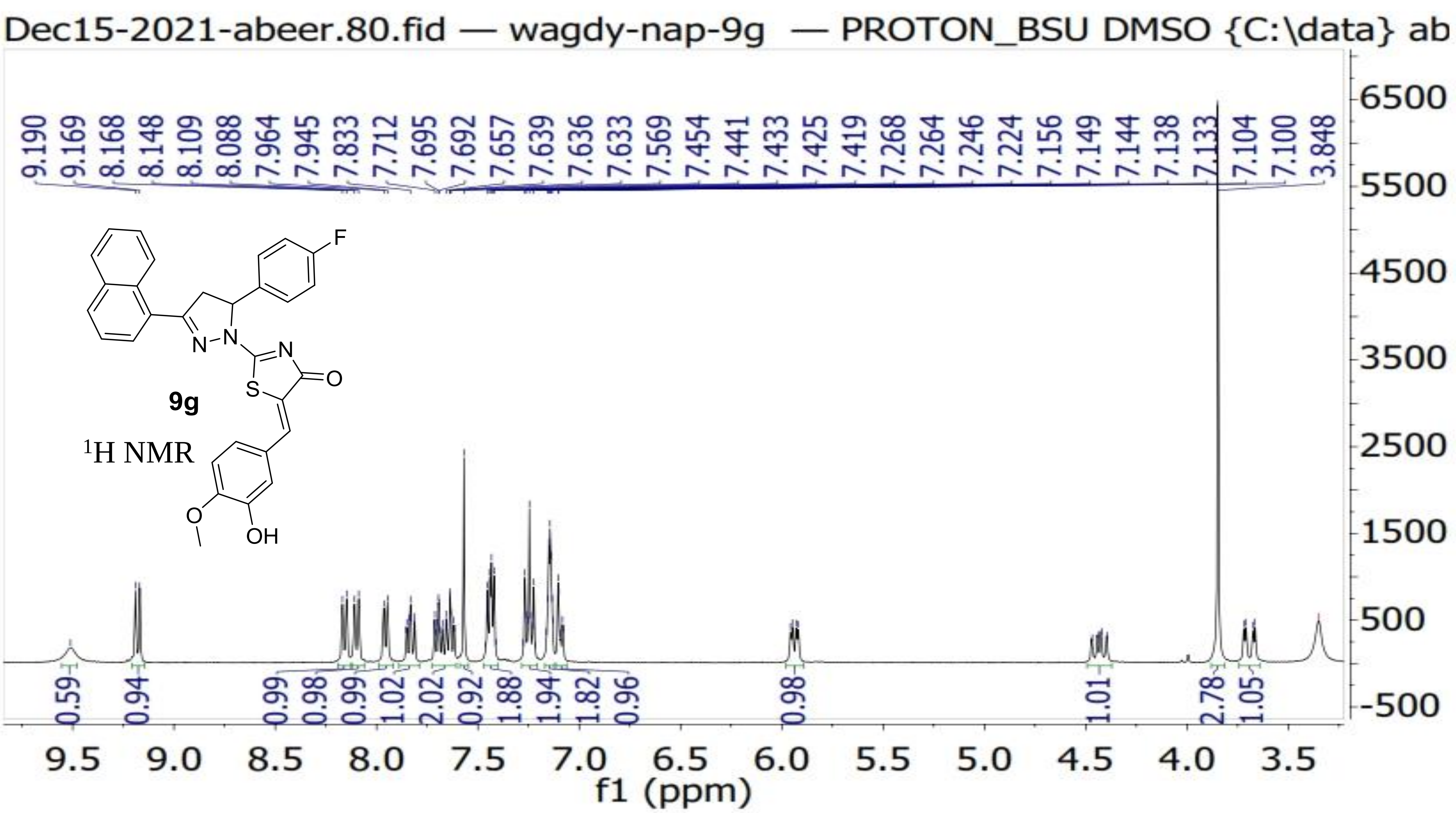
Dec15-2021-abeer.80.fid — wagdy-nap-9g — PROTON_BSU DMSO {C:\data} ab
9.190 9.169 8.168 8.148 8.109 8.088 7.964 7.945 7.833 7.712 7.695 7.692 7.657 7.639 7.636 7.633 7.569 7.454 7.441 7.433 7.425 7.419 7.268 7.264 7.246 7.224 7.156 7.149 7.144 7.138 7.133 7.104 7.100 3.848
F
N
N
N
S
O
9g
¹H NMR
O
OH
0.59 0.94 0.99 0.98 0.99 1.02 2.02 0.92 1.88 1.94 1.82 0.96 0.98 1.01 2.78 1.05
6500
5500
4500
3500
2500
1500
500
-500
9.5 9.0 8.5 8.0 7.5 7.0 6.5 6.0 5.5 5.0 4.5 4.0 3.5
f1 (ppm)

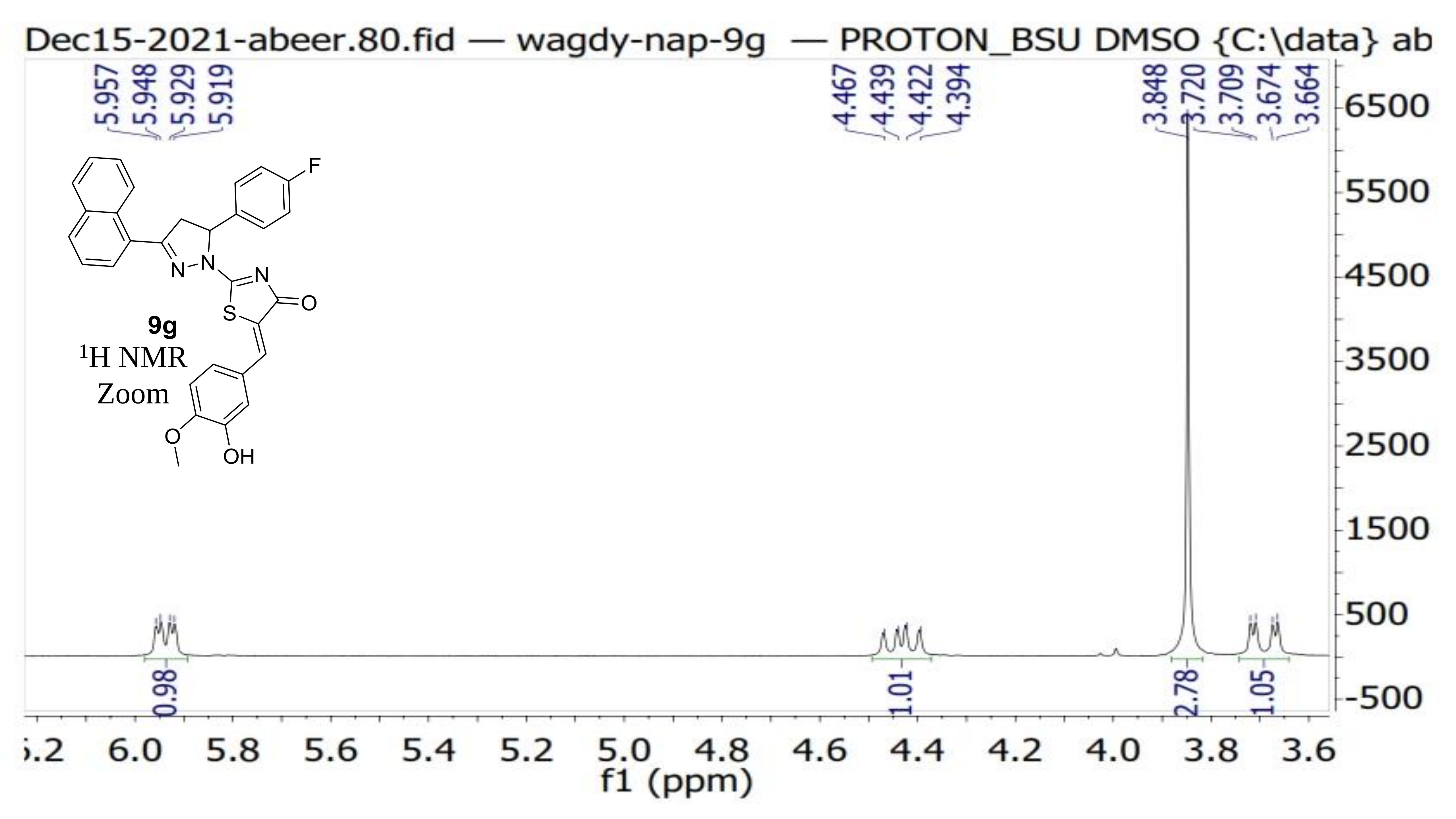
Dec15-2021-abeer.80.fid — wagdy-nap-9g — PROTON_BSU DMSO {C:\data} ab
5.957
5.948
5.929
5.919
4.467
4.439
4.422
4.394
3.848
3.720
3.709
3.674
3.664
F
N
N
N
S
O
O
OH
9g
1H NMR
Zoom
0.98
1.01
2.78
1.05
6500
5500
4500
3500
2500
1500
500
-500
6.0
5.8
5.6
5.4
5.2
5.0
4.8
4.6
4.4
4.2
4.0
3.8
3.6
f1 (ppm)

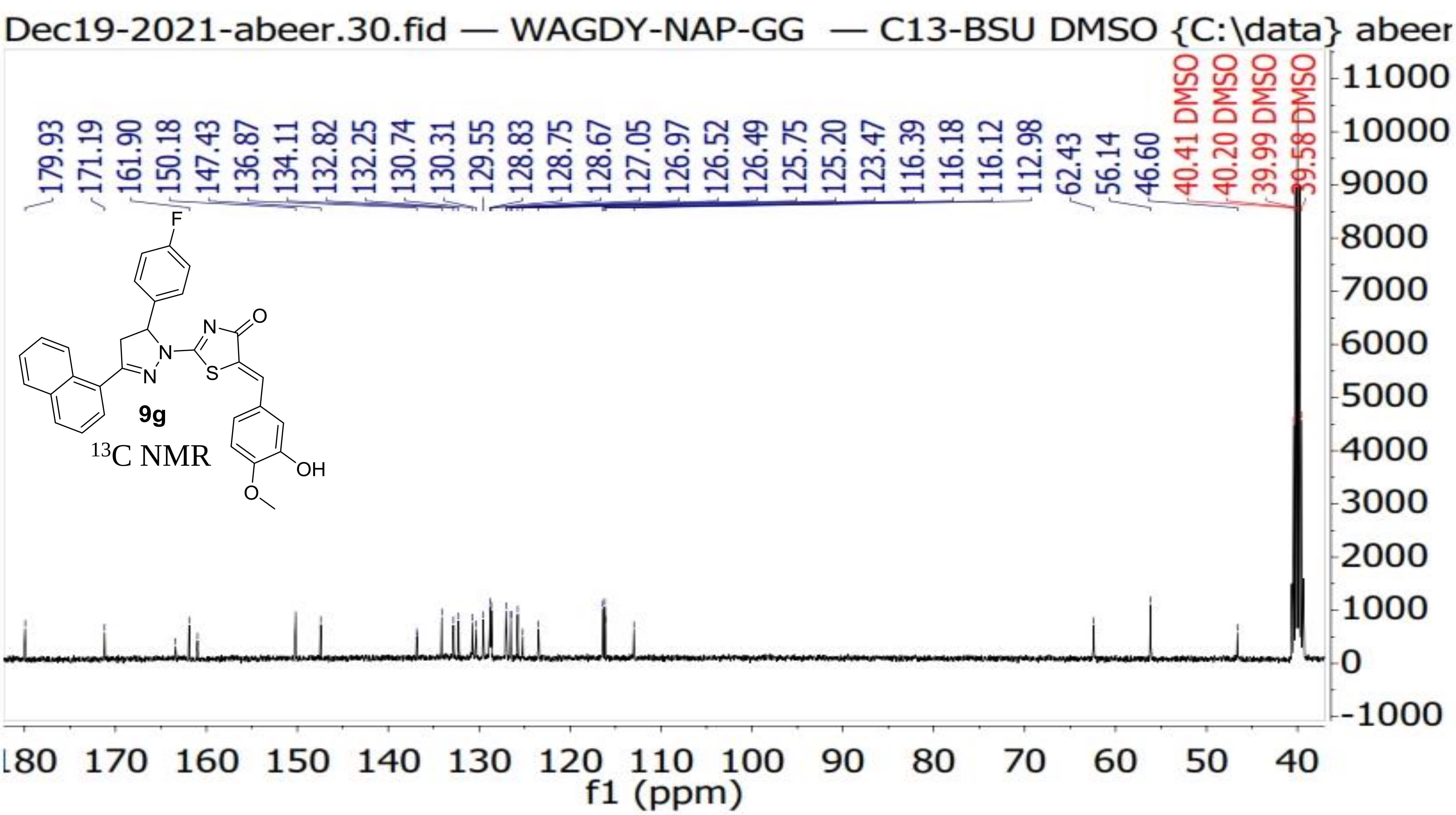

Dec19-2021-abeer.30.fid — WAGDY-NAP-GG — C13-BSU DMSO {C:\data} abeer
179.93
171.19
161.90
150.18
147.43
136.87
134.11
132.82
132.25
130.74
130.31
129.55
128.83
128.75
128.67
127.05
126.97
126.52
126.49
125.75
125.20
123.47
116.39
116.18
116.12
112.98
62.43
56.14
46.60
40.41 DMSO
40.20 DMSO
39.99 DMSO
39.58 DMSO
F
N
O
N
S
N
N
OH
O
9g
13C NMR
f1 (ppm)

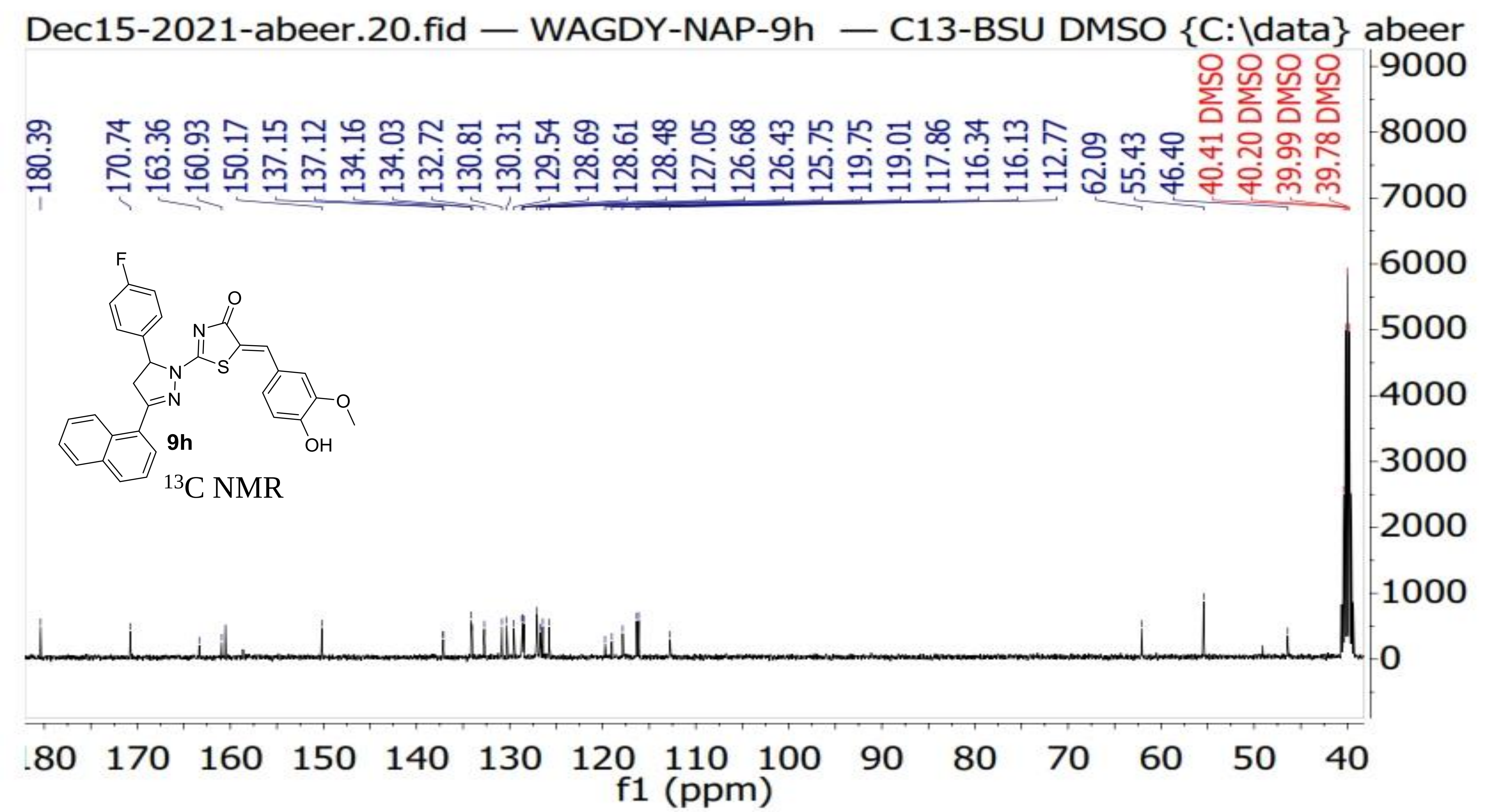

Dec15-2021-abeer.20.fid — WAGDY-NAP-9h — C13-BSU DMSO {C:\data} abeer
180.39
170.74
163.36
160.93
150.17
137.15
137.12
134.16
134.03
132.72
130.81
130.31
129.54
128.69
128.61
128.48
127.05
126.68
126.43
125.75
119.75
119.01
117.86
116.34
116.13
112.77
62.09
55.43
46.40
40.41 DMSO
40.20 DMSO
39.99 DMSO
39.78 DMSO
F
O
N
N
S
N
O
OH
9h
^{13}C NMR
f1 (ppm)

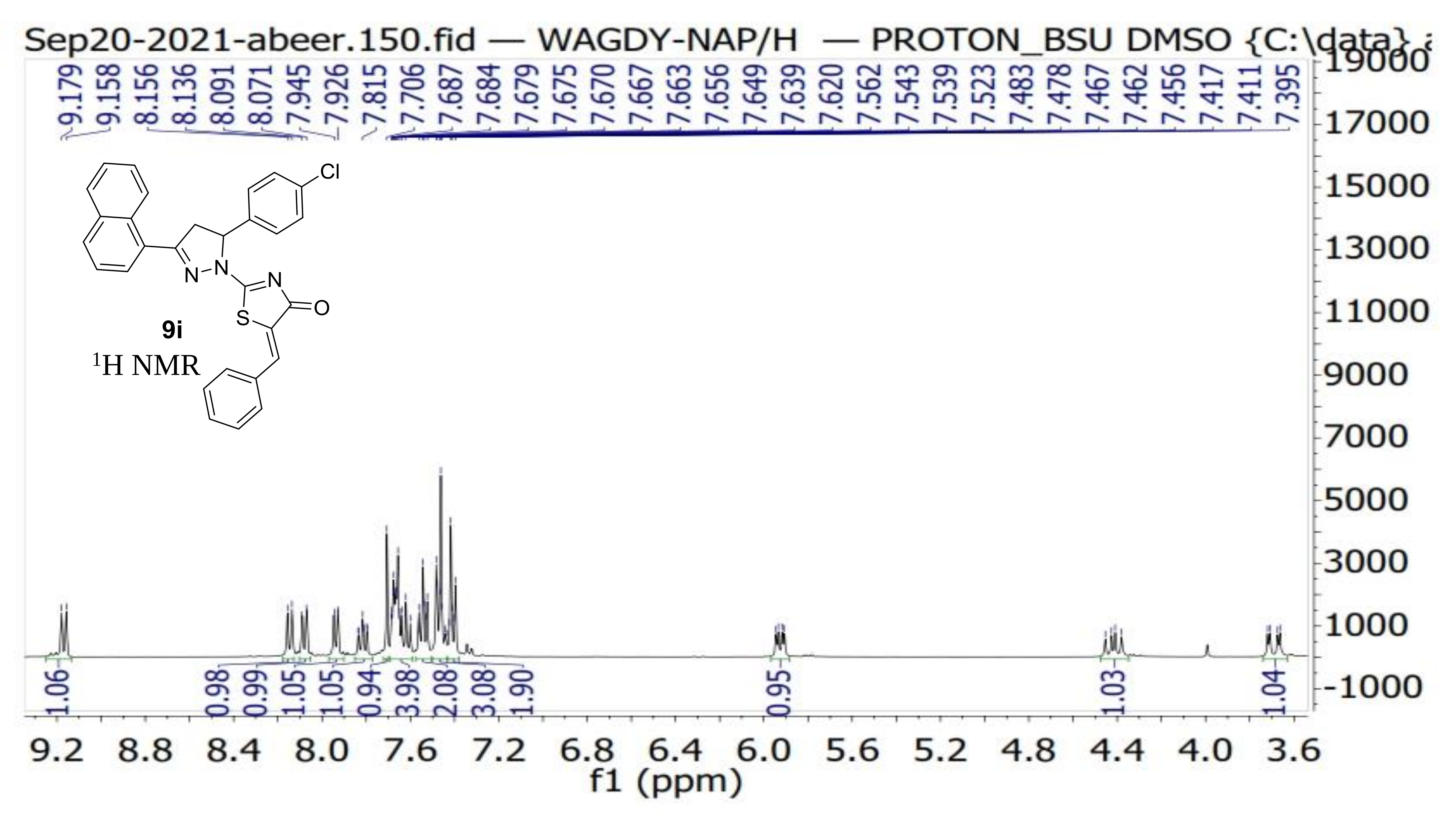

Sep20-2021-abeer.150.fid — WAGDY-NAP/H — PROTON_BSU DMSO {C:\data}
9.179 9.158 8.156 8.136 8.091 8.071 7.945 7.926 7.815 7.706 7.687 7.684 7.679 7.675 7.670 7.667 7.663 7.656 7.649 7.639 7.620 7.562 7.543 7.539 7.523 7.483 7.478 7.467 7.462 7.456 7.417 7.411 7.395
Cl
N
N
N
O
S
9i
1H NMR
1.06 0.98 0.99 1.05 1.05 0.94 3.98 2.08 3.08 1.90 0.95 1.03 1.04
f1 (ppm)

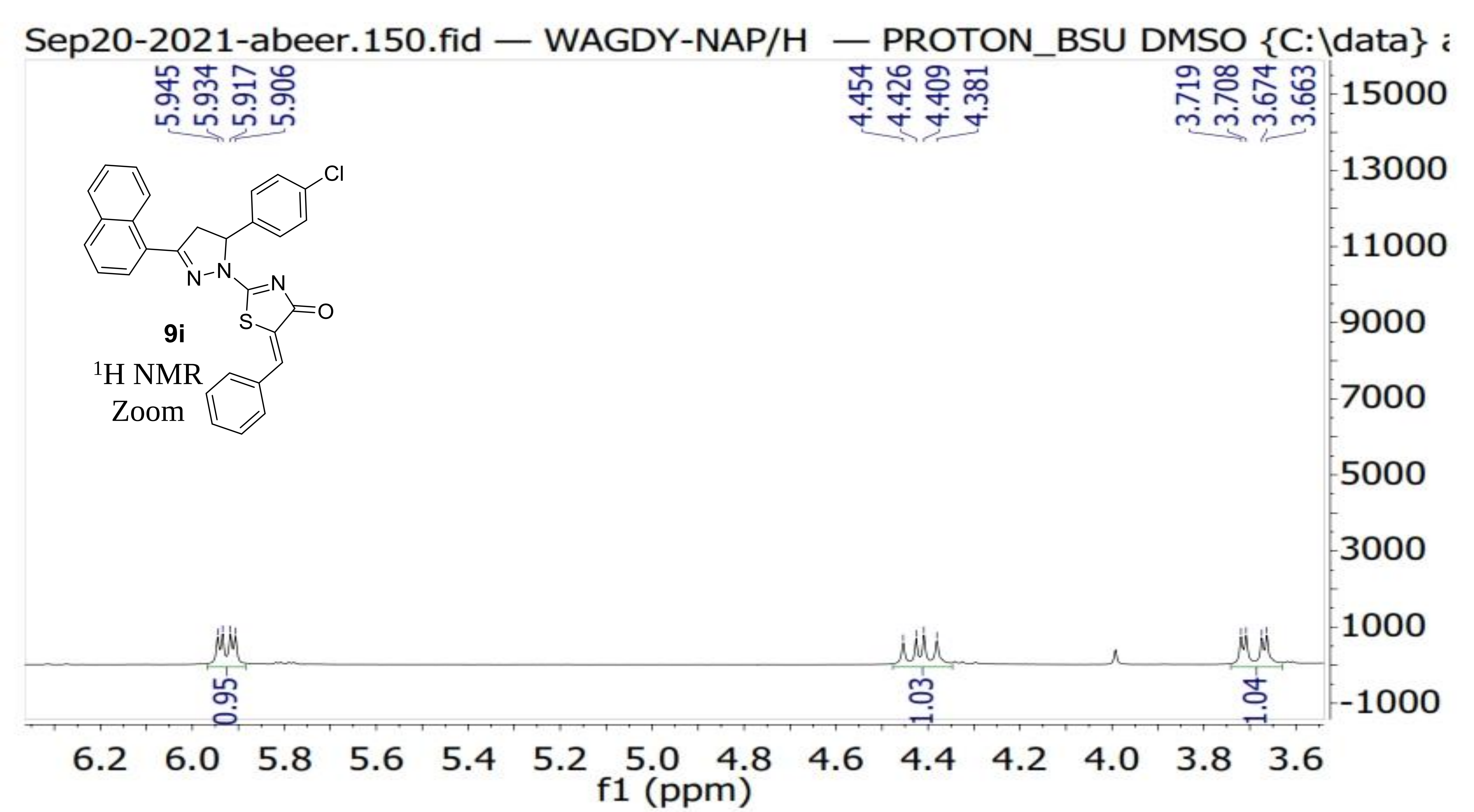
Sep20-2021-abeer.150.fid — WAGDY-NAP/H — PROTON_BSU DMSO {C:\data} a
5.945
5.934
5.917
5.906
4.454
4.426
4.409
4.381
3.719
3.708
3.674
3.663
Cl
N
N
N
O
S
9i
1H NMR
Zoom
0.95
1.03
1.04
15000
13000
11000
9000
7000
5000
3000
1000
-1000
6.2
6.0
5.8
5.6
5.4
5.2
5.0
4.8
4.6
4.4
4.2
4.0
3.8
3.6
f1 (ppm)

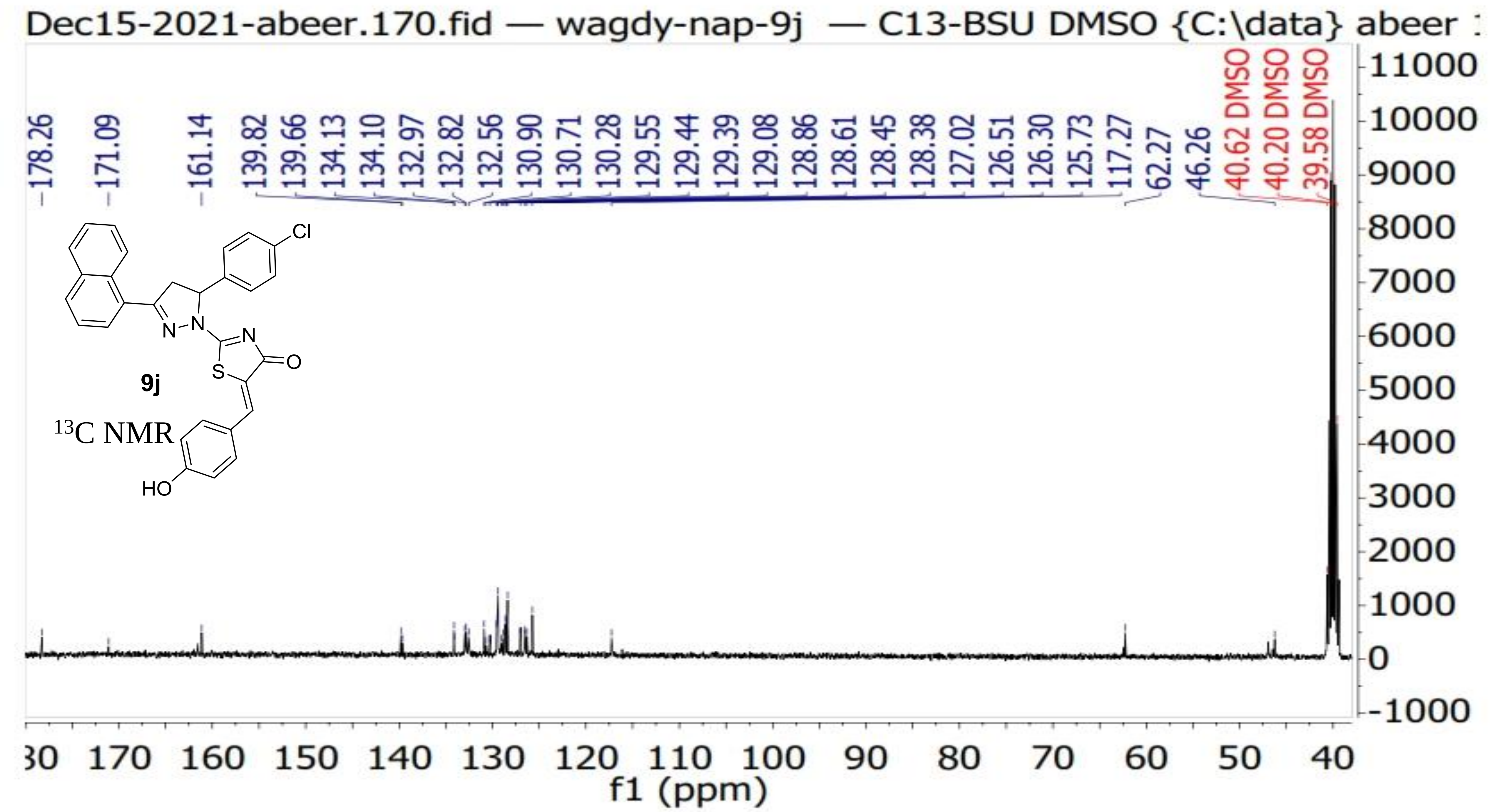
Dec15-2021-abeer.170.fid — wagdy-nap-9j — C13-BSU DMSO {C:\data} abeer
178.26
171.09
161.14
139.82
139.66
134.13
134.10
132.97
132.82
132.56
130.90
130.71
130.28
129.55
129.44
129.39
129.08
128.86
128.61
128.45
128.38
127.02
126.51
126.30
125.73
117.27
62.27
46.26
40.62 DMSO
40.20 DMSO
39.58 DMSO
Cl
N
N
N
S
O
HO
9j
13C NMR
f1 (ppm)

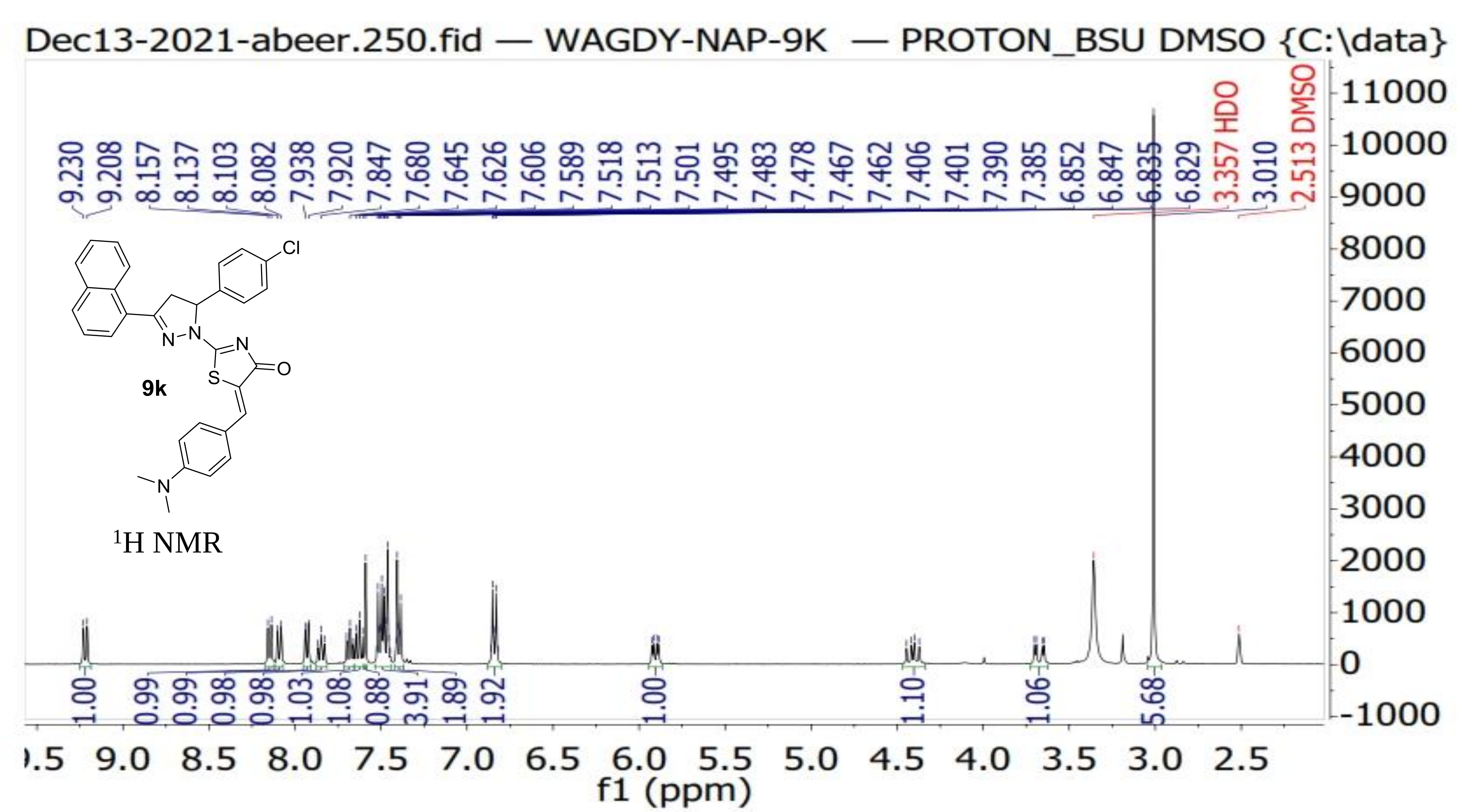
Dec13-2021-abeer.250.fid — WAGDY-NAP-9K — PROTON_BSU DMSO {C:\data}
9.230
9.208
8.157
8.137
8.103
8.082
7.938
7.920
7.847
7.680
7.645
7.626
7.606
7.589
7.518
7.513
7.501
7.495
7.483
7.478
7.467
7.462
7.406
7.401
7.390
7.385
6.852
6.847
6.835
6.829
3.357 HDO
3.010
2.513 DMSO
Cl
N
N
N
S
O
N
9k
1H NMR
1.00
0.99
0.99
0.98
0.98
1.03
1.08
0.88
3.91
1.89
1.92
1.00
1.10
1.06
5.68
f1 (ppm)

Dec13-2021-abeer.250.fid — WAGDY-NAP-9K — PROTON_BSU DMSO {C:\data}

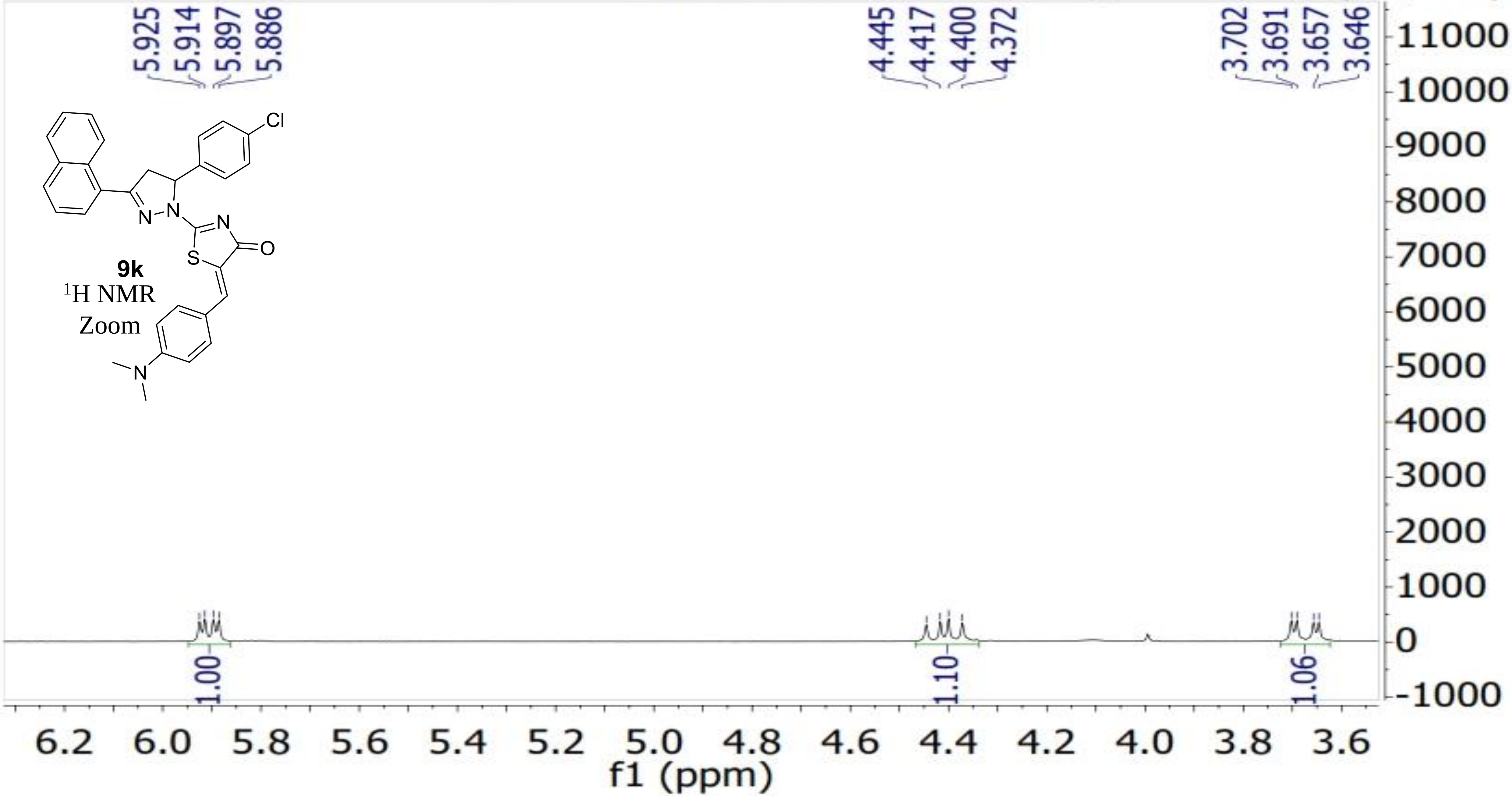

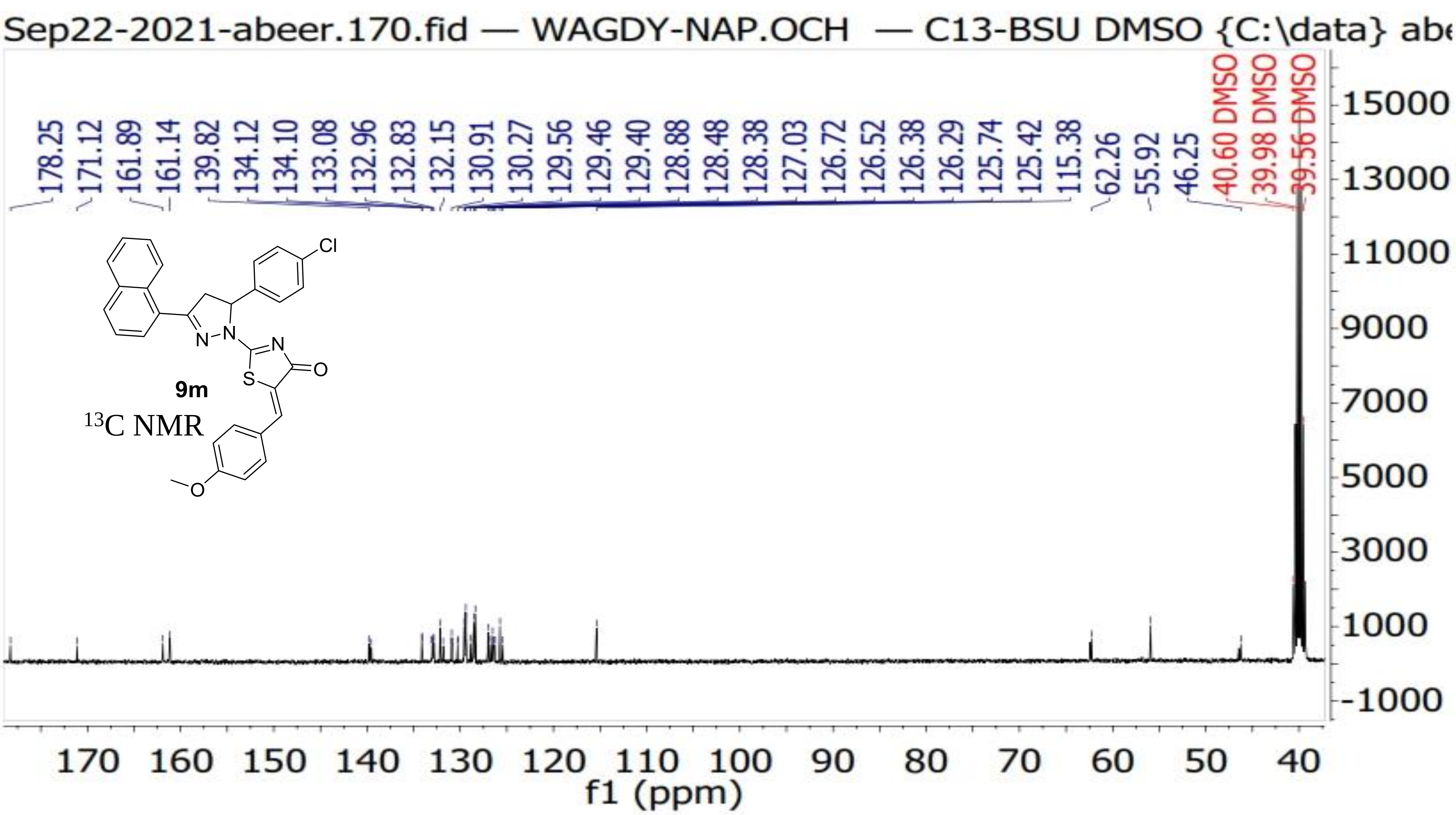
Sep22-2021-abeer.170.fid — WAGDY-NAP.OCH — C13-BSU DMSO {C:\data} abe
178.25
171.12
161.89
161.14
139.82
134.12
134.10
133.08
132.96
132.83
132.15
130.91
130.27
129.56
129.46
129.40
128.88
128.48
128.38
127.03
126.72
126.52
126.38
126.29
125.74
125.42
115.38
62.26
55.92
46.25
40.60 DMSO
39.98 DMSO
39.56 DMSO
Cl
N
N
N
S
O
O
9m
13C NMR
15000
13000
11000
9000
7000
5000
3000
1000
-1000
170
160
150
140
130
120
110
100
90
80
70
60
50
40
f1 (ppm)

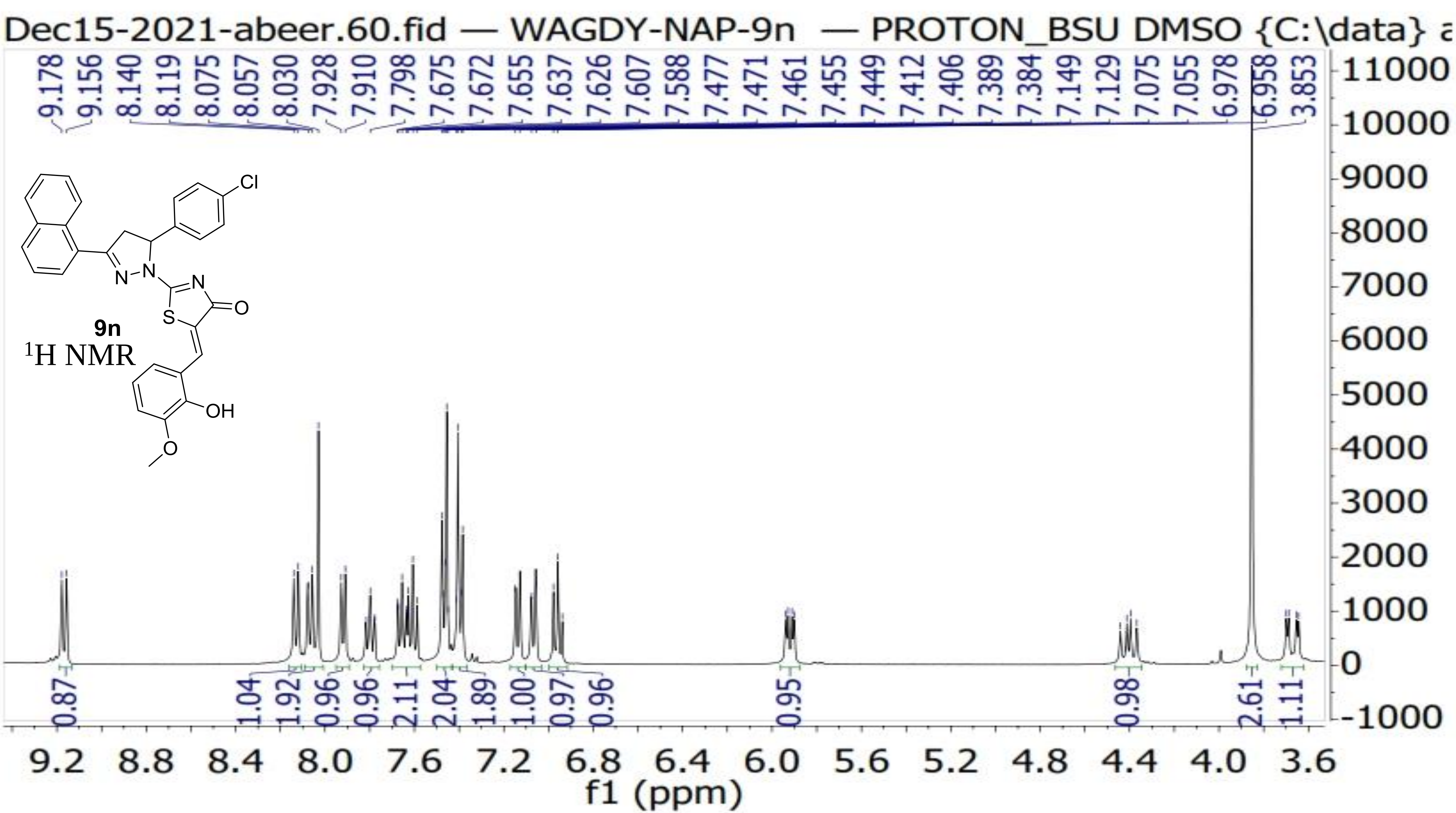

Dec15-2021-abeer.60.fid — WAGDY-NAP-9n — PROTON_BSU DMSO {C:\data} a
9.178 9.156 8.140 8.119 8.075 8.057 8.030 7.928 7.910 7.798 7.675 7.672 7.655 7.637 7.626 7.607 7.588 7.477 7.471 7.461 7.455 7.449 7.412 7.406 7.389 7.384 7.149 7.129 7.075 7.055 6.978 6.958 3.853
Cl
N
N
N
O
S
OH
O
9n
1H NMR
0.87 1.04 1.92 0.96 0.96 2.11 2.04 1.89 1.00 0.97 0.96 0.95 0.98 2.61 1.11
9.2 8.8 8.4 8.0 7.6 7.2 6.8 6.4 6.0 5.6 5.2 4.8 4.4 4.0 3.6
f1 (ppm)
11000 10000 9000 8000 7000 6000 5000 4000 3000 2000 1000 0 -1000

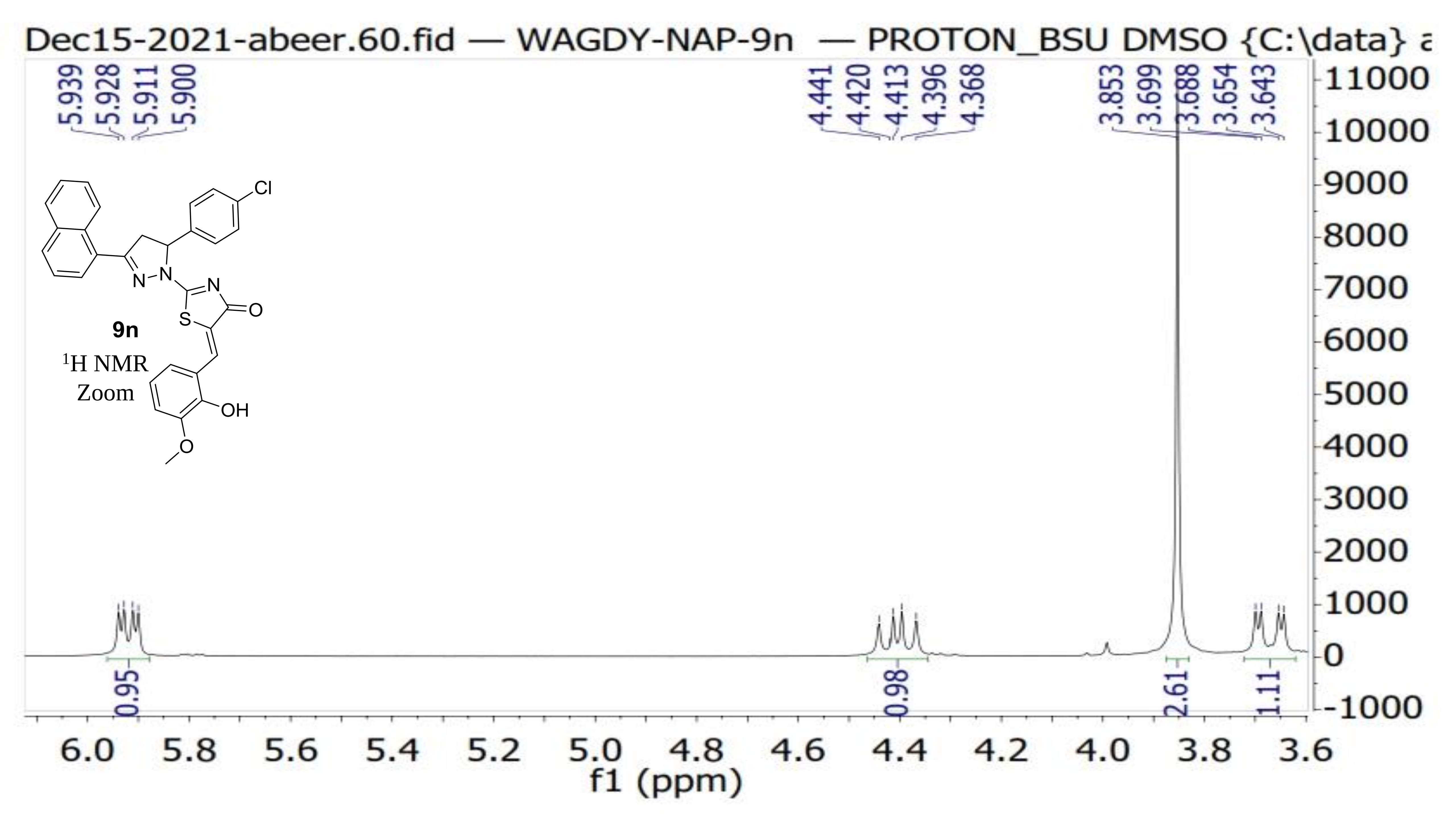

Dec15-2021-abeer.60.fid — WAGDY-NAP-9n — PROTON_BSU DMSO {C:\data} a
5.939
5.928
5.911
5.900
4.441
4.420
4.413
4.396
4.368
3.853
3.699
3.688
3.654
3.643
Cl
N
N
N
O
S
OH
O
9n
1H NMR
Zoom
0.95
0.98
2.61
1.11
11000
10000
9000
8000
7000
6000
5000
4000
3000
2000
1000
0
-1000
6.0
5.8
5.6
5.4
5.2
5.0
4.8
4.6
4.4
4.2
4.0
3.8
3.6
f1 (ppm)

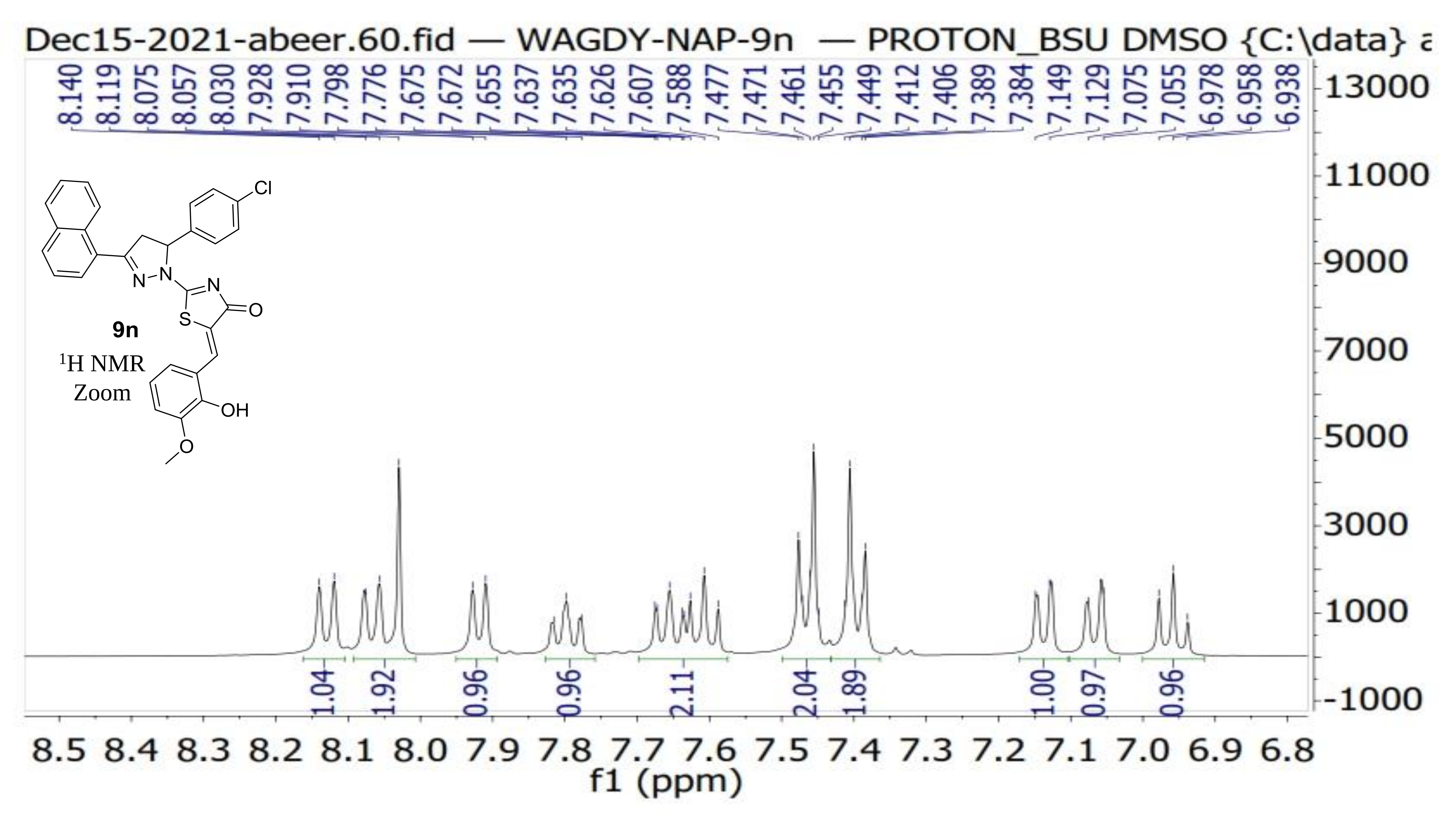
Dec15-2021-abeer.60.fid — WAGDY-NAP-9n — PROTON_BSU DMSO {C:\data} a
8.140 8.119 8.075 8.057 8.030 7.928 7.910 7.798 7.776 7.675 7.672 7.655 7.637 7.635 7.626 7.607 7.588 7.477 7.471 7.461 7.455 7.449 7.412 7.406 7.389 7.384 7.149 7.129 7.075 7.055 6.978 6.958 6.938
Cl
N
N
N
O
S
OH
O
9n
1H NMR
Zoom
1.04 1.92 0.96 0.96 2.11 2.04 1.89 1.00 0.97 0.96
13000 11000 9000 7000 5000 3000 1000 -1000
8.5 8.4 8.3 8.2 8.1 8.0 7.9 7.8 7.7 7.6 7.5 7.4 7.3 7.2 7.1 7.0 6.9 6.8
f1 (ppm)

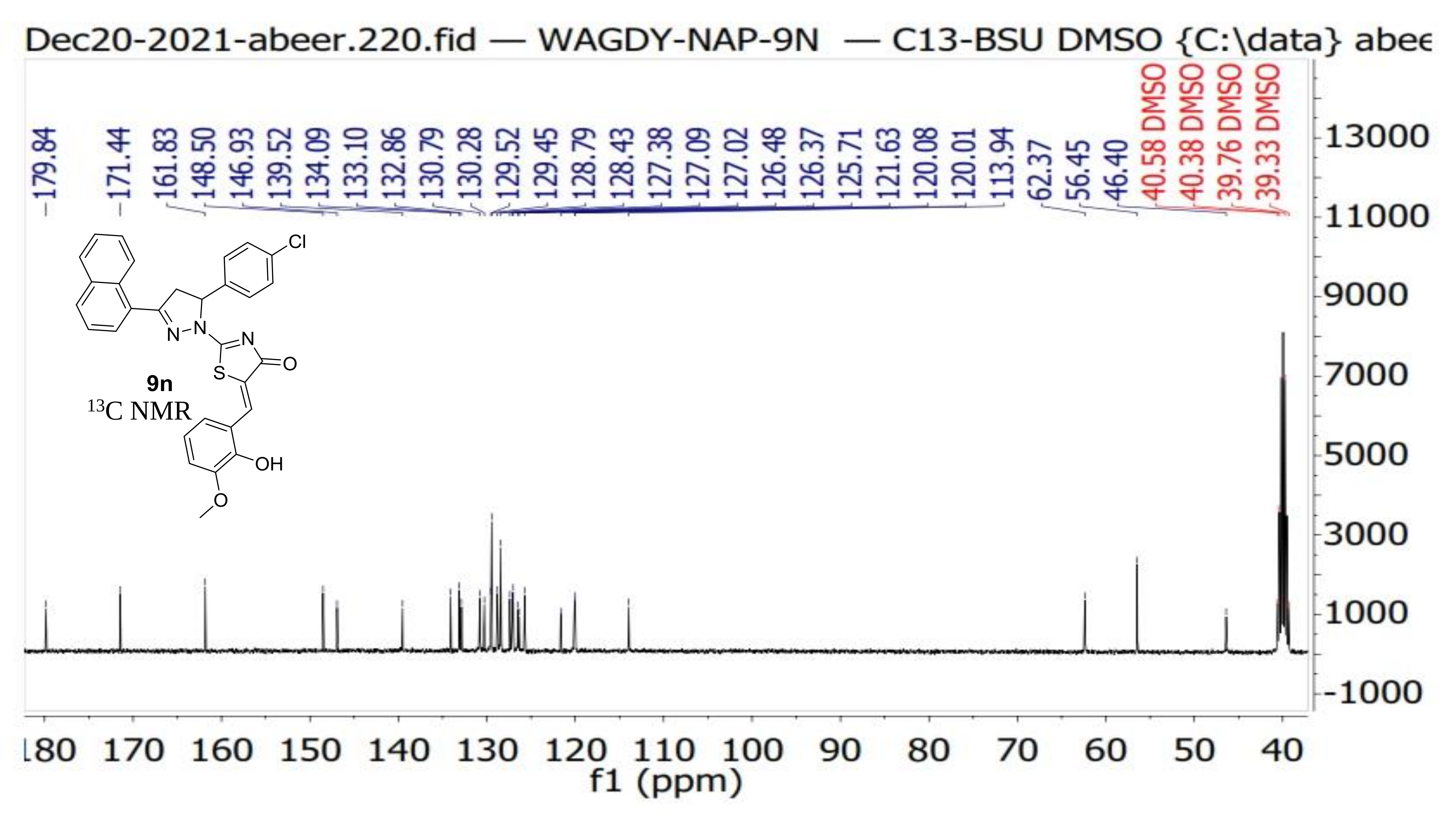

Dec20-2021-abeer.220.fid — WAGDY-NAP-9N — C13-BSU DMSO {C:\data} abee
179.84
171.44
161.83
148.50
146.93
139.52
134.09
133.10
132.86
130.79
130.28
129.52
129.45
128.79
128.43
127.38
127.09
127.02
126.48
126.37
125.71
121.63
120.08
120.01
113.94
62.37
56.45
46.40
40.58 DMSO
40.38 DMSO
39.76 DMSO
39.33 DMSO
Cl
N
N
N
O
S
OH
O
9n
13C NMR
13000
11000
9000
7000
5000
3000
1000
-1000
180 170 160 150 140 130 120 110 100 90 80 70 60 50 40
f1 (ppm)

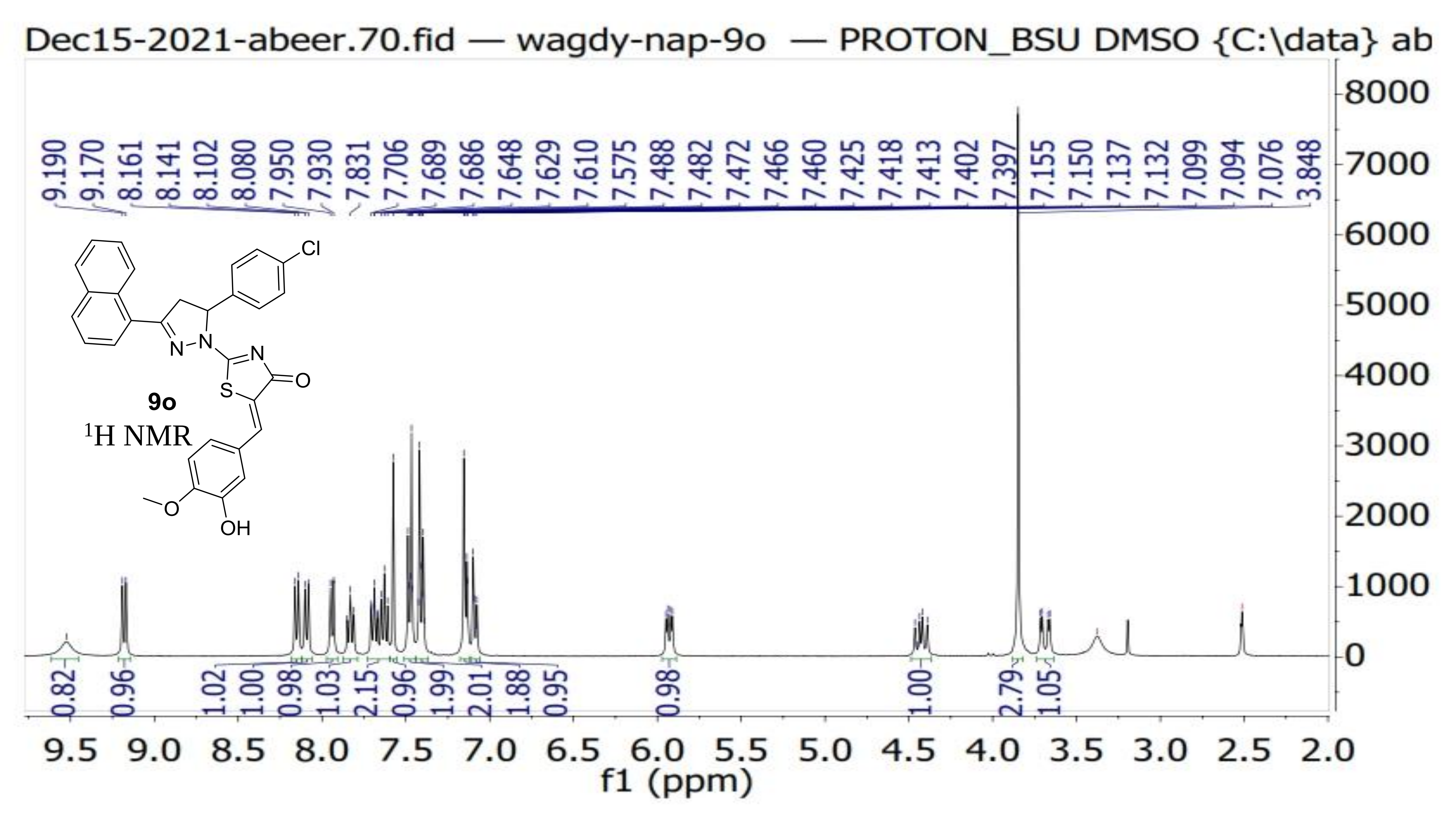
Dec15-2021-abeer.70.fid — wagdy-nap-9o — PROTON_BSU DMSO {C:\data} ab
9.190 9.170 8.161 8.141 8.102 8.080 7.950 7.930 7.831 7.706 7.689 7.686 7.648 7.629 7.610 7.575 7.488 7.482 7.472 7.466 7.460 7.425 7.418 7.413 7.402 7.397 7.155 7.150 7.137 7.132 7.099 7.094 7.076 3.848
Cl
N
N
N
O
S
O
OH
9o
1H NMR
0.82 0.96 1.02 1.00 0.98 1.03 2.15 0.96 1.99 2.01 1.88 0.95 0.98 1.00 2.79 1.05
8000
7000
6000
5000
4000
3000
2000
1000
0
9.5 9.0 8.5 8.0 7.5 7.0 6.5 6.0 5.5 5.0 4.5 4.0 3.5 3.0 2.5 2.0
f1 (ppm)

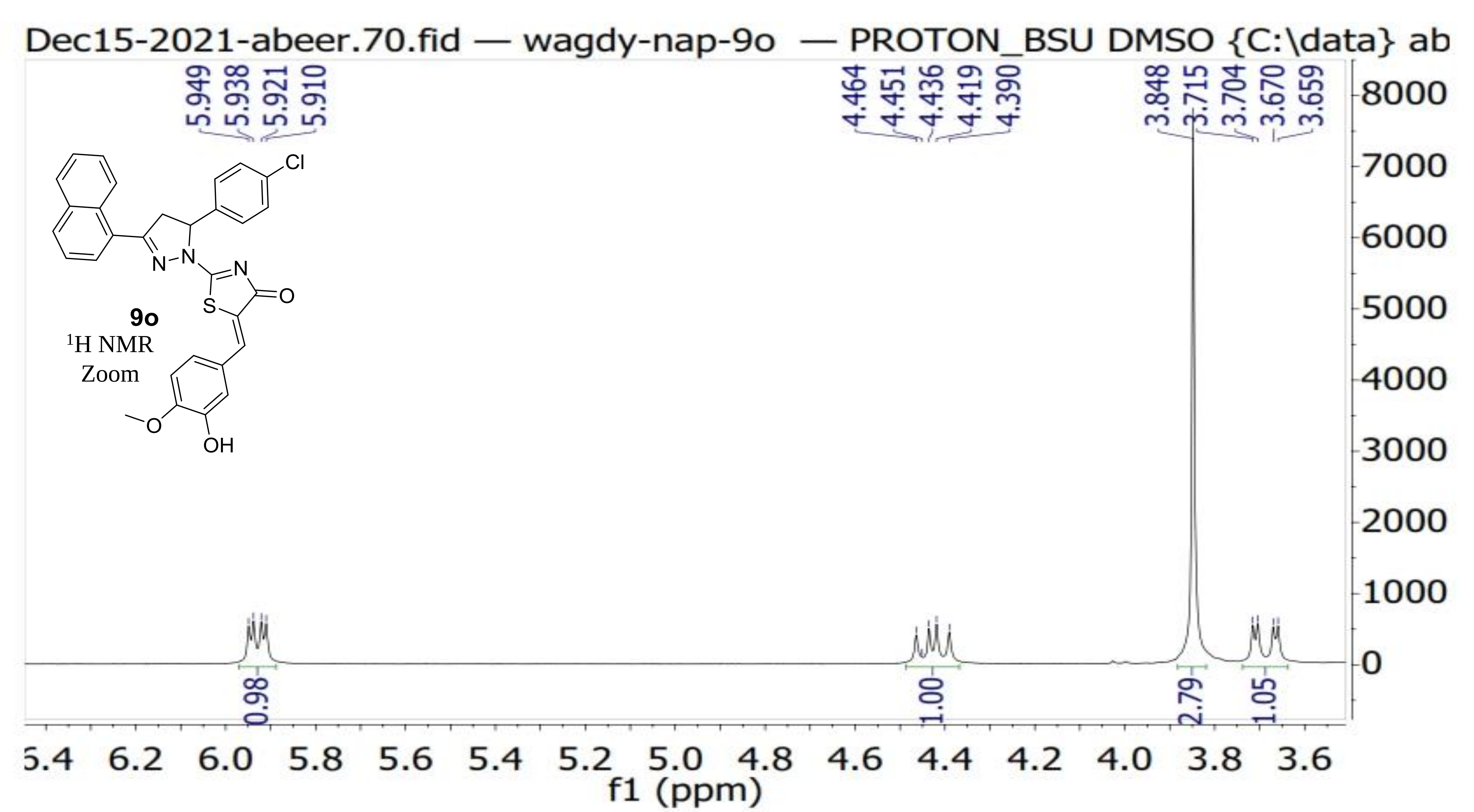

Dec15-2021-abeer.70.fid — wagdy-nap-9o — PROTON_BSU DMSO {C:\data} ab
5.949
5.938
5.921
5.910
4.464
4.451
4.436
4.419
4.390
3.848
3.715
3.704
3.670
3.659
Cl
N
N
N
O
S
OH
O
9o
¹H NMR
Zoom
0.98
1.00
2.79
1.05
f1 (ppm)

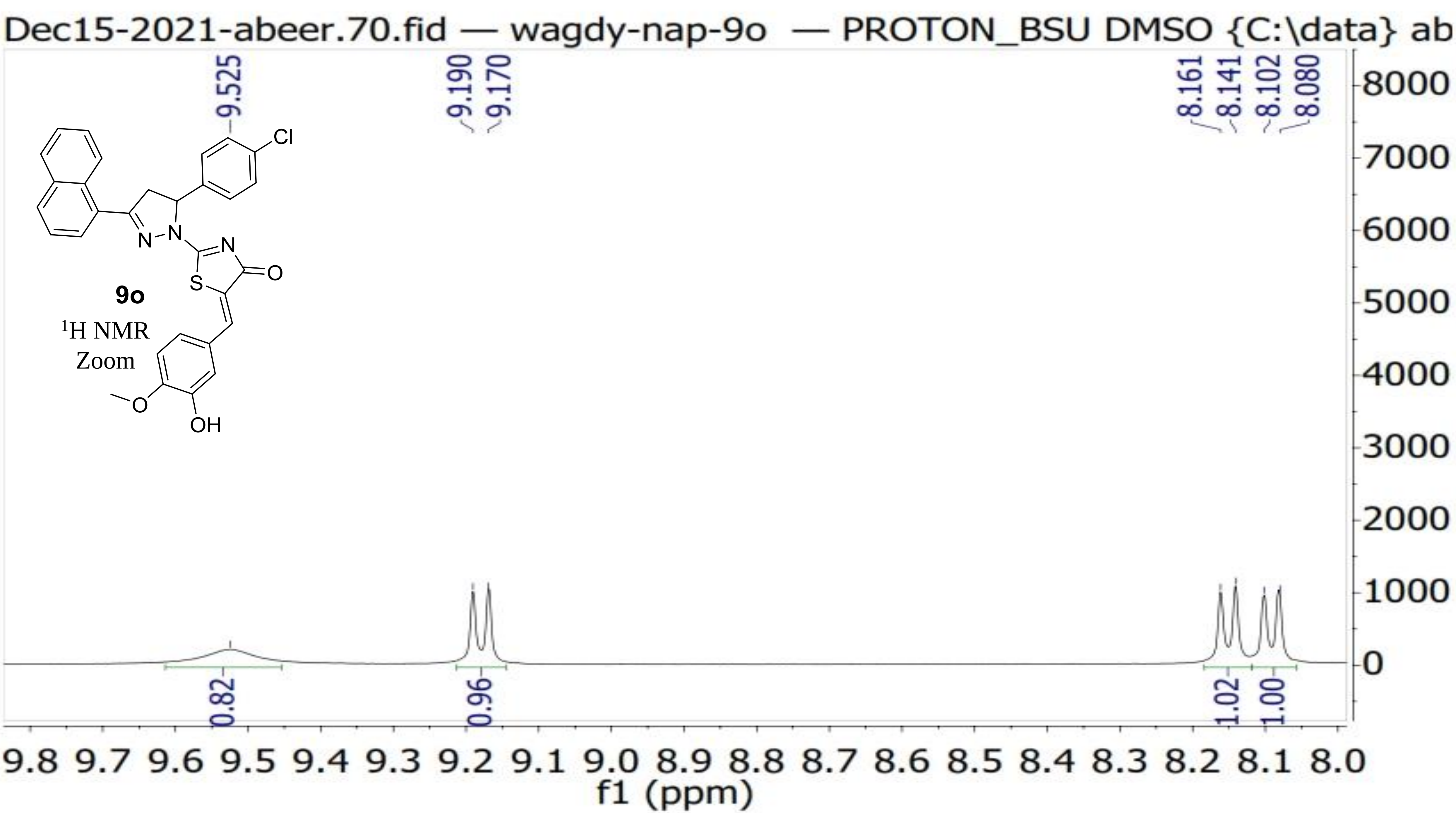
Dec15-2021-abeer.70.fid — wagdy-nap-9o — PROTON_BSU DMSO {C:\data} ab
9.525
9.190
9.170
8.161
8.141
8.102
8.080
Cl
N
N
N
S
O
O
OH
9o
¹H NMR
Zoom
0.82
0.96
1.02
1.00
8000
7000
6000
5000
4000
3000
2000
1000
0
9.8 9.7 9.6 9.5 9.4 9.3 9.2 9.1 9.0 8.9 8.8 8.7 8.6 8.5 8.4 8.3 8.2 8.1 8.0
f1 (ppm)

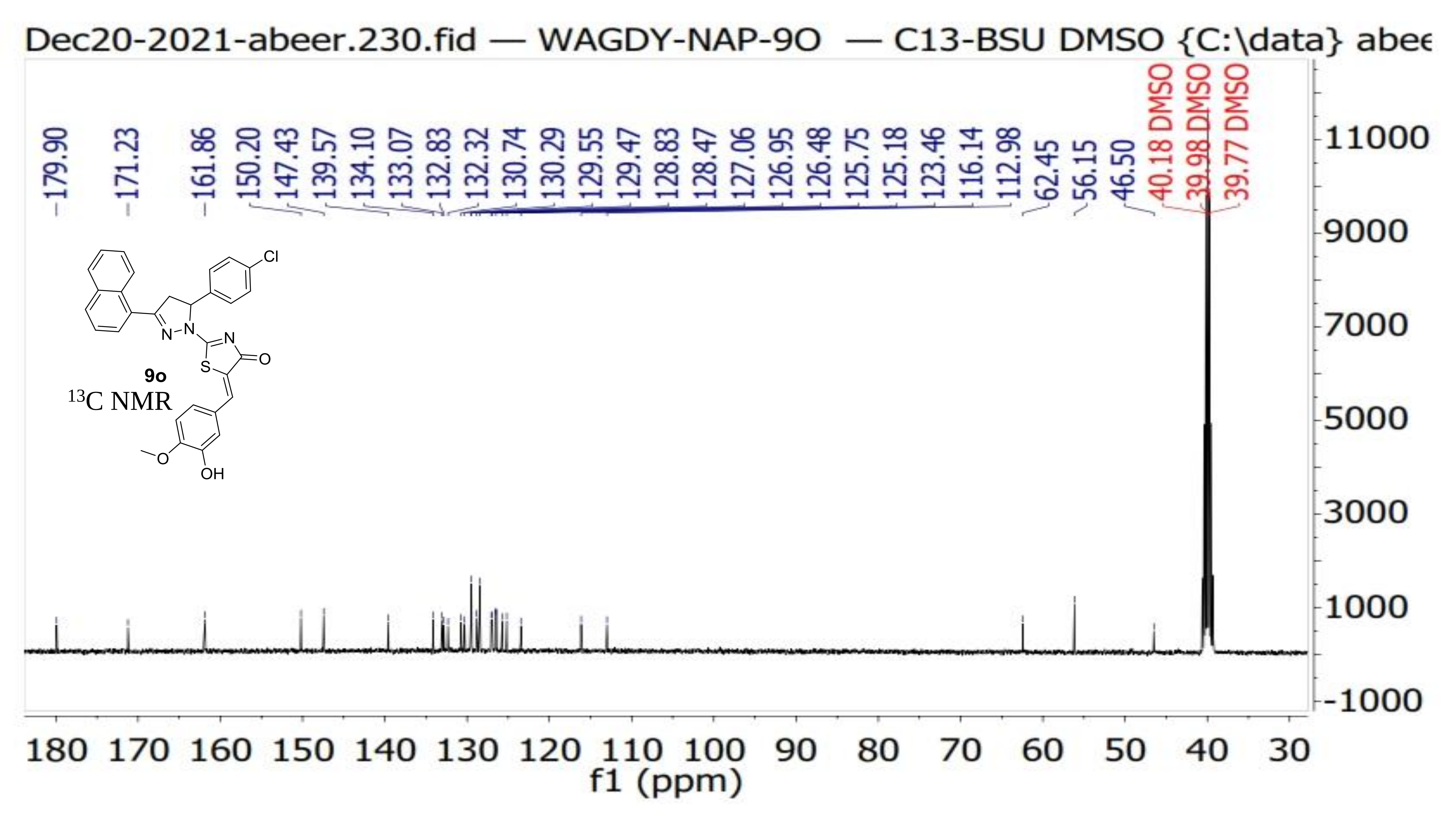

Dec20-2021-abeer.230.fid — WAGDY-NAP-9O — C13-BSU DMSO {C:\data} abee
179.90
171.23
161.86
150.20
147.43
139.57
134.10
133.07
132.83
132.32
130.74
130.29
129.55
129.47
128.83
128.47
127.06
126.95
126.48
125.75
125.18
123.46
116.14
112.98
62.45
56.15
46.50
40.18 DMSO
39.98 DMSO
39.77 DMSO
Cl
N
N
N
S
O
O
OH
9o
13C NMR
11000
9000
7000
5000
3000
1000
-1000
180 170 160 150 140 130 120 110 100 90 80 70 60 50 40 30
f1 (ppm)
